# Adapted motivational interviewing for brief healthcare consultations: A systematic review and meta‐analysis of treatment fidelity in real‐world evaluations of behaviour change counselling

**DOI:** 10.1111/bjhp.12664

**Published:** 2023-05-04

**Authors:** Alison K. Beck, Amanda L. Baker, Ben Britton, Alistair Lum, Sonja Pohlman, Erin Forbes, Lyndell Moore, Ditte Barnoth, Sarah J. Perkes, Chris Oldmeadow, Gregory Carter

**Affiliations:** ^1^ School of Medicine and Public Health The University of Newcastle Callaghan New South Wales Australia; ^2^ Hunter New England Health New Lambton New South Wales Australia; ^3^ Clinical Research Design and Statistical Service Hunter Medical Research Institute New Lambton Heights New South Wales Australia

**Keywords:** adapted motivational interviewing, behaviour change counselling, complex intervention, real world, systematic review, treatment fidelity

## Abstract

**Background:**

Behaviour change counselling (BCC) is an adaptation of motivational interviewing (MI) designed to maximize the effectiveness of time‐limited health behaviour change consultations. To improve intervention quality and understanding of treatment effects, it is recommended that evaluations of health behaviour change interventions incorporate existing fidelity frameworks (e.g. The National Institutes of Health [NIH] Behaviour Change Consortium) and ensure that treatment fidelity is assessed and reported.

**Purpose:**

This systematic review was designed to examine (a) adherence to NIH fidelity recommendations, (b) provider fidelity to BCC and (c) impact of these variables on the real‐world effectiveness of BCC for adult health behaviours and outcomes.

**Methods and Results:**

Searches of 10 electronic databases yielded 110 eligible publications describing 58 unique studies examining BCC delivered within real‐world healthcare settings by existing providers. Mean study adherence to NIH fidelity recommendations was 63.31% (Range 26.83%–96.23%). Pooled effect size (Hedges *g*) for short‐term and long‐term outcomes was .19 (95% CI [.11, .27]) and .09 (95% CI [.04, .13]), respectively. In separate, random‐effects meta‐regressions, neither short‐term nor long‐term effect sizes were significantly modified by adherence to NIH fidelity recommendations. For the subgroup of short‐term alcohol studies (*n* = 10), a significant inverse relationship was detected (Coefficient = −.0114, 95% CI [−.0187, −.0041], *p* = .0021). Inadequate and inconsistent reporting within the included studies precluded planned meta‐regression between provider fidelity and BCC effect size.

**Conclusions:**

Further evidence is needed to clarify whether adherence to fidelity recommendations modifies intervention effects. Efforts to promote transparent consideration, evaluation and reporting of fidelity are urgently needed. Research and clinical implications are discussed.

**Statement of contribution**

**What is already known?**

- Intervention fidelity is an important, but inadequately addressed methodological consideration in scientific evaluations of psychological interventions.
- Inadequate attention to fidelity limits current understanding of how interventions perform, especially within real‐world clinical settings
- This is the first systematic review and meta‐analysis to examine treatment fidelity in evaluations of behaviour change counselling, a brief adaptation of motivational interviewing

**What does this study add?**

- In real‐world healthcare consultations behaviour change counselling may produce significant, albeit modest improvements for a range of health behaviours and outcomes.
- Not enough studies assess, evaluate or report practitioner fidelity when delivering behaviour change counselling, so the impact of provider behaviour on client outcomes is unclear
- Improved understanding of intervention effectiveness within real‐world healthcare settings requires improved consideration, assessment and reporting of fidelity throughout the design and conduct of intervention evaluations.

## BACKGROUND

Fidelity is a complex construct that is central to the design, conduct, evaluation, reporting and dissemination of complex behaviour change research (Boutron et al., 2017; Craig et al., 2008; Michie et al., 2009). A range of definitions and models have been offered (Moore et al., 2015; O'Shea et al., 2016) and debate remains regarding how best to conceptualize this construct (Moore et al., 2015; Toomey et al., 2020). Broadly, treatment fidelity encompasses whether an intervention was delivered as intended (‘integrity’ or ‘adherence’), provider skill in delivering an intervention (‘competence’) and the degree to which an intervention is distinguishable from comparison conditions (‘differentiation’; Bellg et al., 2004; Borrelli et al., 2005; Kazdin, 1986; Moncher & Prinz, 1991). Treatment fidelity is central to interpreting the outcome of intervention evaluations, since the presence or absence of treatment effects can be more confidently attributed to the intervention under investigation (i.e. relative to non‐adherence and/or other factors unrelated to the intervention) (Dane & Schneider, 1998; Dobson & Cook, 1980; Meyer et al., 1993; Moncher & Prinz, 1991).

Demonstrating that promising interventions can be delivered adequately (i.e. with fidelity) by real‐world clinicians within real‐world settings (Onken et al., 2014) is a key task of Stage III Research, as defined by the National Institute of Health Stage Model for Behavioural Intervention Development (Onken et al., 2014). Specifically, when clinicians are trained to deliver an intervention within the context of every day service provision (i.e. often without the same control and resources afforded research settings; Miller & Rollnick, 2014), without assessing, monitoring and evaluating treatment fidelity, there is no way of knowing what is actually being delivered and the (in)consistency with the intervention under investigation. Simply put, “people cannot benefit from a treatment to which they have not been exposed” (Miller & Rollnick, 2014). Treatment fidelity is therefore a particularly important consideration for translational research (Miller & Rollnick, 2014; Onken et al., 2014).

In its broadest sense, the term ‘behaviour change counselling’ (BCC) has been used to refer to counselling efforts focused on promoting health behaviours and/ or changing unhealthy behaviours. Within the Motivational Interviewing (MI) literature, BCC refers to a time‐limited adaptation of MI that extends beyond brief advice. BCC has been defined as a way of ‘structuring a conversation which maximises the individual's freedom to talk and think about change in an atmosphere free of coercion and the provision of premature solutions’. (Rollnick et al., 1999, 2010) In this context (and the focus of the current review) BCC incorporates key principles, skills and strategies from MI to maximize the effectiveness of conversations about health behaviour change within the context of brief healthcare consultations (Rollnick et al., 2002). Although there is considerable overlap between MI and BCC, according to the seminal definition of this approach, a defining feature of BCC is delivery within time‐limited consultations (i.e. 5–30 min vs. 30–60 min for motivational interviewing or motivational enhancement; Rollnick et al., 2002).

The effectiveness of BCC has yet to be explored within a systematic review or meta‐analysis. Findings from related (and overlapping) ‘adaptions of MI’ (Burke et al., 2004) and MI more broadly are generally positive for promoting change in a range of health outcomes and behaviours (DiClemente et al., 2017; Frost et al., 2018; Lundahl et al., 2010, 2013; McKenzie et al., 2015; Morton et al., 2015). However, variable effect sizes and inconsistency in the direction of treatment effects have also been noted (Miller & Rollnick, 2012, 2014). Variations in treatment fidelity may be implicated (Miller & Rollnick, 2014). The benefits of MI may also be attenuated when evaluations move beyond controlled research settings to real‐world health care settings (Hallgren et al., 2018; Miller & Rollnick, 2014).

Making use of existing fidelity frameworks, and ensuring that treatment fidelity is not only assessed but also reported, represent two key recommendations for improving the quality of health behaviour change interventions (Toomey et al., 2020). The National Institutes of Health (NIH) Behaviour Change Consortium provides a framework for enhancing and evaluating treatment fidelity in health behaviour research (Bellg et al., 2004; Borrelli, 2011; Borrelli et al., 2005). This framework offers best practice recommendations across five domains (study design; provider training; treatment delivery; treatment receipt and treatment enactment). Of relevance to the methods employed in the current review, a corresponding checklist was also developed (Borrelli et al., 2005) and updated (Borrelli, 2011) to provide researchers with a tool for evaluating treatment fidelity (Borrelli et al., 2005). Although the NIH fidelity framework has been available since 2005, inadequate reporting of fidelity practices and outcomes (i.e. the level of fidelity achieved) within a range of complex health behaviour change interventions remains commonplace (Begum et al., 2021; Borrelli et al., 2005, 2015; Lambert et al., 2017; O'Shea et al., 2016; Preyde & Burnham, 2011; Rixon et al., 2016; Salloum et al., 2021; Walton et al., 2017). Whether this is the case for evaluations of BCC is unknown.

### Importance

Despite fidelity representing an important methodological consideration for the conduct and interpretation of translational research (Miller & Rollnick, 2014; Onken et al., 2014), and MI strategies and principles being widely utilized in real‐world clinical practice (Miller & Rollnick, 2014), there has yet to be a systematic evaluation of fidelity practices and outcomes within evaluations of BCC. Moreover, despite the role of fidelity in interpreting treatment effects, it is currently unclear whether the real‐world effectiveness of BCC is influenced by either (a) the degree to which a study incorporates NIH fidelity recommendations, or (b) provider fidelity to the delivery of BCC.

### Aims and objectives

This study is designed to examine treatment fidelity within real‐world evaluations of BCC for adult health behaviours or outcomes. Our objectives are to:

1. Conduct a systematic review to identify and summarize studies reporting evaluations of BCC interventions
2. For the identified studies, use the NIH behaviour change consortium fidelity checklist to estimate the degree to which NIH fidelity recommendations have been incorporated
3. Estimate the effect sizes for BCC using meta‐analysis
4. Examine the magnitude and direction of the relationship between NIH fidelity checklist score and intervention effect size using meta‐regression
5. Estimate provider fidelity to BCC (degree to which it has been delivered ‘as intended’) by synthesising reported levels of provider adherence and competence
6. Examine the magnitude and direction of the relationship between provider fidelity to BCC and intervention effect size using meta‐regression

### Review questions

This systematic review is designed to address the following questions:

1. What is the level of adherence (%) to the NIH framework for assessing, monitoring and enhancing treatment fidelity?
2. What is the effect size of BCC for short‐term (≤6 months) and long‐term (>6 months) primary health outcomes?
3. Is the effect size of BCC modified by how comprehensively a study addresses the NIH fidelity recommendations?
4. What is the pooled estimate for the level of treatment fidelity reported by the included studies?
5. Is the effect size of BCC modified by the degree to which the intervention is delivered ‘as intended’?

## METHODS

### Transparency and openness

This systematic review was prospectively registered PROSPERO 2019 CRD42019131169, informed by Cochrane (Higgins, Thomas, Chandler, Cumpston, Li, Page, & Welch, 2021a) and PRISMA (Moher et al., 2015) guidelines for conducting and reporting systematic reviews and meta‐analyses. The protocol has been published Beck, A.K., et al 2019 and is available from pubmed.ncbi.nlm.nih.gov/31366650/ (doi: https://doi.org/10.1136/bmjopen‐2018‐028417). This manuscript is reported according to the updated PRISMA statement (Page et al., 2021), and Journal Article Reporting Standards for Quantitative Research in Psychology (Appelbaum et al., 2018).

### Eligibility criteria

#### Study type

**FIGURE 1:**
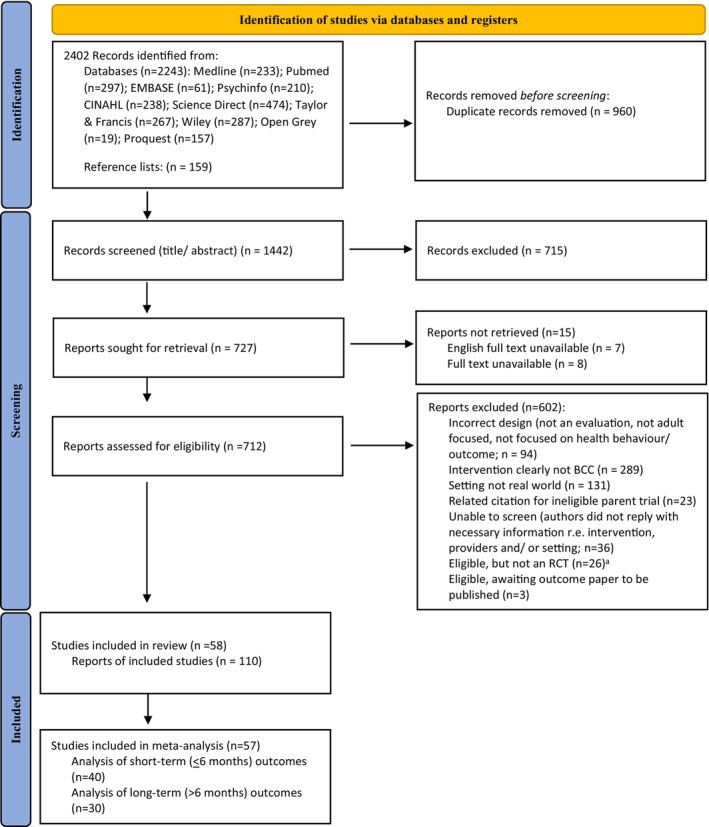
PRISMA flow diagram. ^a^In contrast to the published protocol, pragmatic constraints meant that non‐randomised designs were ultimately excluded from the current review.

To be classified as an ‘evaluation’ of BCC, the article had to assess the impact of BCC on one or more health behaviours or outcomes (defined below). Studies could be randomised (individual, cluster or stepped‐wedge), non‐randomised or observational. A full‐text English version of the article had to be available. No limits were set on study follow‐up duration. Following completion of searches, the number of studies identified and volume of data to extract, meant that we deviated from the protocol and only used randomised designs for data extraction and synthesis (see Figure 1).

#### Healthcare providers and participants

Existing health workers (paid or unpaid) within any real‐world health care setting or service had to deliver the BCC intervention to adults (18+) attending for healthcare (physical or mental). ‘Health worker’ was defined according to the World Health Organisation definition of “people engaged in actions whose primary intent is to enhance health” (World Health Organization, 2006) (a full list of professions is provided in the protocol; Beck, A.K. et al 2019).

#### Types of interventions

The intervention of interest was BCC, a time‐limited adaptation of MI (Rollnick et al., 2002, 2010). Studies were included if they defined the intervention being evaluated wholly or in part as ‘BCC’ per the following criteria (Rollnick et al., 2002; Box 1)

BOX 1

1. Use MI skills, principles and/ or strategies (i.e. MI or BCC were used and a published MI or BCC intervention framework or manual were cited).
2. Consist of more than “brief advice” (i.e. >5 min)
3. Brief (≤30 min)
4. Focus on helping participants to change a health behaviour

The BCC intervention could be of any intensity, session number and delivery mode (e.g. face‐to‐face, telephone etc.), and delivered alone, or as is common in clinical practice, in combination with other interventions.

#### Types of comparison conditions

The BCC intervention could be compared to active (e.g. psychological intervention and/ or pharmacotherapy) and/ or inactive (e.g. no treatment) or other control (wait list control, standard care, placebo) condition(s).

#### Types of outcome measures

Eligible studies evaluated the impact of the BCC intervention on patient health behaviour(s)/outcome(s). As it is common for fidelity methods and outcomes to be reported separately to the primary evaluation, consistent with published methodology (Borrelli et al., 2005), papers from eligible outcome evaluations that were cited for further information (e.g. training methods, intervention details) were also included (File S1).

### Information sources

#### Search strategy

Searches were conducted in 10 electronic databases (Medline, PubMed, EMBASE, PsycINFO, CINAHL Complete, Science Direct, Taylor and Francis, Wiley Online Library, Open Grey and Proquest). Search terms that described the intervention of interest (BCC) were combined with those that described the target population (health workers) and setting (real‐world settings/services). See File S2 for the full MEDLINE search strategy.

Abstract, title, key words and/or subject headings specific to each of the identified databases were searched. Searches were run from the inception of each database until present. Searches were first conducted (by AKB) in November 2018 and re‐run in July 2020. Reference lists of eligible papers were hand‐searched and potentially relevant articles were sourced, including any citations to previously published fidelity methods and/or outcomes. All publications were organised in Endnote and the systematic review management software Covidence (Covidence Systematic Review Software, n.d.).

### Study records

#### Selection process

The PRISMA 2020 flow diagram is presented in Figure 1. AKB reviewed the titles and abstracts from the searches and excluded articles if they clearly ineligible. If eligibility was unclear from the abstract the full text was accessed and independently assessed against the inclusion and exclusion criteria by two review authors (AKB and EF). Discrepancies were resolved via discussion. If insufficient information was reported to determine whether inclusion criteria were met (e.g. intervention duration, setting, provider and content) the authors of that study were contacted on no more than three occasions to obtain further information. In the absence of sufficient information to determine eligibility, articles were not retained for data extraction.

### Data collection process

Data extraction and coding were performed by AKB and independently by a second researcher (AL, SP, LM, DB and SJP). Multiple publications arising from the same study were extracted separately and combined across data collection forms.

#### Data items

Extraction forms were developed by the research team. They were informed by Cochrane guidelines (Higgins, Thomas, Chandler, Cumpston, Li, Page, & Welch, 2021a) and the latest iteration (Borrelli, 2011) of the NIH treatment fidelity checklist (Borrelli et al., 2005) and included information about the article, risk of bias, setting, intervention methods, fidelity methods, fidelity outcomes (e.g. adherence or competence), primary outcome and treatment effects.

#### Incorporation of NIH fidelity recommendations

Aligned with published recommendations (O'Shea et al., 2016) and the methodology of related reviews (e.g. Lambert et al., 2017; Salloum et al., 2021; Walton et al., 2017) the fidelity practices of included studies were assessed using the latest iteration (Borrelli, 2011) of the NIH treatment fidelity checklist (Borrelli et al., 2005). This 40‐item checklist assesses the presence/absence of fidelity practices across five domains: study design, provider training, treatment delivery, treatment receipt and treatment enactment (see Bellg et al., 2004; Borrelli, 2011; Borrelli et al., 2005 for further information). Consistent with published modifications (Ang et al., 2018), we differentiated the ‘present’ rating into ‘present sufficiently’ (to denote detailed or extensive coverage/ reporting) and ‘present insufficiently’ (for information that is brief, reasonably inferred based on the information presented or inadequate to fulfil criteria for ‘sufficient’).

#### Level of provider fidelity reported

Any data pertaining to the level of provider fidelity reported by the included studies were extracted. This included outcomes from observer, provider and participant‐rated instruments used to assess provider fidelity to the delivery of the BCC intervention, and any comparisons between treatment arms (i.e. differentiation).

### Study quality assessment

The methodological quality of included studies was assessed using the Effective Public Health Practice Project (EPHPP) qualitative assessment tool for quantitative studies (EPHPP, 1998). This quality appraisal tool is composed of eight sections (selection bias, study design, confounders, blinding, data collection methods, withdrawals and dropouts, intervention integrity and analysis). Ratings (strong vs. moderate vs. weak) within each domain are used to generate an overall quality rating for the study (strong vs. moderate vs. weak). The EPHPP demonstrates sound psychometric properties (Armijo‐Olivo et al., 2012) and was selected as it measures a distinct, albeit related concept (quality) than the Cochrane Collaboration risk of bias tool.

### Risk of bias assessment

Aligned with Cochrane recommendations (Higgins, Thomas, Chandler, Cumpston, Li, Page, & Welch, 2021a) risk of bias (within and across studies) was assessed using the Cochrane Collaboration risk of bias tool for randomised studies (RoB 2.0). Each item was judged as being high, low or unclear risk, in line with published guidance (Higgins, Thomas, Chandler, Cumpston, Li, Page, & Welch, 2021a). All eligible randomised studies were included in the meta‐analysis irrespective of risk of bias assessment.

### Assessment of the quality of evidence of effects

Aligned with Cochrane recommendations the Grading of Recommendations, Assessment, Development, and Evaluations (GRADE) approach (Higgins, Thomas, Chandler, Cumpston, Li, Page, & Welch, 2021a) was used to assess the quality of evidence for the short‐term and long‐term effects of BCC (overall and according to intervention target behaviour). Quality of evidence for each outcome is presented according to the following categories: ‘high’, ‘moderate’, ‘low’, ‘very low’, in line with published definitions (Higgins, Thomas, Chandler, Cumpston, Li, Page, & Welch, 2021a).

### Statistical analysis

Analyses were conducted using SPSS (IBM Corp, 2017) and Comprehensive Meta‐Analysis (CMA; Borenstein et al., 2013).

#### Incorporation (%) of NIH fidelity recommendations

The degree to which each article incorporated the NIH fidelity recommendations (overall, and within each of the five fidelity domains) was calculated by dividing the number of strategies deemed appropriate for that article by the number of strategies coded as ‘present’ (either ‘present sufficiently’ or ‘present insufficiently’). These scores were then used to calculate the mean proportion of adherence to fidelity recommendations (overall, and within each of the five fidelity domains) across all included studies. ‘High adherence’ to the NIH fidelity recommendations was defined as ≥80% of the total number of applicable checklist items coded as ‘present’ (Borrelli, 2011; Borrelli et al., 2005). To determine the use of specific fidelity recommendations across studies, the number of studies using each checklist item was calculated as a percentage of the total number of articles for which that item was deemed applicable. We also examined whether the degree to which NIH Fidelity recommendations were incorporated changed over time (File S3).

#### Effect size of BCC

Quantitative data from included studies (number of events, number of non‐events, means, standard deviations, confidence intervals, change scores, effect sizes, *t* tests, *F* tests and/or *p* values) were imported into CMA (Borenstein et al., 2013) and converted to a standardised score (Hedge's *g*). Events and non‐events were defined in relation to the data presented for each dichotomous primary outcome extracted (e.g. ‘events’ for positively framed outcomes such as number of people abstinent, and ‘non‐events’ for negatively framed outcomes such as the number of people who continued to smoke/ drink etc.). When necessary values were not reported in the primary study (e.g. standard deviation), they were generated from the available data (e.g. standard error) using published conversion formulas (Higgins, Thomas, Chandler, Cumpston, Li, Page, & Welch, 2021b).

##### Unit of analysis issues

###### Cluster randomised trials

To avoid ‘unit of analysis error’ (whereby studies that ignore clustering receive greater weight than appropriate in the analysis) (Hedges, 2009; Higgins, Thomas, Chandler, Cumpston, Li, Page, & Welch, 2021c) we followed Cochrane guidelines for including cluster designs. The majority of the included cluster‐randomised trials (21/24) explicitly accounted for clustering in the reported analyses and these adjusted values (means, standard deviations, *p* values) were used for the calculation of effect sizes. Otherwise, we used a conservative intraclass correlation coefficient (ICC) of .5 (Hedges, 2009) and generated adjusted values (Higgins, Thomas, Chandler, Cumpston, Li, Page, & Welch, 2021c).

###### Studies with more than two arms

Two studies (reported across three outcome papers) included more than one treatment arm meeting criteria for BCC (Aalto et al., 2000, 2001; Garner et al., 2020). To avoid double counting participants or arbitrary omission of relevant groups, we followed Cochrane guidelines (Higgins, Thomas, Chandler, Cumpston, Li, Page, & Welch, 2021b) to combine the eligible BCC arms within each of these studies. As BCC was the intervention of interest, and treatment as usual (or equivalent) was the most common comparator condition, additional active treatment arms from three‐arm trials (Cook et al., 2017; Ershoff et al., 1999; Meyer et al., 2012) were not included in the meta‐analysis. One study comparing a BCC intervention to various doses of MI (with or without pharmacotherapy; Hollis et al., 2007) was also excluded from the meta‐analysis due to the absence of a treatment as usual (or equivalent) comparator condition.

###### Studies with multiple follow‐up intervals

When several follow‐up time‐points were reported, as per the Cochrane Convention for classifying follow‐up intervals, outcomes were defined a priori as being short‐term (≤6 months) or long‐term (>6 months) and separate analyses were performed. When multiple ‘short’ or ‘long’ time points were reported within a single study (e.g. end of treatment, 2‐month and 6‐month) the longest interval (i.e. 6‐months) was selected for analysis.

###### Other

One study reported the primary outcome of quality of life across four domain scores (Hegarty et al., 2013). To avoid double counting participants, or arbitrarily excluding this study domain scores were combined (Higgins, Thomas, Chandler, Cumpston, Li, Page, & Welch, 2021) into a single aggregate value for analysis.

##### Heterogeneity and publication bias

Heterogeneity in effect sizes was assessed using *Q*, *I* (Michie et al., 2009), T (Michie et al., 2009) and *T*. Publication bias was assessed through visual inspection of the random‐effects funnel plot, Begg and Mazumdar rank correlation, Egger's regression intercept and Duval and Tweedie's trim and fill.

##### Data synthesis

Random‐effects meta‐analyses were performed separately on the short‐term (*n* = 40) and long‐term (*n* = 30) primary study outcomes. Included papers were grouped according to the target of the intervention and subgroup analyses were performed (pooling *T*; Michie et al., 2009; across groups to improve accuracy; Borenstein, 2019). Effect sizes are reported as Hedges' *g* ± 95% CIs. To understand what the expected range of true effects for populations similar to those in the analysis is, we also calculated the prediction interval (using CMA Prediction Intervals software). As the mean effect size in random‐effects analysis is too narrow when based on the Z‐distribution, the Knapp‐Hartung adjustment was applied to yield a wider (and more accurate) confidence interval (Borenstein, 2019).

### Relationship between NIH fidelity checklist score (%) and intervention effect size

Random‐effects meta‐regressions were performed to examine the magnitude and direction of effect between NIH fidelity checklist score (%) and intervention effect size. Separate analyses were performed on the short‐term (*n* = 40) and long‐term (*n* = 30) study outcomes. The only subgroup with sufficient papers for meaningful meta‐regression (≥10) were interventions targeting alcohol use that reported short‐term study outcomes (*n* = 10). Given the level of heterogeneity, follow‐up sensitivity analyses were conducted to examine the magnitude and direction of effect between NIH fidelity checklist score (%) and intervention effect size when only those studies demonstrating ‘high‐fidelity’ to the NIH checklist were included (*n* = 9 short‐term and *n* = 5 long‐term).

### Pooled estimate of fidelity of intervention delivery

There was wide variation in how fidelity was defined, assessed and reported, with fidelity outcomes often idiosyncratic to the intervention under evaluation. We were therefore unable to generate a pooled estimate of fidelity to BCC. Instead, we provide a narrative synthesis of the assessment and reporting of fidelity of intervention delivery (File S4).

### Relationship between fidelity of intervention delivery and intervention effect size

As we were unable to generate a pooled estimate of fidelity to BCC, the planned random‐effects meta‐regression was not conducted.

## RESULTS

One hundred and 10 publications describing 58 unique studies were included. Patient outcomes were evaluated and reported in 61 papers. A further 49 papers were sourced for additional information about fidelity (including 18 study protocols; 16 papers cited for information about study design, training, intervention and/ or fidelity; and 16 process analyses conducted wholly, or in part to evaluate fidelity outcomes). Key study characteristics are summarised in Table S1. The majority of studies were conducted in the USA (*n* = 19/58; 32.7%), United Kingdom (*n* = 9/58; 15.5%) and the Netherlands (*n* = 6/58, 10.3%). Half were conducted in primary care settings (*n* = 29/58; 50%), with the remainder distributed between hospital in‐patient and/ or out‐patient settings (*n* = 14/58; 24.1%) and specialist services/ clinics (*n* = 15/58; 25.8%). Studies used BCC to target a range of health behaviours including alcohol (*n* = 11), smoking (*n* = 9), substance use (*n* = 6), multiple health behaviour change (including diet, activity, lipid control, alcohol and/ or smoking; *n* = 11), physical activity (*n* = 5), sub‐optimal glycaemic control (*n* = 3), treatment engagement/ adherence (*n* = 7), and other health behaviours (*n* = 6; including oral hygiene, HIV risk behaviours, safety planning for intimate partner violence and oncology nutrition).

### Incorporation (%) of NIH fidelity recommendations

Use of the NIH fidelity recommendations within and across studies is presented in Table 1. Mean adherence to the NIH fidelity checklist was 63.31% (Range = 26.83%–96.23%; Median = 63.41, IQR = 51.19%–75.60%). Use of individual checklist items varied widely across studies (8.62%–98%). Ten studies (Bóveda‐Fontán et al., 2015; Britton et al., 2019; Darker et al., 2016; D'Onofrio et al., 2008; Fisher et al., 2014; Garner et al., 2020; George et al., 2021; Hegarty et al., 2013; Shin et al., 2013; Zatzick et al., 2014) distributed across evaluations of BCC targeting ‘other’ health behaviours (3/6, 50%), substance use (2/6, 33%), alcohol (3/11, 27%), physical activity (1/5, 20%), treatment adherence (1/7, 14%) and multiple health behaviour change (1/11, 9%) addressed more than 80% of NIH fidelity checklist items. None of the evaluations targeting smoking or suboptimal glycaemic control attained this benchmark. Fidelity considerations encompassed under the ‘design’ domain were most frequently addressed, with an average of 73.72% of items addressed to some degree. This was followed by ‘receipt’ (60.68%), ‘delivery’ (57.08%), ‘training’ (54.52%), and finally ‘enactment’, with an average of 37.93% adherence to this domain. Average adherence to each of the five NIH fidelity domains is summarised in Figure S1.

**TABLE 1:**
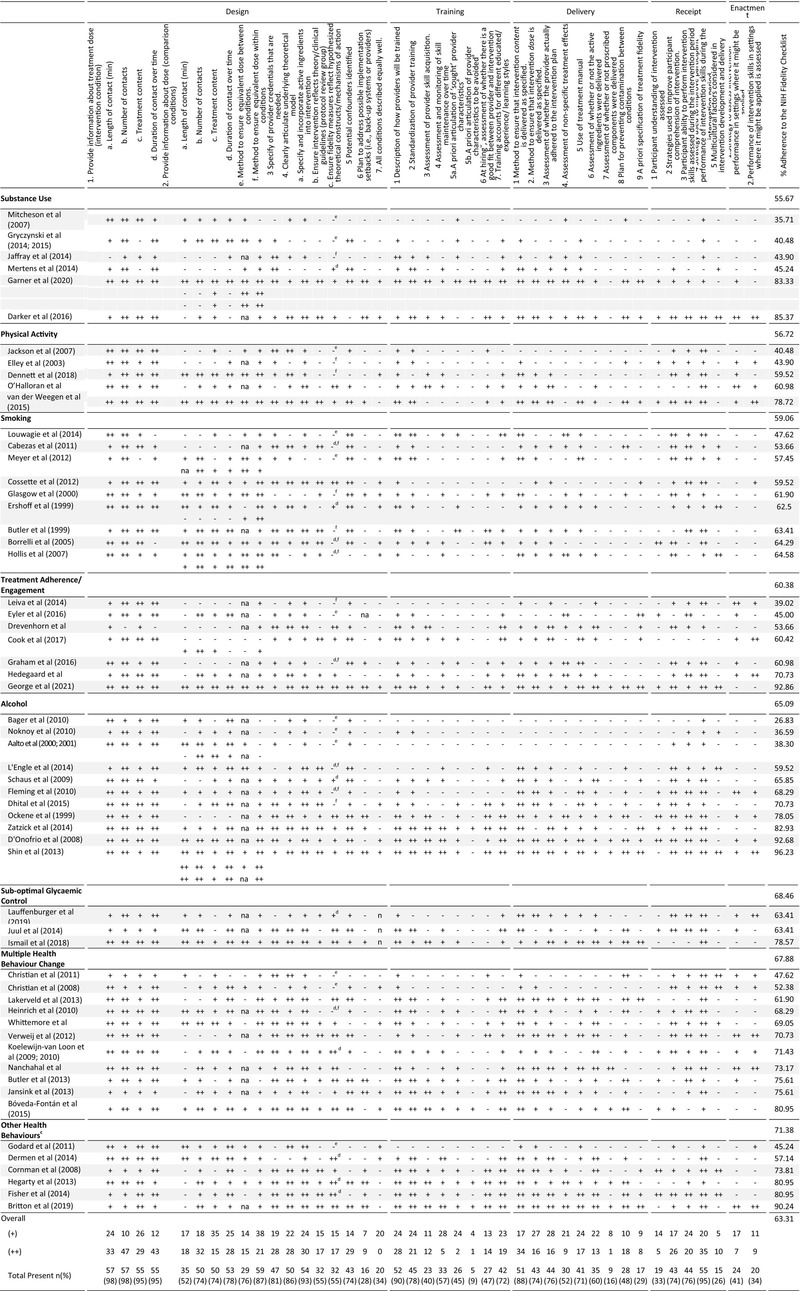
NIH fidelity checklist ratings within and across studies, grouped according to the health behaviour(s) targeted by the BCC intervention and fidelity checklist score (lowest to highest)

^a^ This item was split into two for rating to capture differences between reporting of ‘sought’ after & ‘avoided’ provider characteristics.

^b^ As this review was conducted on real‐world articles whereby providers were already working within the service, this item was rated based on the overall study methodology.

^c^ Other target health behaviours include oral hygiene (*n* = 2), HIV risk behaviours (*n* = 2), oncology nutrition (*n* = 1) and safety planning for intimate partner violence (*n* = 1).

^d^ Findings not reported.

^e^ No fidelity assessment.

^f^ Fidelity assessment, but no rationale.

### Effect size of BCC

#### Short‐term (≤6 months) study outcomes

**FIGURE 2:**
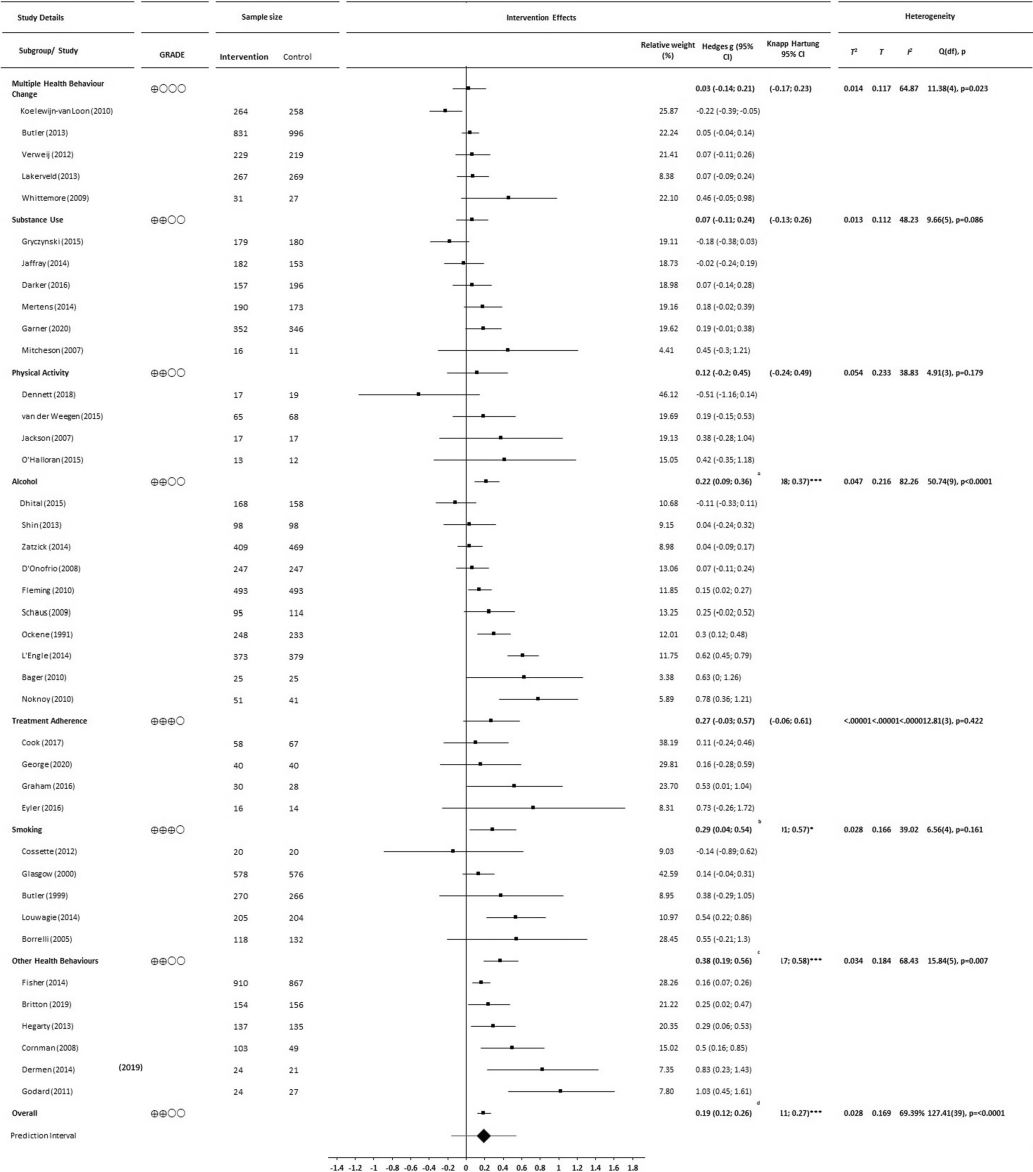
Forest plot summarising effect size (Hedges g), confidence intervals, prediction interval and heterogeneity statistics for studies included in the analysis of short‐term (≤6 months) outcomes (*n* = 40), presented according to target health behaviour and effect size (smallest to largest) (2019). ^a^(*Z* = 3.344, *p* = .001; *t*(33) = 3.11, *p* = .0038); ^b^(*Z* = 2.290, *p* = .022; *t*(33) = 2.13, *p* = .0406); ^c^(*Z* = 3.939, *p* < .001; *t*(33) = 3.67, *p* = .0009); ^d^(*Z* = 5.133, *p* < .001; *t*(39) = 4.61, *p* < .001)

The meta‐analysis of short‐term outcomes is summarised in Figure 2. For short‐term outcomes, the standardised difference in means was .19 (95% CI .11–.27). On average, those who received the BCC intervention scored .19 standard deviations higher (better health behaviour or outcome) up to 6 months post‐baseline than those who did not (Z = 5.13, *p* < .0001; *t* = 4.61, *df* = 39, *p* < .0001). The prediction interval ranged from −.16 to .54, indicating that the impact of the intervention varied widely, and in a small minority of populations may have been unhelpful. Substantial levels of heterogeneity were observed (*Q*, *I* ^2^, *T* ^2^ and *T* are summarised on Figure 2), with >60% of variance in observed effects due to variation in the true effects (rather than sampling error). We found evidence to suggest that the effect size was larger in smaller studies (Begg and Mazumdar rank correlation test *p* = .09; Egger regression test = .01; Figure S2). The GRADE rating is indicative of ‘low’ certainty in the demonstrated short‐term impact of BCC.

#### Long‐term (>6‐month follow‐up) study outcomes

**FIGURE 3:**
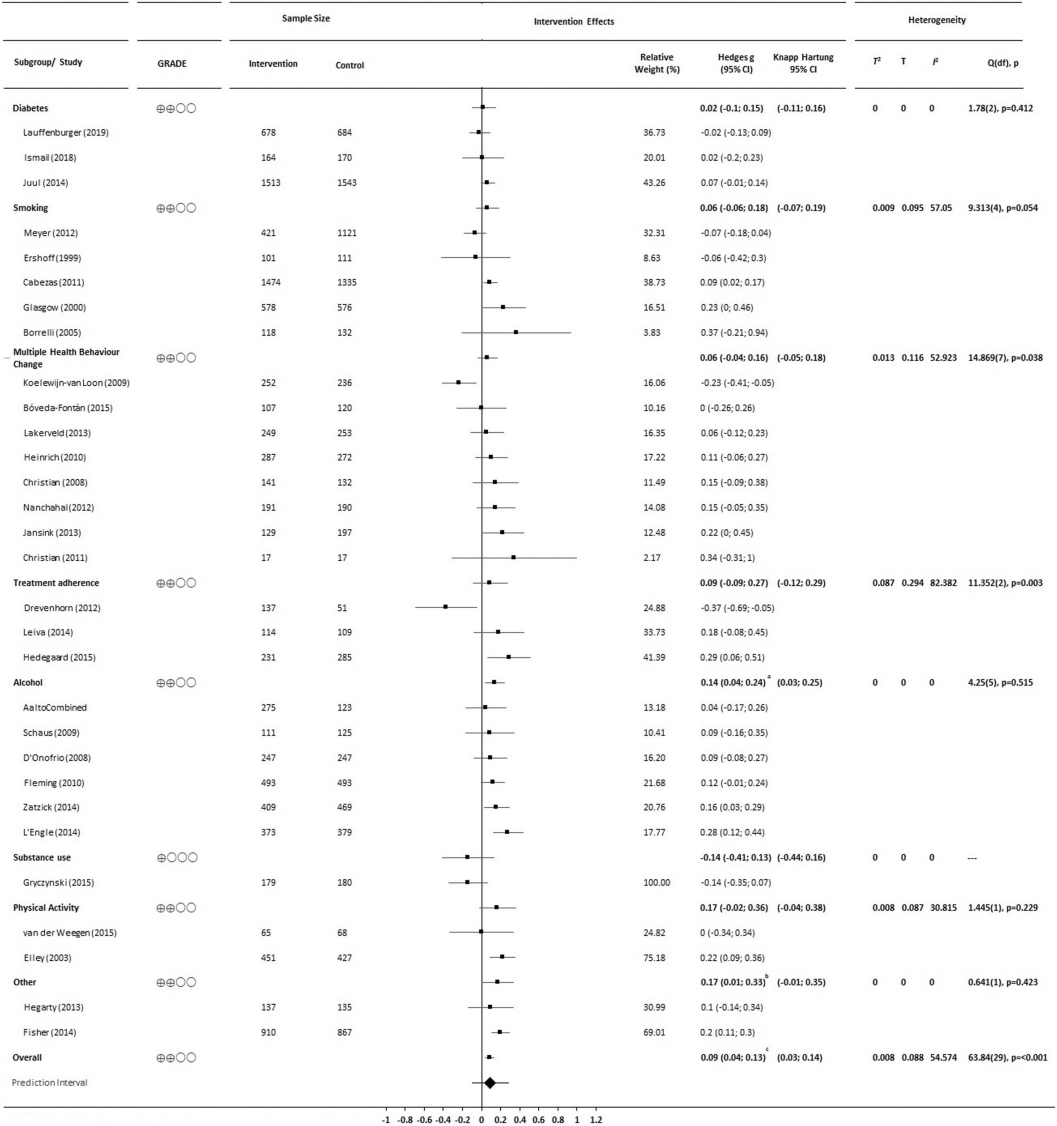
Forest plot summarising effect size (Hedges g), confidence intervals, prediction interval and heterogeneity statistics for studies included in the analysis of long‐term (>6 months) outcomes (*n* = 30), presented according to target health behaviour and effect size (smallest to largest). ^a^(*Z* = 2.74, *p* = .006; *t*(22) = 2.59, *p* = .0168); ^b^(*Z* = 2.04, *p* = .042; *t*(22) = 1.92, *p* = .0676); ^c^(*Z* = 3.55, *p* < .001; *t*(29) = 3.41, *p* = .0019)

The analysis of long‐term outcomes is summarised in Figure 3. For long‐term outcomes, the standardised difference in means was .09 (95% CI .04–.13). On average, those who received the BCC intervention scored .09 standard deviations higher (better health behaviours or outcomes) on follow‐ups conducted between eight and 36 months post‐baseline than those who did not (Z = 3.55, *p* < .0001; *t* = 3.41, *df* = 29, *p* = .0019). The prediction interval for the overall effect size ranged from −.10 to .27. As with short‐term findings, the impact of the intervention varied, and in a small minority of populations may have been unhelpful. Heterogeneity was considerable (*Q*, *I* (Michie et al., 2009), *T* (Michie et al., 2009) and *T* are summarised in Figure 3), with over half of the variance in observed effects due to variation in the true effects (rather than sampling error). We did not find evidence that effect size was larger in smaller studies (Begg and Mazumdar rank correlation test *p* = .33; Egger regression test = .48; Figure S3). The GRADE rating is indicative of ‘low’ certainty in the demonstrated long‐term impact of BCC.

### Relationship between NIH fidelity checklist score (%) and intervention effect size

We cannot conclude that the NIH fidelity checklist score is related to the overall effectiveness of BCC for either short‐term (Q(1) = 3.47, *p* = .0626; Knapp‐Hartung F(1, 38) = 2.89, *p* = .10975) or long‐term (Q(1) = .38, *p* = .5374; Knapp‐Hartung F(1, 28) = .34, *p* = .5620) study outcomes (Figure S4). For the subgroup of studies examining the impact of BCC on short‐term alcohol outcomes, an inverse relationship (coefficient = −.0114, *SE* = .0037, 95% CI −.0187 to −.0041) was detected between the NIH fidelity checklist score and effect size (Q(1), *p* = .0021; Figure S4). This relationship remained significant after applying the Knapp‐Hartung adjustment (F(1, 8) = 9.45, *p* = .0153; Figure S4). In follow‐up sensitivity analyses conducted using only those studies that attained ‘high‐fidelity’ to the NIH fidelity checklist, no significant relationship was detected between NIH fidelity checklist score and the overall effectiveness of BCC for either short‐term (Q(1) = .70, *p* = .4043; Knapp‐Hartung *F*(1, 7) = .70, *p* = .5321) or long‐term (Q(1) = .58, *p* = .4480; Knapp‐Hartung *F*(1, 3) = .58, *p* = .5032) study outcomes.

### Provider fidelity to BCC

The assessment and reporting of fidelity of intervention delivery are summarised in Table S2, and a narrative synthesis is presented in File S4. Forty‐six studies (79.31%) reported using methods to evaluate the fidelity of intervention delivery, of which 28 (60%) subsequently presented the outcome data. Studies varied widely in the methods used and how the findings were reported.

### Quality assessment

EPHPP quality assessment ratings are presented in Figure S5. Almost three quarters of included outcome papers were rated to be of moderate (27/61, 44.26%) or strong (18/61, 29.50%) quality. All outcome papers were assigned a rating of ‘strong’ for study design (all were RCTs). Otherwise, studies were most likely to be assigned a rating of ‘strong’ for identifying and controlling for confounders (45/61, 73.77%). Selection bias and blinding were the least likely domains to be assigned a rating of ‘strong’ (both 8/61, 13.11%).

### Risk of bias

Risk of bias ratings is presented in Figures S6 and S7. Approximately three‐quarters of studies were assigned a risk of bias rating of either ‘low’ (5/58, 8.62%) or ‘some concerns’ (38/58, 65.51%). Across the included studies, risk of bias due to missing outcome data was lowest (44/58, 75.86% ‘low’ rating) and deviation from the intended intervention highest (11/58, 18.96% ‘high’ rating).

### Evidence of short‐ and long‐term effects

GRADE ratings for strength of evidence for short‐term and long‐term benefits of BCC (intervention subgroups and overall) are presented in Figures 2 and 3, respectively. Evidence for the benefit of BCC was oftentimes low, at best moderate (for short‐term gains following interventions targeting either smoking or treatment adherence) and at worst very low (for short‐term gains for interventions targeting multiple health behaviour change and long‐term gains for interventions targeting substance use).

## DISCUSSION

The average level of adherence to the NIH framework for assessing, monitoring and enhancing fidelity was 63.31% (Range = 26.83%–96.23%). This finding is 6%–26% higher than previously published levels of adherence to the NIH framework (Begum et al., 2021; Borrelli et al., 2005; Lambert et al., 2017; O'Shea et al., 2016; Preyde & Burnham, 2011; Salloum et al., 2021). Ten studies (17%) (Bóveda‐Fontán et al., 2015; Britton et al., 2019; Darker et al., 2016; D'Onofrio et al., 2008; Fisher et al., 2014; Garner et al., 2020; George et al., 2021; Hegarty et al., 2013; Shin et al., 2013; Zatzick et al., 2014) addressed more than 80% of the fidelity checklist items, also higher than published accounts (Begum et al., 2021; Borrelli et al., 2005; Preyde & Burnham, 2011; Salloum et al., 2021). Our systematic review is unique in that we specifically focused on evaluations conducted within the context of real‐world service provision. Given that efforts to enhance, monitor and evaluate fidelity are especially important for evaluations conducted outside of highly controlled research settings (Miller & Rollnick, 2014; Onken et al., 2014), this demonstrated attention to fidelity is encouraging. However, given that we, and others (Begum et al., 2021; Borrelli et al., 2005; Lambert et al., 2017; O'Shea et al., 2016; Preyde & Burnham, 2011; Salloum et al., 2021) found wide variation in the use of fidelity strategies within and across the five NIH fidelity domains, identifying the relative clinical importance of the various strategies remains an important question for future research. Indeed, ‘design’ considerations were most likely to be attended to, relative to other aspects of fidelity (e.g. training and delivery) that are presumably key in terms of the quality of the intervention delivered, and therefore any effect on outcome. Clearly, further research is warranted.

The standardised effect size for BCC varied according to intervention target and follow‐up duration. Minimal short‐term benefit was found for interventions targeting multiple health behaviour change, substance use and physical activity. A small effect size was detected for interventions targeting alcohol, treatment adherence, smoking and ‘other’ health behaviours. We found only a small pooled effect size for the long‐term benefits of BCC. Published effect sizes for MI interventions targeting health behaviours vary widely (DiClemente et al., 2017; Hallgren et al., 2018), with reports ranging from minimal to moderate benefit (An & Song, 2020; Chen et al., 2022; Makin et al., 2021; Yao et al., 2021; Zomahoun et al., 2017). Consistent with the current findings, the benefits may attenuate over time (Lundahl & Burke, 2009), and be larger for smoking and alcohol use relative to other health behaviours (DiClemente et al., 2017; Frost et al., 2018). Of note, evidence suggests that larger effect sizes may be produced when MI is compared to ‘weak’ comparison groups (e.g. waitlist or no treatment; Frost et al., 2018; Lundahl & Burke, 2009). We, and others (Begum et al., 2021; Preyde & Burnham, 2011) have found that the content of comparator conditions is typically not assessed or described. Accordingly, small effects may be due to lack of differentiation between intervention and comparator conditions. Improved attention to the description, assessment and reporting of comparator conditions is therefore essential. It is also important to acknowledge that effect size will also be influenced by the outcome measure selected and corresponding follow‐up duration, as some health behaviours may have as an outcome, variables that take longer to change.

We did not detect a significant relationship between incorporation of NIH fidelity recommendations and overall effect size for either short‐ or long‐term outcomes. In interventions targeting alcohol use, an inverse relationship was detected for short‐term outcomes. Although not significant (*p* = .6), we also observed a trend for an inverse relationship between incorporation of NIH fidelity recommendations and short‐term outcomes in the overall analysis. Aligned with our findings, prior systematic reviews have found smaller effect sizes for studies that conducted fidelity checks (irrespective of actual level of fidelity) relative to those that did not (Barrett et al., 2018; Palacio et al., 2016). An inverse relationship has also been detected between MI effect size and methodological rigour more broadly (Burke et al., 2003). One interpretation of our findings then, is that greater incorporation of NIH fidelity recommendations may be found in studies that reduce systematic errors more consistently and so avoid otherwise artificially inflated treatment effects. However, in light of the heterogeneity within our sample, and that the NIH checklist was not developed specifically for interventions evaluated under real‐world conditions, further research is needed before definitive conclusions can be drawn.

The inconsistency of how fidelity was assessed and reported within the included publications meant that an overall estimate of provider fidelity to the delivery of BCC could not be calculated. Limited consideration of fidelity within the current sample of papers may be due to the more pragmatic nature of these evaluations. For example, in addition to the practical challenges of assessing and monitoring fidelity within the context of real‐world clinical practice (Gill et al., 2020; Hurlocker et al., 2020; Ogden, 2016; Rayson et al., 2022; Zuidgeest, Goetz, et al., 2017; Zuidgeest, Welsing, et al., 2017) fidelity may receive less attention within this context due to a shift in focus from efficacy towards effectiveness (Thorpe et al., 2009). However, irrespective of where a study lies on the efficacy‐effectiveness continuum, without information about the degree to which an intervention is delivered ‘as intended’, and the (dis)similarity to the comparison condition(s), it remains unclear whether null effects are due to an ineffective intervention, or poor implementation (Gottfredson et al., 2015; Heather, 2014). To this end we identified several challenges that need to be addressed within future evaluations. Firstly, aligned with prior reviews (e.g. Copeland et al., 2015; Dalgetty et al., 2019; Lindson et al., 2019; O'Halloran et al., 2014; Rimayanti et al., 2022; Spencer & Wheeler, 2016; Steed et al., 2019) there was considerable discrepancy between the proposed or actual assessment of fidelity (*n* = 48) and subsequent reporting (*n* = 26) of fidelity outcomes. This selection bias not only limits the data that is available but also the generalisability of the findings. Secondly, although 30 studies adhered to ‘best practice’ (Bellg et al., 2004; Borrelli, 2011; Borrelli et al., 2005) by using an observer‐rated instrument to evaluate fidelity, also consistent with prior reviews (Copeland et al., 2015; Dalgetty et al., 2019; Lindson et al., 2019; O'Halloran et al., 2014; Rimayanti et al., 2022; Spencer & Wheeler, 2016; Steed et al., 2019), the methods were poorly described. Information about the sample size rated, how the sample was selected, how the coders were trained, how inter‐rater reliability was assessed (and the level achieved) and whether raters were blind are important methodological considerations for reducing bias and measurement error that were frequently omitted. Thirdly, although BCC was the intervention of interest, fidelity checks largely focused on other intervention components. This has significant implications for our understanding of the relative contribution of BCC to patient outcome. Consistent with published evidence (e.g. Lambert et al., 2017; Olson, 2015; Walton et al., 2017) evaluation of fidelity within the included studies also often relied on idiosyncratic outcomes and assessment instruments (e.g. intervention checklists). This is despite the availability of a range of validated, observer‐rated instruments for assessing fidelity to MI and BCC (Gill et al., 2020; Hurlocker et al., 2020) and standardised taxonomies for operationalising (Kok et al., 2016), and coding (Knittle et al., 2020; Michie et al., 2013) the delivery of behaviour change techniques.

Inspection of individual study findings suggests wide variation in treatment fidelity, from minimal through to proficient delivery of BCC/MI. Within‐ and between‐provider variation in fidelity to MI and other behaviour change interventions is not uncommon (Bateup et al., 2020; Beckman et al., 2021; Hallgren et al., 2018).Indeed, provider delivery of an intervention can be influenced by a range of factors, including provider (e.g. personality; Fletcher & Delgadillo, 2022) and client (e.g. motivation; Imel et al., 2011) variables, and their interaction (Borsari et al., 2019; Magill et al., 2018; Villarosa‐Hurlocker et al., 2019). The ideal benchmark for treatment fidelity has been set at 80% (Borrelli et al., 2005). However, the feasibility of attaining this benchmark within real‐world clinical practice has been questioned (Hankonen, 2021). The complex interplay between treatment fidelity and ‘therapist responsiveness’ (Stiles & Horvath, 2017; Webb et al., 2010) is an important consideration. Therapist responsiveness refers to the fact that providers often adapt their behaviour according to the perceived needs of the patient (Stiles & Horvath, 2017; Webb et al., 2010). Accordingly, rigid adherence in the service of ‘high fidelity’ may undermine intervention effectiveness by failing to account for important contextual factors (Chambers et al., 2013; Lundahl & Burke, 2009). Conversely, accumulating evidence suggests that adapted interventions and therapist flexibility (while staying true to core intervention components and hypothesised mechanisms of action) may enhance intervention effects (Stirman et al., 2017; Sundell et al., 2015). Efforts should therefore focus on maximising the likelihood that the decision of whether or not to deliver a given intervention component is theoretically informed and clinically driven (i.e. relative to provider preference or avoidance). Supervision and feedback are therefore essential (Caron & Dozier, 2019, 2021; Isenhart et al., 2014; Naar et al., 2021; Schwalbe et al., 2014). Incorporating objective self (Beckman et al., 2021; Caron & Dozier, 2021; Isenhart et al., 2014) and peer (Beckman et al., 2021; Isenhart et al., 2014) ratings of patient consultations into the feedback process may be particularly beneficial, and double as an opportunity to address the need (Fordham et al., 2021; Gill et al., 2020; Hurlocker et al., 2020) for novel, feasible methods for evaluating fidelity within the context of real‐world service provision.

In the absence of a pooled estimate of fidelity, we are unable to comment on whether the short‐ or long‐term benefits of BCC are in any way modified by the degree to which the intervention was delivered ‘as intended’. Evidence regarding the relationship between treatment fidelity and patient outcome is limited and complex. For example, adherence to BCC and MI have been variously linked to improved patient outcomes (DeVargas & Stormshak, 2020; Fischer, 2016; Spohr et al., 2016), worse patient outcomes (Beck et al., 2021; DeVargas & Stormshak, 2020; Osilla et al., 2018; Schmidt et al., 2019) and no effect (Osilla et al., 2018; Rowell, 2019; Spohr et al., 2016; Webb et al., 2010). Moreover, the relationship between fidelity and patient outcome has been found to vary according to intervention component (Copeland et al., 2015; Magill et al., 2018; Pace et al., 2017), provider skill (Borsari et al., 2019), and client severity (Imel et al., 2011). Further research is warranted to clarify the relationship between patient and clinician variables, intervention delivery and treatment outcome. Recent reports (Stirman et al., 2017) suggest that adaptive and factorial approaches to clinical trials, such as SMART (Sequential Multiple Assignment Randomized Trial), and Multiphase Optimization Strategy (MOST) may be key to understanding the adaptations that occur in clinical practice and their impact on treatment outcome.

### Limitations

Several limitations should be considered when interpreting our findings. Firstly, searches were limited to studies published in English. Secondly, although studies from a range of countries were included, most were conducted in the USA, UK and other high‐income countries. Thirdly, interventions were mostly delivered by GPs and/ or nurses within the context of primary care and providers tended to be female. Given that the delivery of MI may vary according to country (Schmidt et al., 2022), profession (Cook et al., 2016), and that gender differences in patient‐centred communication have been noted (Jefferson et al., 2013), generalisation beyond the current sample is unclear. Thirdly, BCC was delivered alongside other intervention components. Although this reflects the reality of real‐world practice (Hallgren et al., 2018), with MI often delivered alongside other interventions to enhance patient engagement and intervention effects (Miller & Rollnick, 2013), we are unable to comment on the specific contribution of BCC to the effect sizes demonstrated. Defining, coding and examining the relative contribution of non‐BCC intervention components to intervention effects represents an important challenge for future research. Finally, our estimate of adherence to the NIH fidelity recommendations may be an underrepresentation of the strategies actually used by the included studies. The checklist is based on what the authors' report, so it is entirely possible that some strategies were used but not reported (i.e. due to journal word count limits).

### Implications for practice

- BCC interventions delivered in real‐world healthcare settings by existing healthcare providers to adult patients may produce modest, short‐term improvements for a range of health behaviours and outcomes. However, evidence for what is actually delivered within intervention and control conditions is currently lacking. GRADE ratings further attenuate our confidence in the strength of these effects.
- There is no clear evidence that incorporation of the NIH fidelity recommendations increases or decreases the overall short‐term or long‐term benefits of BCC. The short‐term effect of BCC for alcohol use may be attenuated when studies pay greater attention to best practice fidelity recommendations. Further evidence will clarify the nature and direction of this effect.
- There is insufficient evidence to determine the presence, strength and direction of the relationship between provider fidelity to the delivery of BCC and patient health outcomes or behaviours. Further evidence will clarify the importance of fidelity benchmarks and provider responsiveness.

### Implications for research

- Future efforts should focus on clarifying the relative clinical importance of individual NIH fidelity recommendations and domains. Using this evidence to streamline recommended fidelity practices may help to enhance their integration into the design and reporting of future evaluations.
- Assessment and reporting of intervention fidelity need to become a routine component of intervention evaluations. Minimal standards, clear guidance, improved consistency in the definition of intervention components and increased use of standardised methods would allow for easier comparison across studies. To improve the feasibility of incorporating observer‐rated fidelity assessment, trialists should consider examining the reliability and validity of integrating self‐ and peer‐ratings of fidelity into the supervision and feedback process.
- To improve understanding of treatment effects, comprehensive, transparent assessment and reporting of control conditions are needed. Future evaluations should describe, assess and report the dose, delivery and characteristics of *both* the intervention and control condition. Differentiation between intervention and control arms should also be evaluated and reported.
- Future efforts should focus on clarifying the relationship between treatment fidelity and intervention effects. Rigorous experimental designs that allow researchers to manipulate and evaluate the relationship between patient and provider behaviours, intervention delivery and treatment outcome are needed.
- To improve generalisability, further research in developing, low‐ and middle‐income countries; utilising a more diverse range of healthcare providers and settings is needed. Future research should also assess and report the demographic characteristics of intervention providers.

## CONCLUSION

Fidelity is an important methodological consideration when interpreting treatment effects (Bellg et al., 2004; Borrelli, 2011; Borrelli et al., 2015). Understanding how MI interventions perform in real‐world settings represents an important research priority (Hallgren et al., 2018). This is the first systematic review and meta‐analysis to examine the degree to which NIH fidelity recommendations have been incorporated into real‐world evaluations of BCC interventions, and the relationship to treatment outcome. We also provide new evidence for the effect size of BCC when delivered within real‐world healthcare settings by existing providers for a range of health behaviours and outcomes. Together with our narrative synthesis and critical appraisal of fidelity assessment and reporting practices, these findings have important clinical implications and inform key recommendations for future research. This novel, methodologically rigorous systematic review and meta‐analysis addresses an important clinical and research priority, lending new insight into treatment fidelity and related implications for understanding real‐world treatment effects.

## AUTHOR CONTRIBUTIONS

All authors made substantial contributions to conception, design, methods and/or the content of the current publication. Beck contributed to conceptualisation, methodology, formal analysis, investigation, data curation, writing—original draft, writing—review and editing, project administration and funding acquisition; Baker, Britton and Carter contributed to conceptualisation, methodology, writing—review and editing and supervision; Lum, Pohlman, Forbes, Moore, Barnoth and Perkes contributed to methodology, investigation, data curation, validation and writing—review and editing; Oldmeadow contributed to conceptualisation, methodology, supervision and writing—review and editing. All authors offered critical revisions to the manuscript for importantintellectual content, have approved the final version of this manuscript andagree to be accountable for all aspects of the work in ensuring that questionsrelated to the accuracy or integrity of any part of the work are appropriatelyinvestigated and resolved.

## CONFLICT OF INTEREST STATEMENT

None to declare.

## Supporting information


Figure S1

Figure S2

Figure S3

Figure S4

Figure S5

Figure S6

Figure S7

Figure S8

Table S1

Table S2

Table S3

File S1

File S2

File S3

File S4

## Data Availability

This systematic review and meta‐analysis were prospectively registered (17 July 2019) on the International prospective register of systematic reviews: PROSPERO 2019 CRD42019131169. The review protocol has been published and is available from pubmed.ncbi.nlm.nih.gov/31366650/ (doi: 10.1136/bmjopen‐2018‐028417). The data and syntax used in this review is available at the APA's repository on the Open Science Framework and can be accessed at https://osf.io/ac5zd/?view_only=fd03e5bc0e5b4dfe97ddce60fa4269aa.
