# Supplementary material for: Adapted motivational interviewing for brief healthcare consultations: A systematic review and meta‐analysis of treatment fidelity in real‐world evaluations of behaviour change counselling
Source: Br J Health Psychol. 2023 May 4;28(4):972–99. doi: 10.1111/bjhp.12664 (PMC10947272; doi:10.1111/bjhp.12664)
Supplement: Supplementary file 1 — Figure S1 [file BJHP-28-972-s002.docx]

**Supplementary Figure 1**

*NIH Fidelity Checklist Ratings for Each Domain, Grouped According to the Health Behavior(s) Targeted by the BCC Intervention and Overall Fidelity Checklist Score (lowest to highest)*
