# Supplementary material for: Adapted motivational interviewing for brief healthcare consultations: A systematic review and meta‐analysis of treatment fidelity in real‐world evaluations of behaviour change counselling
Source: Br J Health Psychol. 2023 May 4;28(4):972–99. doi: 10.1111/bjhp.12664 (PMC10947272; doi:10.1111/bjhp.12664)

**Supplementary Figure 3**

*Funnel Plot of Observed and Imputed Effects for Meta-analysis of Long-term (>6 months) Patient Outcomes*


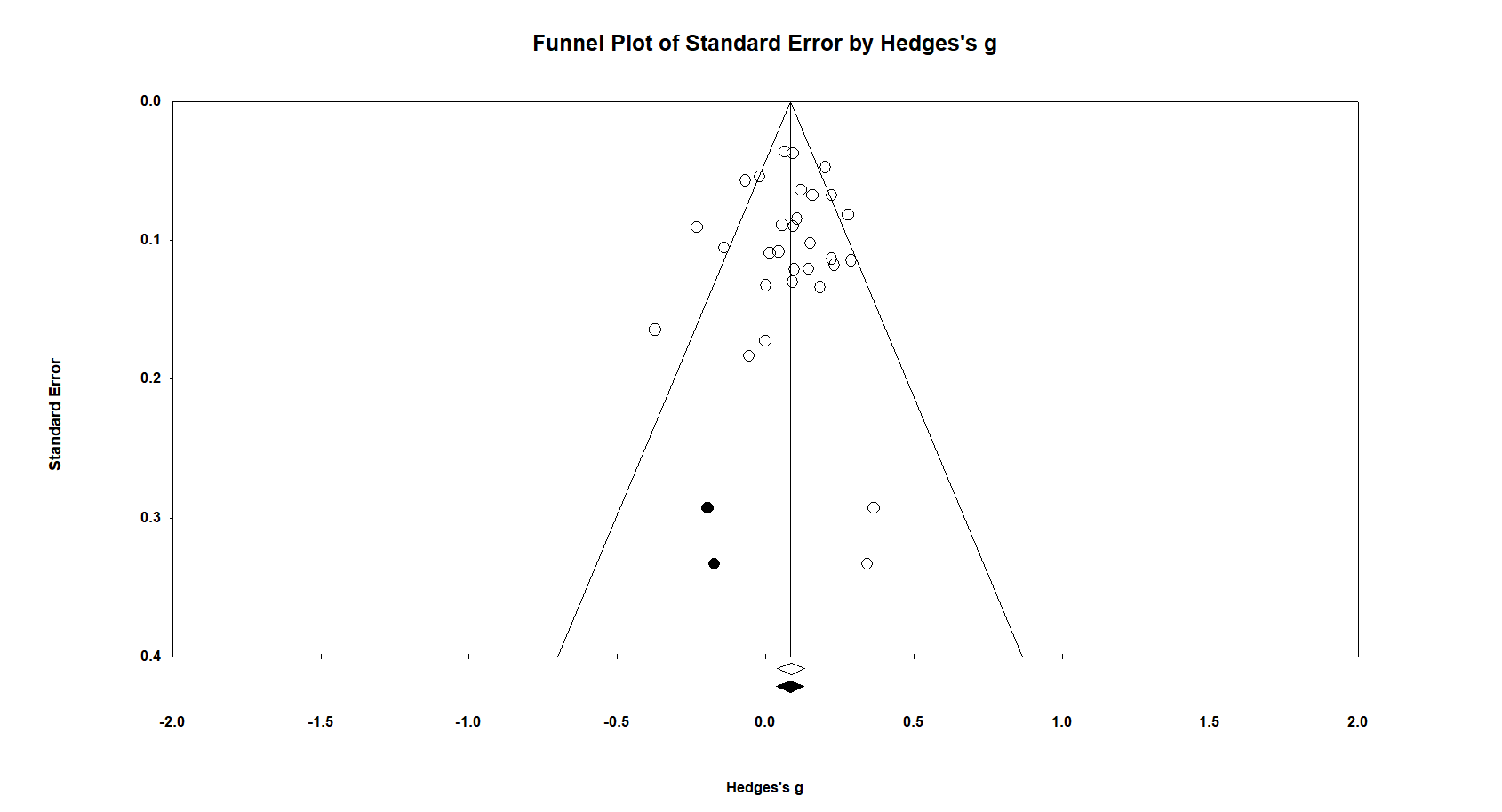

Supplement: Supplementary file 3 — Figure S3 [file BJHP-28-972-s012.docx]
