# Supplementary material for: Adapted motivational interviewing for brief healthcare consultations: A systematic review and meta‐analysis of treatment fidelity in real‐world evaluations of behaviour change counselling
Source: Br J Health Psychol. 2023 May 4;28(4):972–99. doi: 10.1111/bjhp.12664 (PMC10947272; doi:10.1111/bjhp.12664)
Supplement: Supplementary file 4 — Figure S4 [file BJHP-28-972-s004.docx]

**Supplementary Figure 4**

*Meta-regressions Between NIH Checklist Score and Hedges g (Z distribution and Knapp-Hartung Adjustment) for Short-term (n=40) Long-term (n=30); and Short-term Alcohol Sub-group (n=10) Analyses*

|  | *Z* Distribution | Knapp-Hartung Adjustment |
| --- | --- | --- |
| Overall Analysis |  |  |
| Short-term Outcomes | 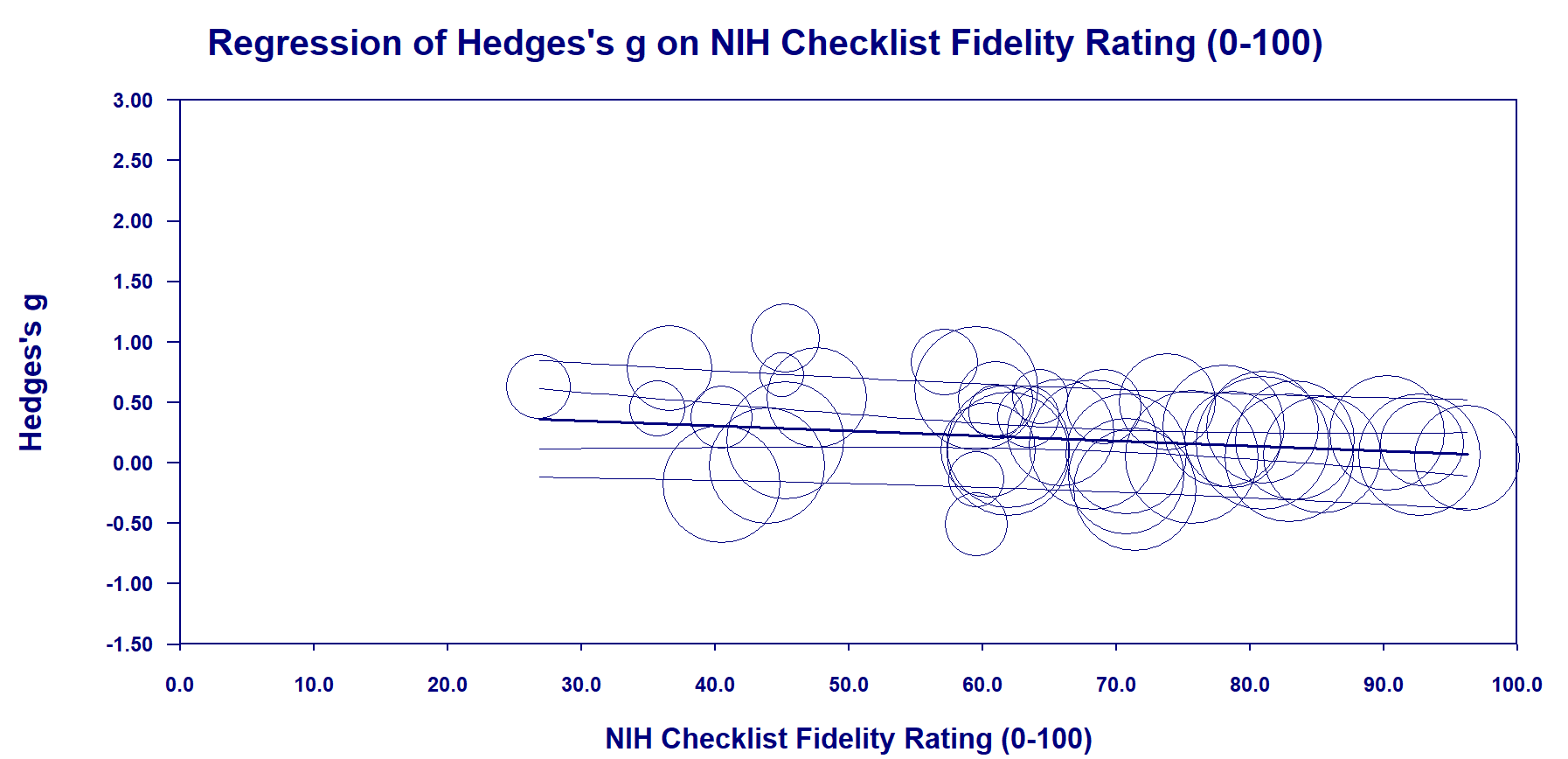 | 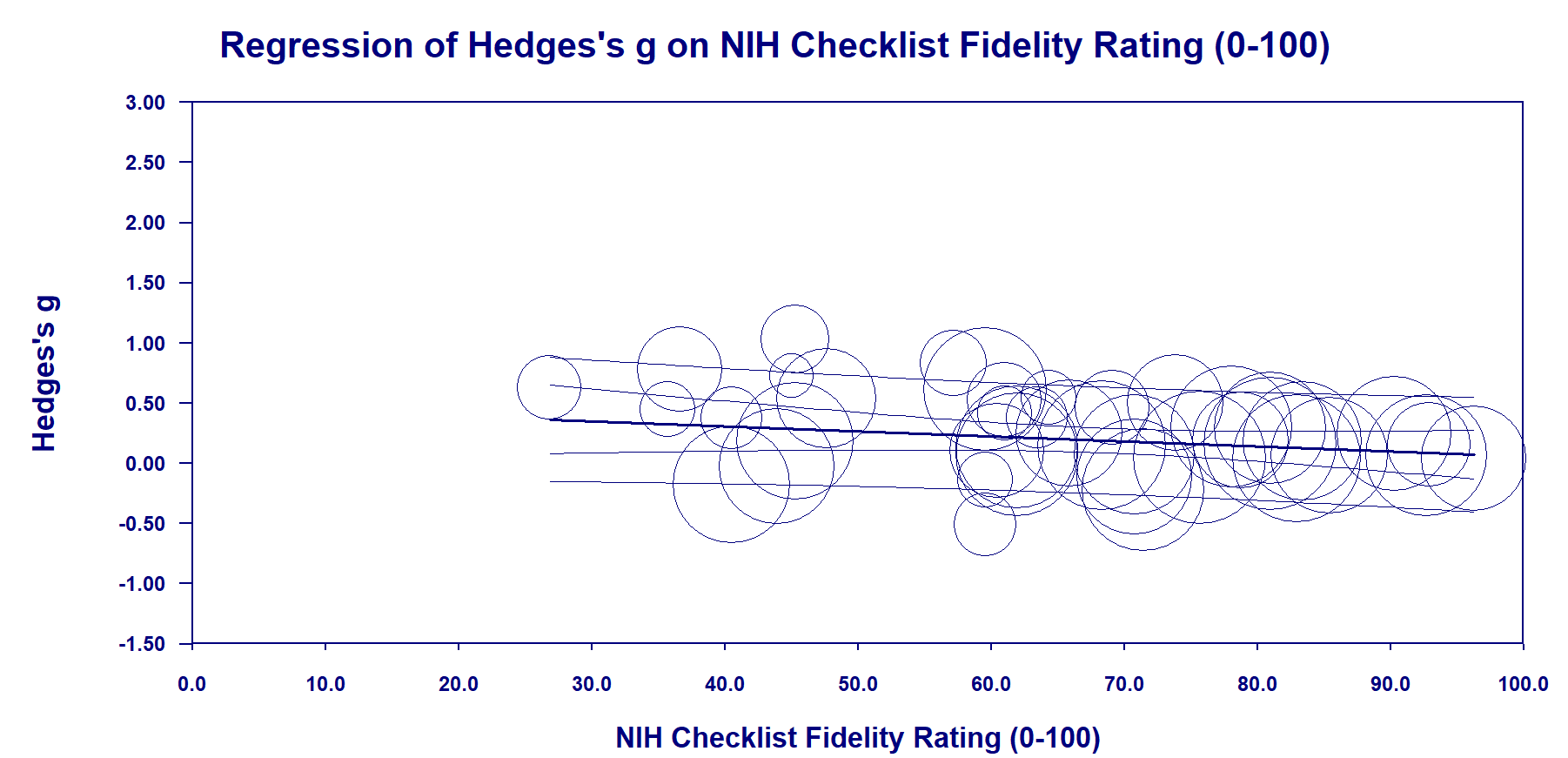 |
| Long-term Outcomes | 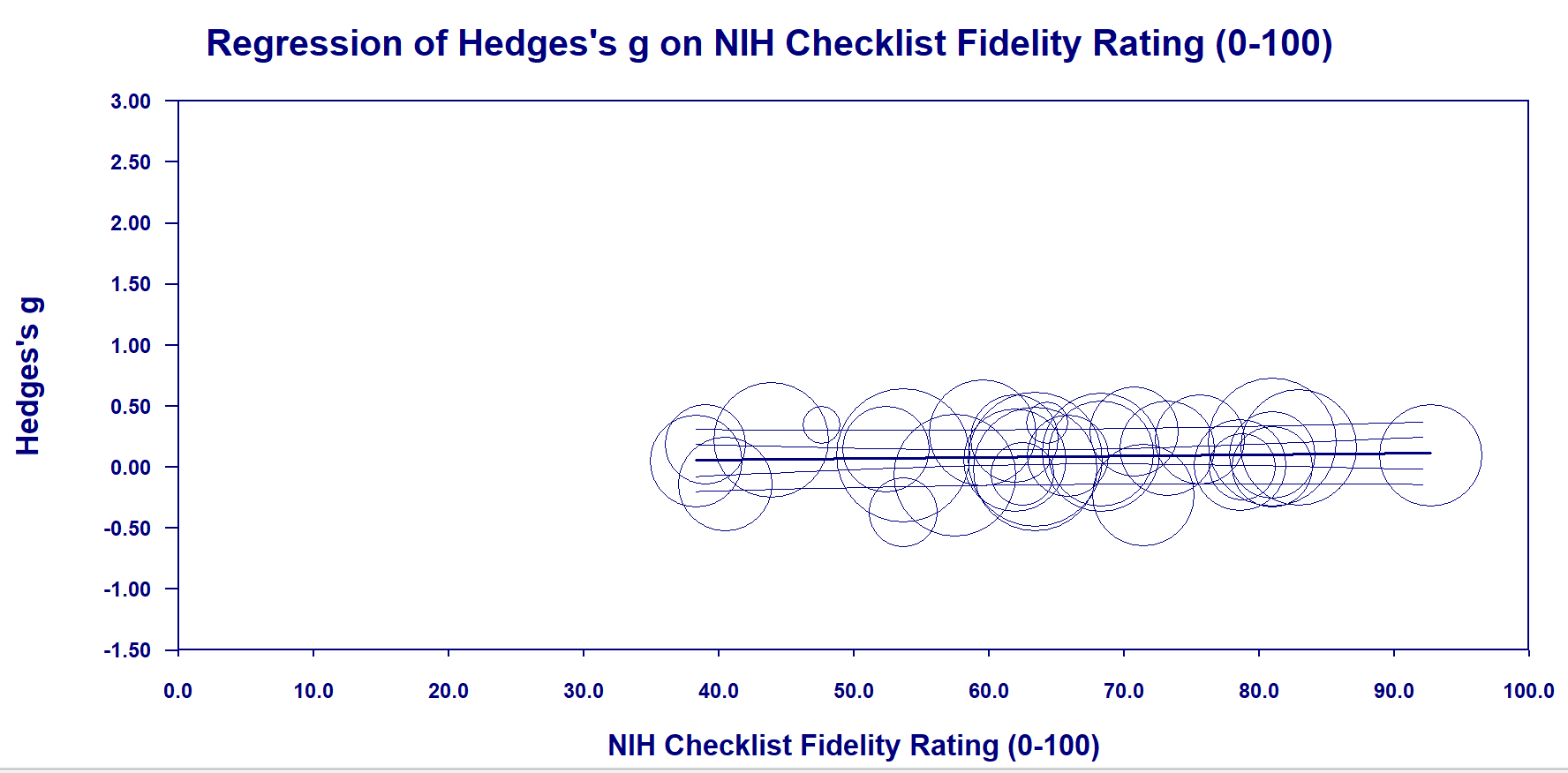 | 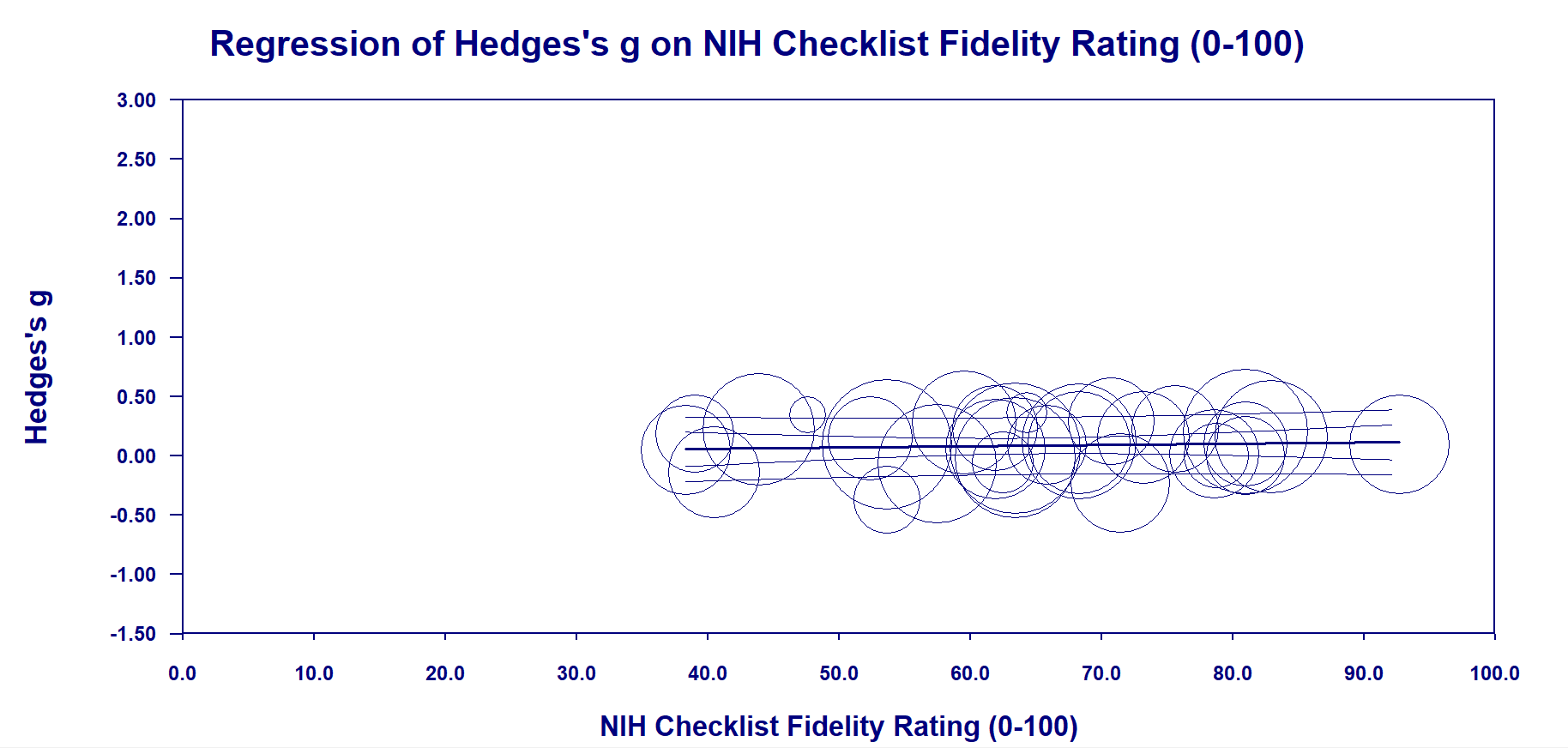 |
| Sub-group Analysis |  |  |
| Short-term Alcohol Outcomes | 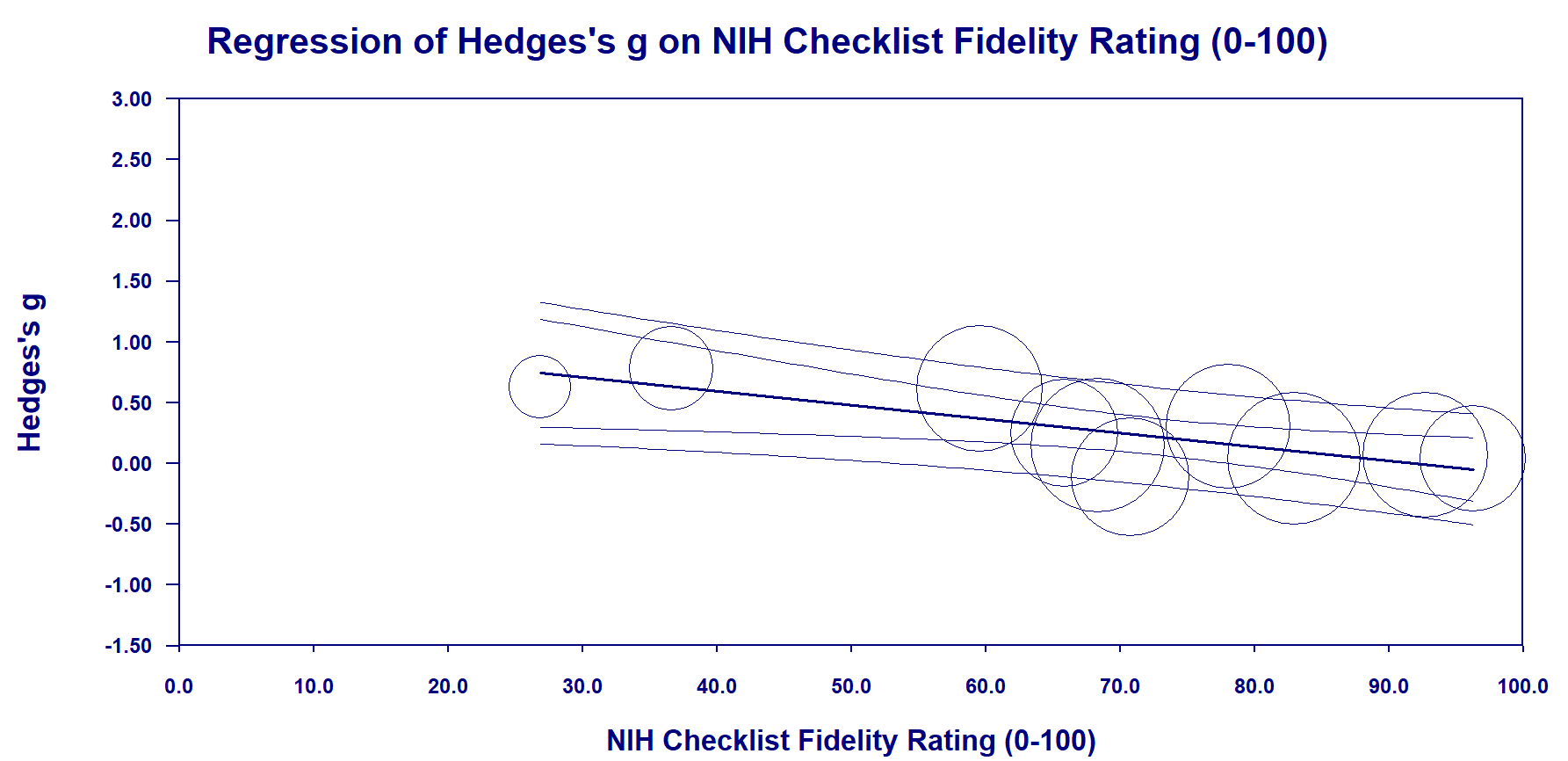 | 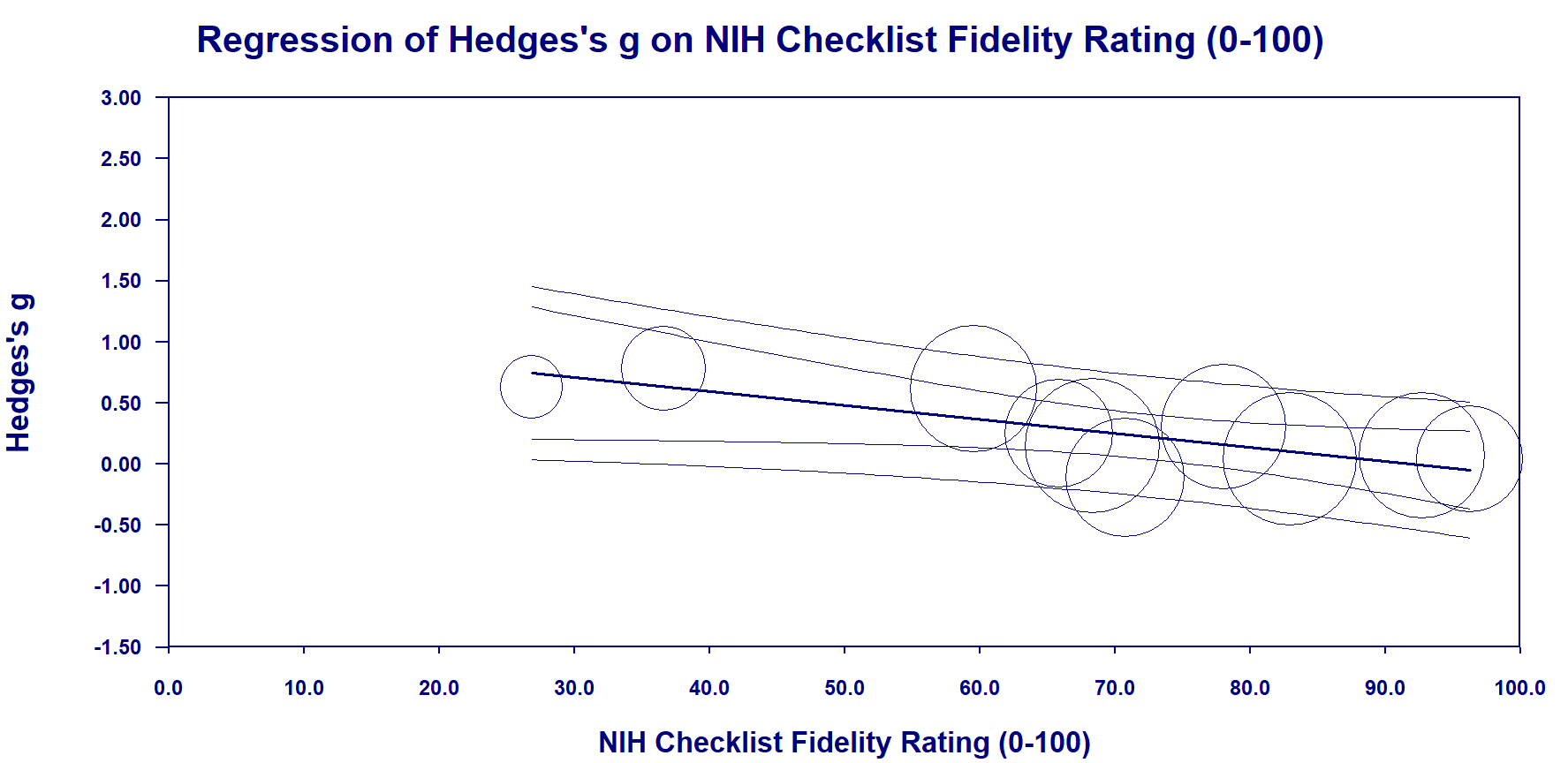 |
