# Supplementary material for: Adapted motivational interviewing for brief healthcare consultations: A systematic review and meta‐analysis of treatment fidelity in real‐world evaluations of behaviour change counselling
Source: Br J Health Psychol. 2023 May 4;28(4):972–99. doi: 10.1111/bjhp.12664 (PMC10947272; doi:10.1111/bjhp.12664)
Supplement: Supplementary file 5 — Figure S5 [file BJHP-28-972-s014.docx]

**Supplementary Figure 5**

*EPHPP Quality Assessment Ratings Within and Across the Outcome Papers (n=61) of the Included Studies*

|  | Selection Bias | Study Design | Confounders | Blinding | Data Collection Methods | Withdrawals & Drop Outs | Overall Quality |
| --- | --- | --- | --- | --- | --- | --- | --- |
| Substance Use |  |  |  |  |  |  |  |
| Mitchesonet et al (2007) | - | ++ | ++ | + | ++ | ++ | Moderate |
| Gryczynski et al (2015) | + | ++ | ++ | + | + | ++ | Strong |
| Schwartz et al (2014) | + | ++ | ++ | + | ++ | ++ | Strong |
| Jaffray et al (2014) | + | ++ | ++ | + | ++ | + | Strong |
| Mertens et al (2014) | + | ++ | - | + | ++ | ++ | Moderate |
| Garner et al (2020) | ++ | ++ | ++ | + | - | - | Weak |
| Darker et al (2016) | ++ | ++ | ++ | + | ++ | + | Strong |
| Physical Activity |  |  |  |  |  |  |  |
| Jackson et al (2007) | + | ++ | ++ | + | ++ | - | Moderate |
| Elley et al (2003) | + | ++ | - | + | + | ++ | Moderate |
| Dennett et al (2018) | + | ++ | ++ | + | ++ | ++ | Strong |
| O’Halloran et al (2016) | - | ++ | ++ | + | ++ | ++ | Moderate |
| van der Weegen et al (2015) | - | ++ | ++ | + | ++ | ++ | Moderate |
| Smoking |  |  |  |  |  |  |  |
| Louwagie et al (2014) | ++ | ++ | ++ | - | ++ | + | Moderate |
| Cabezas et al (2011) | + | ++ | ++ | + | + | - | Moderate |
| Meyer et al (2012) | + | ++ | ++ | - | - | - | Weak |
| Cossette et al (2012) | + | ++ | ++ | + | ++ | - | Moderate |
| Glasgow et al (2000) | + | ++ | ++ | + | + | ++ | Strong |
| Ershoff et al (1999) | ++ | ++ | ++ | + | + | + | Strong |
| Butler et al (1999) | + | ++ | ++ | + | + | + | Strong |
| Borrelli et al (2005) | ++ | ++ | ++ | + | - | + | Moderate |
| Hollis et al (2007) | + | ++ | ++ | + | - | + | Moderate |
| Treatment adherence/ engagement |  |  |  |  |  |  |  |
| Leiva et al (2014) | + | ++ | ++ | + | - | ++ | Moderate |
| Eyler et al (2016) | + | ++ | ++ | + | - | ++ | Moderate |
| Drevenhorn et al (2012) | - | ++ | - | - | - | ++ | Weak |
| Cook et al (2017) | + | ++ | ++ | + | ++ | ++ | Strong |
| Graham et al (2016) | - | ++ | - | + | ++ | ++ | Weak |
| Hedegaard et al (2016) | + | ++ | ++ | + | + | + | Strong |
| George et al (2021) | ++ | ++ | - | ++ | ++ | ++ | Moderate |
| Alcohol |  |  |  |  |  |  |  |
| Bager et al (2010) | + | ++ | ++ | - | - | - | Weak |
| Noknoy et al (2010) | + | ++ | + | ++ | + | ++ | Strong |
| Aalto et al (2000) | - | ++ | + | + | + | - | Weak |
| Aalto et al (2001) | - | ++ | + | + | + | + | Moderate |
| L'Engle et al (2014) | - | ++ | - | + | + | ++ | Weak |
| Schaus et al (2009) | - | ++ | + | + | ++ | - | Weak |
| Fleming et al (2010) | + | ++ | ++ | ++ | ++ | ++ | Strong |
| Dhital et al (2015) | + | ++ | + | + | ++ | + | Strong |
| Ockene et al (1999) | + | ++ | ++ | ++ | ++ | ++ | Strong |
| Zatzick et al (2014) | - | ++ | + | ++ | ++ | + | Moderate |
| D'Onofrio et al (2008) | + | ++ | - | ++ | ++ | ++ | Moderate |
| Shin et al (2013) | + | ++ | ++ | - | + | ++ | Moderate |
| Sub-optimal glycaemic control |  |  |  |  |  |  |  |
| Lauffenburger et al (2019) | - | ++ | ++ | ++ | - | ++ | Weak |
| Juul et al (2014) | ++ | ++ | ++ | + | - | + | Moderate |
| Ismail et al (2018) | + | ++ | - | + | ++ | + | Moderate |
| Multiple health behavior change |  |  |  |  |  |  |  |
| Christian et al (2011) | + | ++ | ++ | + | ++ | ++ | Strong |
| Christian et al (2008) | + | ++ | ++ | - | - | ++ | Weak |
| Lakerveld et al (2013) | + | ++ | ++ | + | ++ | + | Strong |
| Heinrich et al (2010) | - | ++ | - | + | - | - | Weak |
| Whittemore et al (2009) | + | ++ | ++ | + | - | ++ | Strong |
| Verweij et al (2012) | + | ++ | ++ | + | - | ++ | Moderate |
| Koelewijn-van Loon et al (2010) | - | ++ | ++ | + | + | + | Moderate |
| Koelewijn-van Loon et al (2009) | - | ++ | ++ | + | + | ++ | Moderate |
| Nanchahal et al (2012) | + | ++ | ++ | + | - | + | Moderate |
| Butler et al (2013) | + | ++ | ++ | + | ++ | - | Moderate |
| Jansink et al (2013) | - | ++ | - | + | - | - | Weak |
| Bóveda-Fontán et al (2015) | + | ++ | ++ | - | - | ++ | Weak |
| Other |  |  |  |  |  |  |  |
| Godard et al (2011) | + | ++ | ++ | + | - | - | Weak |
| Dermen et al (2014) | - | ++ | ++ | ++ | - | - | Weak |
| Cornman et al (2008) | - | ++ | ++ | + | - | + | Weak |
| Hegarty et al (2013) | ++ | ++ | ++ | - | ++ | + | Moderate |
| Fisher et al (2014) | - | ++ | ++ | + | - | + | Moderate |
| Britton et al (2019) | - | ++ | ++ | + | ++ | ++ | Strong |
| Weak | 19 (31.15%) | 0  (0%) | 10  16.39%) | 8 (13.11%) | 21 (34.43%) | 13 (21.31%) | 16 (26.23%) |
| Moderate | 34  (55.74%) | 0  (0%) | 6  (9.83%) | 45 (73.77%) | 14  (22.95%) | 19 (31.14%) | 27 (44.26%) |
| Strong | 8  (13.11%) | 61  (100%) | 45  (73.77%) | 8 (13.11%) | 26  (42.62%) | 29 (47.54%) | 18 (29.51%) |
