# Supplementary material for: Adapted motivational interviewing for brief healthcare consultations: A systematic review and meta‐analysis of treatment fidelity in real‐world evaluations of behaviour change counselling
Source: Br J Health Psychol. 2023 May 4;28(4):972–99. doi: 10.1111/bjhp.12664 (PMC10947272; doi:10.1111/bjhp.12664)
Supplement: Supplementary file 6 — Figure S6 [file BJHP-28-972-s009.docx]

**Figure 9**

*Risk of Bias Ratings for Included Studies (n=58)*

|  | Bias arising from the randomisation process | | | Bias due to deviations from intended interventions | | Bias due to missing outcome data | | Bias in measurement of the outcome | | Bias in selection of the reported result | | Overall Risk of Bias |  |  |  |
| --- | --- | --- | --- | --- | --- | --- | --- | --- | --- | --- | --- | --- | --- | --- | --- |
| Substance use |  | | |  | |  | |  | |  | |  |  |  |  |
| Mitcheson et al (2007) | | 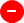 | 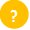 | | 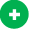 | | 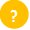 | | 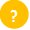 | | 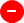 | | 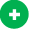 | Low risk |  |
| Gryczynski et al (2014; 2015) | | 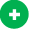 | 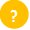 | | 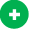 | | 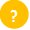 | | 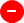 | | 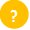 | | 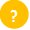 | Some concerns |  |
| Jaffray et al (2014) | | 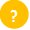 | 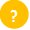 | | 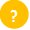 | | 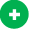 | | 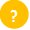 | | 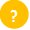 | | 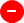 | High risk |  |
| Mertens et al (2014) | | 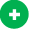 | 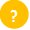 | | 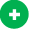 | | 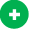 | | 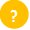 | | 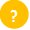 | |  |  |  |
| Garner et al (2020) | | 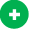 | 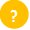 | | 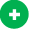 | | 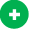 | | 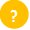 | | 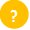 | |  |  |  |
| Darker et al (2016) | | 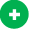 | 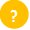 | | 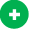 | | 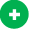 | | 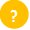 | | 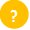 | |  |  |  |
| Physical activity | |  |  | |  | |  | |  | |  | |  |  |  |
| Jackson et al (2007) | | 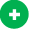 | 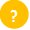 | | 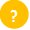 | | 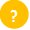 | | 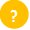 | | 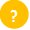 | |  |  |  |
| Elley et al (2003) | | 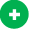 | 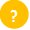 | | 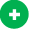 | | 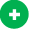 | | 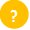 | | 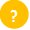 | |  |  |  |
| Dennett et al (2018) | | 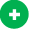 | 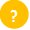 | | 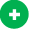 | | 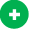 | | 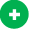 | | 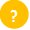 | |  |  |  |
| O’Halloran et al (2016) | | 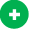 | 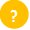 | | 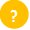 | | 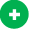 | | 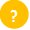 | | 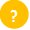 | |  |  |  |
| van der Weegen et al (2015) | | 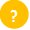 | 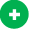 | | 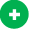 | | 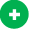 | | 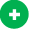 | | 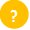 | |  |  |  |
| Smoking | |  |  | |  | |  | |  | |  | |  |  |  |
| Louwagie et al (2014) | | 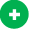 | 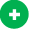 | | 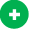 | | 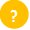 | | 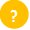 | | 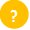 | |  |  |  |
| Cabezas et al (2011) | | 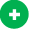 | 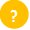 | | 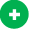 | | 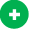 | | 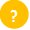 | | 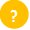 | |  |  |  |
| Meyer et al (2012) | | 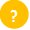 | 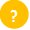 | | 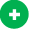 | | 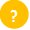 | | 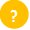 | | 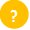 | |  |  |  |
| Cossette et al (2012) | | 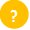 | 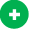 | | 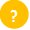 | | 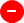 | | 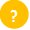 | | 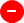 | |  |  |  |
| Glasgow et al (2000) | | 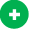 | 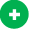 | | 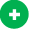 | | 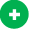 | | 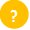 | | 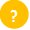 | |  |  |  |
| Ershoff et al (1999) | | 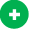 |  | |  | |  | |  | |  | |  |  |  |
| Butler et al (1999) | |  |  | |  | |  | |  | |  | |  |  |  |
| Borrelli et al (2005) | |  |  | |  | |  | |  | |  | |  |  |  |
| Hollis et al (2007) | |  |  | |  | |  | |  | |  | |  |  |  |
| Treatment adherence/ engagement | |  |  | |  | |  | |  | |  | |  |  |  |
| Leiva et al (2014) | |  |  | |  | |  | |  | |  | |  |  |  |
| Eyler et al (2016) | |  |  | |  | |  | |  | |  | |  |  |  |
| Drevenhorn et al (2012) | |  |  | |  | |  | |  | |  | |  |  |  |
| Cook et al (2017) | |  |  | |  | |  | |  | |  | |  |  |  |
| Graham et al (2016) | |  |  | |  | |  | |  | |  | |  |  |  |
| Hedegaard et al (2016) | |  |  | |  | |  | |  | |  | |  |  |  |
| George et al (2021) | |  |  | |  | |  | |  | |  | |  |  |  |
| Alcohol | |  |  | |  | |  | |  | |  | |  |  |  |
| Bager et al (2010) | |  |  | |  | |  | |  | |  | |  |  |  |
| Noknoy et al (2010) | |  |  | |  | |  | |  | |  | |  |  |  |
| Aalto et al (2000; 2001) | |  |  | |  | |  | |  | |  | |  |  |  |
| L'Engle et al (2014) | |  |  | |  | |  | |  | |  | |  |  |  |
| Schaus et al (2009) | |  |  | |  | |  | |  | |  | |  |  |  |
| Fleming et al (2010) | |  |  | |  | |  | |  | |  | |  |  |  |
| Dhital et al (2015) | |  |  | |  | |  | |  | |  | |  |  |  |
| Ockene et al (1999) | |  |  | |  | |  | |  | |  | |  |  |  |
| Zatzick et al (2014) | |  |  | |  | |  | |  | |  | |  |  |  |
| D'Onofrio et al (2008) | |  |  | |  | |  | |  | |  | |  |  |  |
| Shin et al (2013) | |  |  | |  | |  | |  | |  | |  |  |  |
| Sub-optimal glycaemic control | |  |  | |  | |  | |  | |  | |  |  |  |
| Lauffenburger et al (2019) | |  |  | |  | |  | |  | |  | |  |  |  |
| Juul et al (2014) | |  |  | |  | |  | |  | |  | |  |  |  |
| Ismail et al (2018) | |  |  | |  | |  | |  | |  | |  |  |  |
| Multiple health behavior change | |  |  | |  | |  | |  | |  | |  |  |  |
| Christian et al (2011) | |  |  | |  | |  | |  | |  | |  |  |  |
| Christian et al (2008) | |  |  | |  | |  | |  | |  | |  |  |  |
| Lakerveld et al (2013) | |  |  | |  | |  | |  | |  | |  |  |  |
| Heinrich et al (2010) | |  |  | |  | |  | |  | |  | |  |  |  |
| Whittemore et al (2009) | |  |  | |  | |  | |  | |  | |  |  |  |
| Verweij et al (2012) | |  |  | |  | |  | |  | |  | |  |  |  |
| Koelewijn-van Loon et al (2009; 2010) | |  |  | |  | |  | |  | |  | |  |  |  |
| Nanchahal et al (2012) | |  |  | |  | |  | |  | |  | |  |  |  |
| Butler et al (2013) | |  |  | |  | |  | |  | |  | |  |  |  |
| Jansink et al (2013) | |  |  | |  | |  | |  | |  | |  |  |  |
| Bóveda-Fontán et al (2015) | |  |  | |  | |  | |  | |  | |  |  |  |
| Other | |  |  | |  | |  | |  | |  | |  |  |  |
| Godard et al (2011) | |  |  | |  | |  | |  | |  | |  |  |  |
| Dermen et al (2014) | |  |  | |  | |  | |  | |  | |  |  |  |
| Cornman et al (2008) | |  |  | |  | |  | |  | |  | |  |  |  |
| Hegarty et al (2013) | |  |  | |  | |  | |  | |  | |  |  |  |
| Fisher et al (2014) | |  |  | |  | |  | |  | |  | |  |  |  |
| Britton et al (2019) | |  |  | |  | |  | |  | |  | |  |  |  |
| Low | | 36 (62.09%) | 14 (24.13%) | | 44 (75.86%) | | 33 (56.89%) | | 11 (18.97%) | | 5 (8.62%) | |  |  |  |
| Some Concerns | | 20 (34.48%) | 33 (56.89%) | | 11 (18.96%) | | 18 (31.03%) | | 38 (65.52%) | | 38 (65.51%) | |  |  |  |
| High | | 2 (3.44%) | 11 (18.96%) | | 3 (5.17%) | | 7 (12.07%) | | 9 (15.52%) | | 15 (25.86%) | |  |  |  |
