# Supplementary material for: Adapted motivational interviewing for brief healthcare consultations: A systematic review and meta‐analysis of treatment fidelity in real‐world evaluations of behaviour change counselling
Source: Br J Health Psychol. 2023 May 4;28(4):972–99. doi: 10.1111/bjhp.12664 (PMC10947272; doi:10.1111/bjhp.12664)
Supplement: Supplementary file 8 — Figure S8 [file BJHP-28-972-s005.docx]

**Supplementary Figure 8**

*NIH Checklist Score According to Fidelity Domain and Study Year*
