# Supplementary material for: Adapted motivational interviewing for brief healthcare consultations: A systematic review and meta‐analysis of treatment fidelity in real‐world evaluations of behaviour change counselling
Source: Br J Health Psychol. 2023 May 4;28(4):972–99. doi: 10.1111/bjhp.12664 (PMC10947272; doi:10.1111/bjhp.12664)
Supplement: Supplementary file 9 — Table S1 [file BJHP-28-972-s006.docx]

**Table S1**

*Summary of Key Study Characteristics Grouped According to the Health Behaviour(s)^a^ Targeted by the BCC Intervention*

| **Study Details** | | | |  | **Primary Outcome** | |  | **Intervention Arm(s)** | | | |  | **Control Condition** | |  |  |
| --- | --- | --- | --- | --- | --- | --- | --- | --- | --- | --- | --- | --- | --- | --- | --- | --- |
| Author/  Publication Year/  Design | Setting/  Country | Target Population | Target health behaviour(s) |  | Definition/  Assessment  Method | Follow-up interval(s)^b^ |  | Description of BCC Intervention^i^/  Any Additional Treatment Arms | Session details (length, number, frequency) | Duration | Provider type/  Age: M(SD)/  Gender: n(%) female |  | Description | Provider type/  Age: M(SD)/  Gender: n(%) female |  |  |
| **Substance Use** | | | |  |  |  |  |  |  |  |  |  |  |  |  |  |
| Mitcheson (2007)  Cluster RCT | The Methadone Maintenance Clinic  England | Methadone maintenance population | Reduce crack cocaine use |  | Daily amount (rocks; past 30 days)  MAP | 3-months post-intervention^†^ |  | **MI:** Explore/resolve ambivalence; promote reflection on drug use, personal consequences, goals and values, support change | ~30 min x 1 | Single session | Key worker (n=?)^c^  Age: ?  Gender: ? |  | **Crack Information Control Condition:**  Crack awareness’ initiative (reception poster display; in-house information leaflets) | Provider: ? (n=?) ^c^  Age: ?  Gender: ? |  |  |
| Gryczynski (2015)  Parallel, two-arm RCT | Community health centres  USA | Primary care patients with moderate-level illicit drug use | Reduce drug risk and use |  | Global Continuum of Illicit Drug Risk score  ASSIST | 3-, 6-^†^ and  12-^§^  months |  | **In-person BI:** Standard BI and MI techniques (content tailored to motivation and confidence to change), included gender-specific normative feedback (according to clinician preference). | ~14min x 1 | Single session | Behavioural health counsellor (n=2)  Age: ?  Gender: ? |  | **Computer Delivered BI**: Animated avatar asked questions, BI and MI (content tailored to motivation and confidence to change), gender specific normative feedback | NA |  |  |
| Schwartz (2014)  Parallel, two-arm RCT | As above | As above | As above |  | As above | 3-months^d^ |  | **In-person BI:** As above | As above | As above | As above |  | **Computer delivered BI:** As above | NA |  |  |
| Jaffray (2014)  Cluster RCT | Community pharmacies  Scotland | Supervised methadone patients | Reduce heroin use to improve methadone outcomes |  | Illicit heroin use past 30 days (% patients)  MAP | 6-months^†^ |  | **The Enhanced Pharmacy Service Intervention:** MI techniques over a number of visits, building on prior discussions (focused on reducing illicit heroin and other drug use). | <30min x ‘as needed’ | 6-months | Pharmacist (n=?) ^c^  Age: ?  Gender: ? |  | **Normal Practice**: Not described. | Pharmacist (n=?)^c^  Age: ?  Gender: ? |  |  |
| Mertens (2014)  Single-blinded, parallel-group RCT | Public-sector primary health care clinic  South Africa | Young adult primary care patients demonstrating risky alcohol/ drug use | Reduce risky alcohol and drug use and associated harms |  | Alcohol and drug use and problems (past 3 months)  ASSIST | 3-months^†^ |  | **Brief Motivational Intervention:** Based on “Health Behaviour Change: A Guide for Practitioners”, resource list for drinking and drug use problems | ~10min x 1 | Single session | Nurse practitioner (n=?)  Age: ?  Gender: ? |  | **Minimally Enhanced UC:**  UC (not described), resource list for drinking and drug use problems | Provider: ? (n=?)  Age: ?  Gender: ? |  |  |
| Garner (2020)  Cluster RCT | HIV community based organisations  USA | People with HIV | Reduce substance use |  | Days of primary substance use (past 28 days)  Modified version of the ASI | 4-weeks^†^ |  | **MIBI + ISF + ATTC + UC:** MIBI: BI based on MI (reasons for change, feedback, importance/ confidence, change plan)  Implementation strategies:  *ISF:* monthly external facilitation (max 30hrs, virtual and 2 x in-person) x 18-months.  *ATTC:* 12 months of training and technical assistance (max 30hrs: online didactics, 2-day in-person workshop; ongoing feedback, monthly group-consultations, < 3 individual consultations)  UC: referral to formal addiction treatment, mutual-help services, or both. | 20-30min x 1 | Single session | Case manager (n=?) ^c^  Age: ? ^c^  Gender: ? ^c^ |  | **ATTC + ISF + UC**  No MIBI, both implementation strategies, UC | Case manager (n=?) ^c^  Age: ? ^c^  Gender: ? ^c^ |  |  |
|  |  |  |  |  |  |  |  | **MIBI + ATTC + UC**  MIBI, ATTC only, UC | 20-30min x 1 | Single session | Case manager (n=?)^c^  Age: ?^c^  Gender: ?^c^ |  | **ATTC+UC**  No MIBI, ATTC only, UC | Case manager (n=?)^c^  Age: ?^c^  Gender: ?^c^ |  |  |
| Darker (2016)  Cluster RCT | Addiction treatment clinics  Ireland | Patients attending methadone maintenance clinics | Reduce problem alcohol use and illicit substance use |  | Global substance use risk score (excluding tobacco)  ASSIST | 3-months^†^ |  | **BI:** BI (based on MI, SOC and FRAMES) guided by ASSIST feedback report | ~16min x 1 | Single session | Clinical staff (n=?)^c^  Age: ?  Gender: ? |  | **TAU Control:** Pharmacist dispensed methadone, doctors appointments (depending on patient needs and stability), access to counselling (depending on availability and participant preference). | Provider: ? (n=?) ^c^  Age: ?  Gender: ? |  |  |
| **Physical Activity** | | | |  |  |  |  |  |  |  |  |  |  |  |  |  |
| Jackson (2007)  RCT | Diabetes outpatient clinic  UK | Adults not meeting recommended guidelines for PA who attended a GP appointment | Increase PA and maintain changes in exercise behaviour |  | Self-reported PA levels (minutes)  PAQ | 6-weeks^†^ |  | **Exercise Consultation Interview:** TTM stage specific behaviour change intervention (MI +/- change plan/ relapse prevention), “Keeping Fit” (standard PA leaflet). | 20-30min x 1 | Single session | Dietitian (n=1)  Age: ?  Gender: ? |  | **Control:** “Keeping Fit”: (standard PA leaflet). | Provider: ? (n=?)  Age: ?  Gender: ? |  |  |
| Elley (2003)  Cluster RCT | General practices  New Zealand | Adults not meeting recommended guidelines for PA who attended a GP appointment | Increase PA (change in total expenditure of energy) |  | Total energy expenditure (past 3 months)  Self-administered questionnaire from the Auckland heart study | 12-months^§^ |  | **The "Green Prescription" Intervention:** Stage of change assessment, exercise prescription, MI strategies (to encourage and support progress towards exercise goals), newsletters | 10-20min x 3 (minimum) | 3-months | GP (n=?); practice nurse (n=?); exercise specialist (n=?)  Age: ?  Female GP: n=17 (28%) |  | **Control:** Care as usual (not described) | GP (n=?)  Age: ?  Female GP: n=18 (31%) |  |  |
| Dennett (2018)  Single-blind, parallel RCT | Outpatient oncology rehabilitation  Australia | Oncology patients | Increase adherence to recommended PA levels |  | Amount of PA of at least moderate intensity completed in 10-minute bouts (past 1-week)  Tri-axial accelerometer | 8-weeks^†^ |  | **MI Intervention:** Telephone MI (engagement, focusing, evoking, planning per participant progress) + standard oncology rehabilitation | 20-30min x 7 weekly calls | 7-weeks | Physiotherapist (n=1)  Age: ?  Gender: ? |  | **Standard Practice**: Oncology rehabilitation (weekly group education/ exercise sessions + tailored home exercise programme and diary to monitor adherence) | Physiotherapist (n=1); nurse, allied health assistant; nurse, social worker, occupational therapist, dietitian  Age: ?  Gender: ? |  |  |
| O’Halloran (2016)  Single-blind RCT | Community rehabilitation programme.  Australia | People living in the community after hip fracture | Increase PA |  | Steps taken per day (past 7 days)  Accelerometer (ActivPal, PAL Technologies, Glasgow) | 9-weeks^†^ |  | **MI:** Telephone-based MI intervention (eliciting change talk; building importance/ confidence in change; address ambivalence) | 30min x 8 (weekly) | 2-months | Physiotherapist (n=1)  Age: ?  Gender: ? |  | **UC:** GP/ physiotherapist 'as needed' | Provider: ? (n=?)  Age: ?  Gender: ? |  |  |
| Van der Weegen (2015)  Cluster RCT | GP practices  The Netherlands | 40-70 year-old patients with COPD or T2D | Stimulate PA |  | PA, past 5-8 days (average minutes/ day)  Personal Activity Monitor AM300(Pam) | 4-6-^†^ and 9-^§^  months |  | **The Self-Management Support Program**: Lifestyle coaching based on “Five ‘A’s Cycle” counseling technique | 20min x 4 (weeks 1 and 2; 2-3 months; 4-6 months) | 4-6-months | Practice nurse (n=?)  Age: ?  Gender: ? |  | **Care as Usual:** Regular consultations (not described) | Provider: ? (n=?)  Age: ?  Gender: ? |  |  |
|  |  |  |  |  |  |  |  | **Complete 'It's Life' Intervention:** “It's Life” monitoring and feedback tool (activity monitor, mobile phone app, Web app) + self-management support program. | 20min x 4 (weeks 1 and 2; 2-3 months; 4-6 months) + 24/7 access to tool | 4-6-months | Practice nurse (n=?)  Age: ?  Gender: ? |  |  |  |  |  |
| **Smoking** | | | |  |  |  |  |  |  |  |  |  |  |  |  |  |
| Louwagie (2014)  Multi-centre, two-group, parallel, individual RCT | Primary care TB clinics  South Africa | Newly diagnosed TB patients identified as current smokers | Smoking cessation |  | Sustained abstinence (6 months)  Interviewer administered question | 1-, 3- and 6-^† e^ months |  | **Brief MI:** Assessment, patient identified problems, solutions and setting of targets; referral for TB treatment, standardised smoking cessation message, smoking cessation booklet | 15-20min x 1 | Single session | Lay health care workers (n=8)  Age: ?  Gender: ? |  | **Brief Smoking Cessation Advice:** TB diagnosis and treatment, HIV counselling and testing, referral to antiretroviral treatment services, standardised smoking cessation message, smoking cessation booklet | TB nurse (n=?)  Age: ?  Gender: ? |  |  |
| Cabezas (2011)  Cluster RCT | Primary care centres  Spain | Smokers attending a primary care appointment | Sustained smoking abstinence |  | Continuous abstinence (1 month)  Telephone interview | 6-, 12- and  24-^§ e^ months |  | **Stepped Smoking Cessation Intervention**: Evidence-based clinical practice guideline: Brief MI, brief advice or intensive intervention + pharmacotherapy according to stage of change | ~20-30min x 1-10 (per stage of change and participant preference) | 6-months | Family physician and nurse (n=?)  Age: ?  Gender: ? |  | **Standard Care:** Brief smoking cessation advice for diseases related to tobacco consumption | Family physician and nurse (n=?)  Age: ?  Gender: ? |  |  |
| Meyer (2012)  Cluster RCT | General practice  Germany | Smokers attending a general practice appointment | Smoking cessation |  | Point prevalence abstinence  ‘Not even a puff’ | 12-months^§^ |  | **Brief Advice:** 'Health BCC' and 'communication strategies' to deliver tailored brief advice (according to cigarettes per day, nicotine dependence and stage of change), self-help manual (tailored to participant stage of change) | ~10min x 3 (maximum) | 6-months | GP (n=58)  *M*=49.8 (*SD*=8.8) years old  Female: n=40 (69%) |  | **Individually Tailored Computer-generated Letters**: Up to 3 letters derived from participant questionnaire scores/ progress, self-help manual (tailored according to participant stage of change) | GP (n=55)  *M*=48.7(*SD*=8.9) years old  Female: n=37 (67.3%) |  |  |
|  |  |  |  |  |  |  |  | **Combination**  'Brief advice' + letter conditions | ~10min x 3 (maximum) + 3 x letters |  | GP (n=56)  *M*=48.9 (*SD*=9.4) years old  Female: n=34 (60.7%) |  |  |  |  |  |
| Cossette (2012)  Pilot RCT | Adult acute-care cardiovascular centre  Canada | Smokers who attended a primary care appointment | Decrease smoking rates |  | Point-prevalence smoking status  Telephone interview | 6-months^†^ |  | **Smoking Cessation Intervention:** MI, information, goal setting, relapse prevention and/ or pharmacotherapy according to stage of change | ~7-12min x 6 phone calls  (4 x weekly, 2 x monthly) | 3-months | Smoking Cessation nurse specialist (n=?)  Age: ?  Gender: ? |  | **UC:** Usual in-hospital smoking cessation support (based on stage of change and MI), referral to community smoking cessation programs | Smoking cessation nurse specialist (n=?)  Age: ?  Gender: ? |  |  |
| Glasgow (2000)  RCT | Planned parenthood clinics  USA | Female smokers | Smoking cessation |  | Smoking cessation (7-days)  Telephone interview | 6-weeks^†^ and 6-months^§^ post-intervention |  | **MI Based Brief Smoking Intervention:** BI based on MI and barrier-based counselling (smoking cessation video, reactions to video, readiness to quit, personalised strategies based on readiness) | 9min video, 12-15min clinician intervention, support calls (as needed) | 1-month | Planned parenthood staff/ 'clinician' (n=?)  Age: ?  Gender: ? |  | **Advice Only:** “Smart Moves”: Generic stop smoking brochure and standardised 20 sec clinician stop smoking advice | Planned parenthood staff/ 'clinician' (n=?)  Age: ?  Gender: ? |  |  |
| Ershoff (1999)  RCT | Large-group-model managed care organisation  USA | Pregnant smokers | Smoking cessation |  | Biochemically confirmed abstinence  Routine urine cotinine | 32-34 week of pregnancy^§^ |  | **MI:** Semi-structured telephone MI (stage of change, confidence,  pros and cons of smoking, temptations and high risk  situations, mutually-agreed-upon goals), self-help booklet | 10-15min x 4-6 (per individual needs) | ? | Preterm nurse educators (n=17)  Age: ?  Gender: ? |  | **Low Cost Self-help Booklet:** “Living Smoke-free”: Tailored to  smoking patterns, stage of change, and lifestyle of pregnant smokers | Unclear who administered the booklet  Age: ?  Gender: ? |  |  |
| Butler (1999)  RCT | General practice  Wales | Smokers attending a GP appointment | Quit or reduce smoking |  | Point prevalence abstinence  Self-report questionnaire | 6-months^†^ |  | **Motivational Consulting:** 3-phase simplified adaptation of MI (rate motivation/ confidence; MI to build motivation/ confidence; patient sets meaningful targets) | ~10min x 1 | Single session | General practice registrars (n=24)  Age: ?^c^  Gender: ?^c^ |  | **Standardised Brief Advice**: Standardised 2-min smoking cessation script | General practice registrars (n=21)  Age: ?^c^  Gender: ?^c^ |  |  |
|  |  |  |  |  |  |  |  |  |  |  |  |  |  |  |  |  |
|  |  |  |  |  |  |  |  | **Interactive Voice Response Telephone Programme:** Computerized telephone cessation program based on interactive voice response  technology, self-help booklet | ~5min as needed (available 24/7 for participants to call) | ? | Health educator (n=?) + interactive voice recording  Age: ?  Gender: ? |  |  |  |  |  |
| Borrelli (2005)  Cluster RCT ^g^ | Home health care: Visiting Nurse Assoc. of Rhode Island  USA | Medically ill patients who smoke | Smoking cessation |  | Continuous abstinence (% ‘yes’)  Self-reported 'refrained from any smoking' | End of treatment, 2-,^†^ 6- and 12-^§^  months post-treatment |  | **Motivational Enhancement:** MI principles + CBT strategies (for enhancing mood and social support), self-help quit smoking manual (‘‘Clear Horizons’’) | 20-30min x 4  (3 + 1 phone call) | ? | Home care nurses (n=46)  Age: ?^c^  Gender: ? |  | **Standard Care:** Based on Agency for Health Care Policy and Research guidelines, self-help quit smoking manual (‘‘Clear Horizons’’) | Home care nurses (n=52)  Age: ? ^c^  Gender: ? |  |  |
| Hollis (2007)  Randomise parallel group, 6 x treatment cells (263 factorial design) | Oregon tobacco Quitline  USA | Adults who planned to quit within the next month (or had quit within the last 7 days) | Smoking cessation |  | Abstinence (> 30 days)  Interviewer administered questionnaire | 6- and 12-months^f^ |  | **Brief Counselling:** Based on BNI (communicate caring, motivate change, information r.e. cessation strategies, anticipate challenges, review previous quit attempts) pharmacotherapy information, referral information, ‘‘quit kit’’ | 15min x 1 | Single session | Quitline tobacco counsellors (n=?)  Age: ?  Gender: ? |  | **---** | --- |  |  |
|  |  |  |  |  |  |  |  | **Brief Counselling + NRT:** As above + free NRT assessment and prescription per FDA guidelines | As above + NRT x 8 weeks | As above + 8-weeks NRT | As above |  |  |  |  |  |
|  |  |  |  |  |  |  |  | **Moderate Counselling:** Based on MI, + follow-up call (support quit plan, encourage use of self-management and community resources), pharmacotherapy information, referral information, ‘‘quit kit’’ | 40min x 1 + 'brief' f/up call (1-2 weeks later) | 2-3 weeks | Quitline tobacco counsellors (n=?)  Age: ?  Gender: ? |  |  |  |  |  |
|  |  |  |  |  |  |  |  | **Moderate Counselling + NRT:** As above + free NRT assessment and prescription per FDA guidelines | As above + NRT x 8 weeks | As above + 8-weeks NRT | As above |  |  |  |  |  |
|  |  |  |  |  |  |  |  | **Intensive Counselling:** MI, stage of change and relapse prevention (as needed), pharmacotherapy information, referral information, ‘‘quit kit’’ | 30-40min x 1;  + 4 (max) support calls | 3-months | Quitline tobacco counsellors (n=?)  Age: ?  Gender: ? |  |  |  |  |  |
|  |  |  |  |  |  |  |  |  |  |  |  |  |  |  |  |  |
|  |  |  |  |  |  |  |  | **Intensive counselling + NRT:** As above + free NRT assessment and prescription per FDA guidelines | As above + NRT x 8 weeks | As above + 8-weeks NRT | As above |  |  |  |  |  |
| **Treatment Adherence/ Engagement** | | | |  |  |  |  |  |  |  |  |  |  |  |  |  |
| Leiva (2014)  Two arm, parallel, multicenter, RCT | Primary care  Spain | Patients with elevated BP | Improve medication adherence (to reduce BP) |  | Systolic BP  According to ESH/ESC guidelines using an OMROM 705 CP automated sphygmomanometer (mean of three measurements) | 12-months^§^ |  | **Multifactorial Adherence-based Intervention:** MI (explore non-adherence to anti-hypertensive treatment, resolve medication related ambivalence, explore locus of control), pillbox reminder, family support, BP self-monitoring, dosing simplification | 25min x 3  (1, 3, 9 months) | 9-months | Nurse (n=?)  Age: ?  Gender: ? |  | **UC:** No change to UC  (not described) | Nurse (n=?)  Age: ?  Gender: ? |  |  |
| Eyler (2016)  Prospective RCT | Tertiary care academic medical centre  USA | Older adults diagnosed with pneumonia | Improve antibiotic adherence |  | Antibiotic prescription fill rates  Interviewer administered question | 24-48 hours post-discharge^†^ |  | **MI Intervention:** MI-enhanced discharge care (elicit patient understanding, assess barriers, assess readiness), pharmacist counselling r.e. antibiotics | ~10min x 1 | Single session | Clinical pharmacists (n=2)  Age: ?  Gender: ? |  | **Standard Care:** Nurse review of discharge instructions | Nurse (n=?)  Age: ?  Gender: ? |  |  |
| Drevenhorn (2012)  RCT | Primary care  Sweden | Patients with hypertension | Improve adherence to the treatment of hypertension (to improve BP and cholesterol levels) |  | Systolic BP  Assessment method: ? | 12- and 24-^§^  months |  | **Multifactorial Tailored Behavioural Counselling:** Counselling informed by patient centredness, the stage of change model, MI and guidelines for CVD prevention, lifestyle factors and pharmacological treatment. | 18-20min x ? (integrated into UC) | ? | Nurse (n=19)  *M*=48 (*SD*=?) years old  Female: n=19 (100%) |  | **UC:** Not described | Nurse (n=14)  *M*=51 (*SD*=?) years old  Gender: ? |  |  |
| Cook (2017)  RCT | Specialty glaucoma clinics  USA | Patients with glaucoma or ocular hypertension | Improve medication adherence |  | Medication adherence (% Doses taken on time)  Medication Event Monitoring System (records date/ time medication opened) | 3-months^†^ |  | **MI:** MI strategies (acknowledging ambivalence, guiding rather than directing, communication skills to elicit patient motivators, challenges and decisions), patient review (medication, treatment, readiness for change) | ~15min x 3 face-to-face (1-, 4-, 8-week), ~7min x 3 phone-call (2-, 6-, 12-week) post-randomisation | 3-months | Ophthalmic technician (n=5)  Age: ?  Gender: ? |  | **UC:** Routine outpatient care by a glaucoma-subspecialty-trained ophthalmologist, written education materials approved by the American Glaucoma Society and/or ad hoc education and support | Glaucoma-subspecialty-trained ophthalmologist (n=?)  Age: ?  Gender: ? |  |  |
|  |  |  |  |  |  |  |  | **Reminder Calls (Minimal Behavioural Intervention):** Scripted telephone calls (adherence, reasons for any missed doses, use of the MEMS bottle, seek info from opthamologist as needed) | ~3min x 3 | 3-months | Age: ?  Gender: ? |  |  |  |  |  |
| Graham (2016)  RCT | In-patient: acute wards and psychiatric intensive care units  England | Mental health in-patients demonstrating alcohol and/ or drug misuse | Improve engagement in treatment for drug and alcohol misuse |  | Treatment engagement  SATS | 3-months^†^ |  | **Brief Integrated MI:** 3-step manual guided, informed by Cognitive-Behavioural Integrated Treatment, strategies from cognitive therapy for substance use and motivational approaches | 15-30min x 4-6 over 2-weeks + 1-month booster | ~6-weeks | In-patient unit staff (n=27 trained, n=12 delivered)  specialist ‘dual diagnosis’ clinicians (n=6 trained, n=5 delivered)  Age: ?  Gender: ? |  | **TAU:** Informed by inpatient policies (including mental state assessment and monitoring, medication, mental state stabilisation) | In-patient nursing and medical staff (n=?)  Age: ?  Gender: ? |  |  |
| Hedegaard (2015)  RCT | Hospital outpatient clinics  Denmark | Patients with hypertension | Improve medication adherence |  | Composite medication possession ratio to antihypertensive and lipid-lowering agents  Prescriptions redeemed data obtained from the Odense University | 9- and 12-^§ e^  months |  | **Multifaceted Pharmacist Intervention:** Guided by a medication adherence questionnaire, adapted DRug Adherence Work-up (DRAW) tool, comprising collaborative care, medication review and adherence counselling based on principles of MI | 10min review x 1; 30min interview x 1; 15min telephone x 2 + additional calls (as needed) | 6-months | Physicians, nurses, clinical pharmacist (n=5)  Age: ?  Gender: ? |  | **UC:** 2-4 outpatient consultations to address risk factors, incl. BP, blood glucose, lipid profiles, medication | Provider: ? (n=?)  Age: ?  Gender: ? |  |  |
| George (2020)    Cluster RCT | Federally qualified health centres (Primary care)  USA | Patients with uncontrolled asthma | Improve asthma control |  | Asthma Control  ACQ | 1-, 2- and 3-^†^ months |  | **BRief Evaluation of AsthmaTHErapy (BREATHE) Intervention:** Semi-scripted, shared decision making intervention based on the BNI, using MI to address erroneous beliefs and non-adherence | 7min x 1 | Single session | Physicians (n=2); nurse practitioners (n=2)  Age: ?^c^  Gender: ?^c^ |  | **Control:** 7‐min unscripted healthy lifestyle (e.g., diet, exercise) discussion | Physicians (n=2); nurse practitioners (n=2)  Age: ?^c^  Gender: ?^c^ |  |  |
| **Alcohol** | | | |  |  |  |  |  |  |  |  |  |  |  |  |  |
| Bager (2010)  RCT | University hospital  Denmark | Discharged patients who drank daily prior to admission | Increase rates of post-discharge alcohol abstinence |  | Post-discharge abstinence rates  Interviewer Administered Questionnaire (similar to ASI) | 2-months post-discharge^†^ |  | **BI:** ‘Bottom-up' (tailored) MI/ BI and referral for 'alcoholism' treatment | 10-15min x 'repeated contacts' (baseline, 2-4 weeks, 2 months minimum) | 2-months | Nurse (n=?), social worker (n=?)  Age: ?  Gender: ? |  | **Control:** Discharged per current practice: Encouraged to contact primary health care service cost-free Public Centers for Alcohol Abuse Therapy | Nurse (n=?), social worker (n=?)  Age: ?  Gender: ? |  |  |
| Noknoy (2010)  RCT | Primary care units  Thailand | Hazardous drinkers | Change drinking behaviour |  | Alcohol consumption past 1 week (drinks per drinking day)  Self-report Standardised Health Survey questionnaire | 6-week, 3- and 6-^†^ months |  | **Brief MET:** Adapted from Project MATCH MI intervention (stage of change assessment, patient centred counseling, feedback, relapse-prevention and MI strategies), delivered per individual stage of change | ~15min x 3  (day 1, 2 and 6 weeks post baseline) | 6-weeks | Nurse (n=?)  Age: ?  Gender: ? |  | **Assessment Only Control Group:** Research assessments only | --- |  |  |
| Aalto (2000)  RCT | Primary and occupational health care clinics  Finland | Female heavy drinkers | Reduce 'heavy drinking' |  | Alcohol amount per week over the past 2 months (grams)  Self-report questionnaire | 12-, 24- and 36-^§ e^ months |  | **BI group A:** FRAMES ingredients per individual patient needs (e.g. adverse effects of alcohol, comparison to recommended guidelines, lab feedback, info r.e. benefits of change, encouragement to reduce drinking) + self-help booklet | 10-20min x 7 (baseline, 2-, 6-, 12-, 18-, 24-, 30- months) | 30-months | GP (n=?)  Age: ?  Gender: ? |  | **Control:** Advice (risks), screening, 2 x GP visits, feedback from lab tests | GP (n=?), nurse (n=?)  Age: ?  Gender: ? |  |  |
|  |  |  |  |  |  |  |  | **BI group B:** FRAMES ingredients per individual patient needs (e.g. adverse effects of alcohol, comparison to recommended guidelines, lab feedback, info r.e. benefits of change, encouragement to reduce drinking) + self-help booklet | 10-20min x 3 (baseline, 12-, 24- months) | 24-months |  |  |  |  |  |  |
| Aalto (2001)  As above | As above | Male heavy drinkers | As above |  | As above | As above |  | **BI group A**  As above | As above | As above | As above |  | **Control**  As above | As above |  |  |
|  |  |  |  |  |  |  |  | **BI group B**  As above | As above | As above |  |  |  |  |  |  |
| L'Engle (2014)  RCT | Drop in centres  Kenya | Moderate drinking female sex workers | Reduce alcohol use and sexually transmitted infection |  | Alcohol use (% Never, < 1/ week, at least 1/ week, daily)  Behavioural Interview (past 30 days) | 6^†^ and 12-^§^  months |  | **BI:** Based on the WHO BI for Alcohol Use (MI techniques, goal-setting, feedback, counseling based on stage of change) | ~20min x 6 across (~monthly) | 6-months | Nurse counsellor (n=?)  Age: ?  Gender: ? |  | **Nutrition Control Intervention:** Based on Kenyan National Guidelines on Nutrition and HIV/AIDS | Nurse counsellor (n=?)  Age: ?  Gender: ? |  |  |
| Schaus (2009)  RCT | University health services (public university)  USA | College students | Reduce high risk drinking |  | Blood alcohol concentration (typical)  Estimated from TLFB (past 30 days) | 3-, 6-,^†^ 9-, and 12-^§^ months |  | **Brief Motivational Interview:** Patient-centred MI techniques and CBT skills; alcohol-prevention educational brochure + feedback | 20min x 2 (fortnightly) | 4-weeks | Physician (n=2); physician assistant (n=1); nurse practitioner (n=1)  Age: ?  Gender: ? |  | **Control:** Alcohol-prevention educational brochure, university health services per usual providers | Provider: ? (n=?)  Age: ?  Gender: ? |  |  |
| Fleming (2010)  RCT | Primary care based college health clinics  USA and Canada | Heavy drinking college students | Reduce alcohol use and related harm |  | Average number of standard drinks (past 28 days)  TLFB | 6^†^ and 12-^§^  months |  | **BI:** Menu of 24 behaviour change intervention strategies (e.g. feedback, information, blood alcohol level calculator, written agreement), booklet | 15min x 2 (monthly) | 2-months | Physician (n=13); nurse practitioner (n=3); physician assistant (n=1)  *M*=45 (*SD*=?) years old  Gender: ? |  | **Control:** Booklet on general health issues + any health concerns addressed ‘in the usual manner’ | Provider: ? (n=?)  Age: ?  Gender: ? |  |  |
| Dhital (2015)  Parallel-group RCT | Community pharmacies  England | Pharmacy customers with hazardous or harmful drinking | Reduce hazardous or harmful drinking |  | AUDIT Total score  AUDIT | 3-months post-recruitment^†^ |  | **BI:** MI informed BI, resources (information leaflet, unit/ calorie calculator, alcohol services leaflet) | 10min x 1 | Single session | Pharmacist (n=17)  Age: ?^c^  Gender: ?^c^ |  | **Leaflet Only Control Condition:** Information leaflet only (‘Alcohol: The Basics’) | Pharmacist (n=17)  Age: ?^c^  Gender: ?^c^ |  |  |
| Ockene (1999)  Cluster RCT | Academic medical center affiliated primary care practices  USA | High risk drinkers (general medical population) | Reduce alcohol usage |  | Average number of standard drinks per week (past 7 days)  TLFB | 6-months^†^ |  | **Special Intervention:** Screening, standardised lifestyle interview and follow-up "patient-centred alcohol counseling sequence" focused on the number of drinks per week, binge drinking, or both, depending on the participant’s problem area(s), health booklet | 5-10 min x 1 + 1 f/up (minimum) during routine appointments | ? | Attending physician (n=15); resident physician (n=7);  nurse practitioner (n=5)  *M*=36.4 (*SD*=6.7) years old  Female: n=16 (59%) |  | **UC:** Health booklet + 'usual' alcohol intervention | Attending physician (n=11); resident physician (n=5);  nurse practitioner (n=3)  *M*=33.8 (*SD*=5.3) years old  Female: n=9 (47%) |  |  |
| Zatzick (2014)  Cluster RCT | Acute-care medical trauma centre  USA | Blood alcohol-positive in-patients with and without traumatic brain injury | Identify and intervene with problem drinkers |  | % Hazardous Drinking  AUDIT | 6^†^ and 12-^§^ months |  | **MI-enhanced Screening and BI:** Graded sequence of clinical tasks per motivation/readiness for change (elicit views on the importance/ confidence of changing, personalized feedback on alcohol use, clarify goals, e.g., to quit or cut down; and action plans) | 20-30min x 1 | Single session | Nurse (n=3)  Social worker (n=11)  Other (n=2)  Age: ?  Female: n=16 (100%) |  | **Routine Screening and BI** **(no enhanced training)**: Standard SBI with the assistance of any available guidelines, electronic or print training material, or private, state or federal resources | Nurse (n=16); social worker (n=4); other (n=4)  Age: ?  Female: n=22 (91.6%) |  |  |
| D'Onofrio (2008)  RCT | Tertiary care urban hospital emergency department  USA | Hazardous and harmful drinkers | Reduce alcohol consumption and negative consequences |  | Mean number of standard drinks per week (past 30 days)  TLFB | 6^†^ and 12-^§^ months |  | **BNI:** 4-step manual guided intervention based on MI, brief advice and behavioural contracting | 5-10min x 1 | Single session | Emergency practitioner (n=47)  Age: ?  Gender: ? |  | **Scripted Discharge Instructions:** <1min read by the provider | Emergency practitioner (n=?)  Age: ?  Gender: ? |  |  |
| Shin (2013)  RCT | Narcology services/ tuberculosis hospital  Russia | Adults starting TB treatment diagnosed with alcohol abuse or dependence | Reduce drinking and improve TB response |  | Change in mean number of abstinent days (last month)  TLFB | 3-months^†^ |  | **Brief Counselling Intervention (alone or plus naltrexone):** Adaptation of The NIH/NIAAA’s Helping Patients with Alcohol: A Health Practitioner’s Guide, embedded into standard TB appointments; alone or in combination with 50mg daily naltrexone | 10-15min x 6 (monthly) | 6-months | Physician (n=?)^c^  Age: ?  Gender: ? |  | **TAU:** Psychotherapy, disulfiram, placebo implants | Physician (n=?)^c^  nurse (?)  Age: ?  Gender: ? |  |  |
| **Sub-optimal Glycaemic Control** | | | |  |  |  |  |  |  |  |  |  |  |  | | |
| Lauffenburger (2019)  Two-arm pragmatic RCT | Health insurer/ primary care  USA | Patients with poor diabetes control | Improve HbA1c |  | Change in HbA1c.  Routine labs (most recent value) | 12-months post-randomisation^§^ |  | **Multi-faceted Intervention (ENGAGE-DM):** Semi-structured, individually-tailored telephone consultation based on the principles of BNI and shared decision making | ~30min x 1 + 3 (as needed) | 12-months? | Clinical pharmacist (n=?)  Age: ?  Gender: ? |  | **UC:** Not described | Provider: ?  (n=?)  Age: ?  Gender: ? | | |
| Juul (2014)  Cluster RCT | General practice  Denmark | People with T2D | Prevent diabetes related complications |  | HbA1c  Central Denmark Region’s Chronic Disease Database: Average of values during the ‘observational window’ (15-21 months) | 18-months post-‘core intervention’^§^ |  | **Intervention:** SDT + MI strategies (patient-provider relationships, communication skills, patient worksheets, treatment recommendations for T2D) integrated into standard care | 15-30min x 4 (quarterly) | 12-months | Practice nurses (n=?)  Age: ?  Gender: ? |  | **Usual Practice:** General practice delivered type-2 diabetes care | Practice nurses (n=?)  Age: ?  Gender: ? | | |
| Ismail (2018)  Pragmatic parallel two-arm cluster RCT | General practice (diabetes care)  England | Patients with T2D | Improve suboptimal glycaemic control |  | Change in HbA1c (mmol/mol)  Independent affinity chromatography (Primus Ultra2) | 18-months post-randomisation^§^ |  | **Diabetes-6 (D-6):** Six MI and CBT skills (active listening; managing resistance; directing change; supporting self-efficacy; addressing health beliefs; and shaping behaviours) integrated into standard care. | 30min x 12 (monthly) | 12-months | Primary care nurse (n=11)  Age: ?  Female: n=11 (100%) |  | **Standard Care:** Diabetes care per national guidance (diabetes self-management education, monitoring of biomedical status, and giving clinical information and advice) | Primary care nurse (n=12)  Age: ?  Female: n=12 (100%) | | |
| **Multiple Health Behaviour Change** | | | |  |  |  |  |  |  |  |  |  |  |  | | |
| Christian (2011)  Cluster RCT ^g^ | Community health centres  USA | Patients with increased metabolic risk factors | Increase patient health lifestyles behaviour change (healthy eating, PA) |  | Mean weight loss (lb)  Digital scale (Tanita Electronic Scale BWB-800) | 12-months^§^ |  | **Patient Education and Brief Health Behaviour-Change Intervention:** Computer assisted self-management goal setting and physician lifestyle counselling using MI, planning guide (preventing diabetes, achieving diet and PA goals) | 20min x 2 (baseline, 6-months) | 6-months | Physician (n=?)  *M*=44.0 (*SD*=6.7) years old  Gender: ? |  | **Care as Usual Control:** Usual physician visits at baseline- and 6- months + packet of health education materials | Physician (n=?)  *M*=42.3 (*SD*=7.3) years old  Gender: ? | | |
| Christian (2008)  Prospective RCT | Community health centres  USA | Patients with T2D | Increase patient health lifestyles behaviour change (healthy eating, PA) |  | Mean weight loss (lb)  Standard office scale | 12-months^§^ |  | **Brief Health Lifestyle Counselling:** Computerised assessment and tailored feedback, brief MI counselling guided by patient lifestyle change goals, planning guide (diabetes and healthy lifestyle information) | 20min x 4 (baseline, 3-, 6- & 9- months) | 9-months | Physician (n=19)  Age: ?  Gender: ? |  | **Control:** Usual physician visits x 4 (baseline, 3-, 6-, 9- months), packet of health education materials | Physician (n=?)  Age: ?  Gender: ? | | |
| Lakerveld (2013)  Parallel RCT | National primary health care services  The Netherlands | Adults at risk of T2D or CVD | Reduce the estimated risk of developing T2D and CVD mortality (PA, fruit intake, smoking) |  | 9 year risk of developing T2D:  ARIC score (ethnicity, parental history of diabetes, systolic BP, waist circumference,  and height) | 6-^†^ and 12-^§^  months |  | **Lifestyle Intervention:** MI (reinforce attitude/ intention to change; discrepancy between goal and current situation) + problem solving (overcome discrepancy, strengthen perceived control, overcome barriers) to prompt participants to find solutions to change PA, diet, or smoking (per participant preference) | 30min x 6 face-to-face (monthly) + 15min phone booster (3-monthly) | 12-months | Practice nurses (n=8)  Age: ?  Gender: ? |  | **Control:** Written information about risk of developing T2DM and CVD + existing brochures containing health guidelines regarding PA, healthy diet, and how to stop smoking. | Provider: ? (n=?)  Age: ?  Gender: ? | | |
| Heinrich (2010)  Cluster RCT | General practices  The Netherlands | Patients with T2D | Improve clinical, behavioural and process outcomes (lipids, HbA1c, BP, fruit and vegetable intake, PA, QOL) |  | HbA1c (%)  Assessment method: ? | 12- and 24-^§^ months |  | **Adapted MI:** Based on MI and health BCC, web-based education programme (designed to support self-management) | ~20min during quarterly consults | 24-months | Primary care nurses (n=18)  Age: ?  Gender: ? |  | **UC Control Group:** Standard quarterly consultations, web-based education programme (designed to support self-management) | Primary care nurses (n=15)  Age: ?  Gender: ? | | |
| Whittemore (2009)  Pilot cluster RCT | Primary care  USA | Adults at risk of T2D | Improve lifestyle behaviours: (Clinical: weight change, waist circumference, insulin resistance, lipids; Behavioural: nutrition, exercise; Psychosocial: depressive symptoms) |  | % weight loss  Assessment method: ? | 3- and 6-^† e^ months |  | **Lifestyle Change Programme:** Information, behavioural support (collaborative goal setting, problem solving barriers), MI | 20min x 11 (6 x face-to-face, 5 x phone) | 6-months | Nurse practitioner (n=?)  Age: ?  Gender: ? |  | **Enhanced Standard Care**: Standard care informed by current treatment recommendations, written information | Nurse practitioner (n=?)  Age: ?  Gender: ? | | |
| Verweij (2012)  RCT | Occupational health services  The Netherlands | Employees | Prevent weight gain/ increase PA/ decrease sedentary behaviour |  | Daily PA (moderate minutes/ week in the past month)  SQUASH | 6-months^†^ |  | **Balance @ Work:** Weight prevention guideline based care including BCC (direct advice only when asked, address resistance to change), self-monitoring, leaflets on PA and nutrition | 20-30min x 5 | 6-months | Occupational physician (n=7)  Age: ?  Gender: ? |  | **UC:** Health risk appraisal with anthropometric measurements and subsequent health advice | Occupational physician (n=9)  Age: ?  Gender: ? | | |
| Koelewijn-vanLoon (2010)  Cluster RCT | Primary care  The Netherlands | Patients at risk of CVD | Improve risk perception and lifestyle behaviours (saturated fat, fruit and vegetable, exercise, smoking, alcohol) |  | % Patients smoking  2-item ‘validated questionnaire to asses smoking status (yes v. no) | 12-months^§^ |  | **Improving Patient Adherence to Lifestyle Advice (IMPALA):** CVD Risk Management: Risk assessment, risk communication, decision support tool + adapted MI (exploring importance, confidence, goal setting) | 20min x 1 face-to-face; 10min x phone or face-to-face approx 2-weeks apart | 12-weeks | Nurse (n=13)  *M*=38 (*SD*=7) years old  Female: n=13 (100%) |  | **Minimal Intervention:** TAU (per national guidelines for cardiovascular risk management) + cardiovascular risk assessment | Nurse (n=11)  *M*=39 (*SD*=9) years old  Female: n=11 (100%) | | |
| Koelewijn-vanLoon (2009)  Cluster RCT | As above | As above | As above |  | As above | 12-weeks^†^ |  | As above | As above | 12-weeks | As above |  | As above | As above | | |
| Nanchahal (2012)  Pragmatic parallel group RCT | Primary care  England | Clinically overweight/ obese adults | Improve healthy eating, increase PA (to lose weight) |  | Weight loss from baseline to 12 months (kg)  Weight (in light clothing) using the Tanita (BC 420 MA) scale | 6- and 12-^§ e^  months |  | **Camden Weight Loss Programme (Camwel):** Informed by NICE clinical guidelines, ‘essential evidence-based components’ for behaviour change and weight loss, including MI (identify/ enhance motivation and self-efficacy) and CBT (e.g. tailored goal setting, self-monitoring, positive reinforcement, coping with lapses and high risk situations) | 30min x 14 (fortnightly x 12 weeks; 3 weekly x 12 weeks; monthly x 12 weeks) | 9-months | Nurse (n=1); osteopath (n=2);  personal fitness trainers (n=2); not specified (n=1)  Age: ?  Gender: ? |  | **Routine Clinical Practice Control**: Guided by NICE clinical guideline on obesity (e.g. dietitian referral, exercise referral, the ‘Shape-Up’ programme, weight loss medication/ surgery or no further treatment), British Heart Foundation booklet: “So you want to lose weight for good.” | Provider: ? (n=?)  Age: ?  Gender: ? | | |
| Butler (2013)  Cluster RCT | Primary care (general practice clinics)  Wales | Adults attending a primary care appointment | Change in four risk behaviours (smoking, alcohol use, exercise, and healthy eating) |  | Composite measure of self-reported beneficial change across 4 risk behaviours  DINE subset, IPAQ-SF, HIS, AUDIT-C | 3-^† e^ and 12-months |  | **Talking Lifestyles Program:** BCC training program for practitioners, to be incorporated into routine consultations (flexible, menu driven framework; guiding rather than directing, agenda setting, negotiating change, strategies to encourage participants to consider why/ how to change) | 15-30min x 1 | Single session | Nurse (n=12); GP (n=13)  Age: ?  Female: n=15 (60%) |  | **Standard Care:** Not described. | Nurse (n=14); GP (n=14)  Age: ?  Female: n=20 (71%) | | |
| Jansink (2013)  Cluster RCT | General practice (diabetes care)  The Netherlands | Patients with T2D | Improve adherence to lifestyle recommendations (diet and exercise) |  | HbA1c concentration (most recent value)  Extracted from medical records | 14-months^§^ |  | **Comprehensive Lifestyle Intervention (Provider training):** Diabetes care by providers trained in lifestyle counselling based on MI, tools to structure sessions and record keeping | 15-20min x 4 (quarterly) | 14-months | Primary care nurse (n=25)  *M*=40.7 (*SD*=7.8) years old  Female: n=23 (92%) |  | **UC:** Care consistent with current diabetes guidelines | Primary care nurse (n=28)  *M*=44.4 (*SD*=6.6) years old  Female: 27 (97%) | | |
| Bóveda-Fontán (2015)  Cluster RCT | Community health centres  Spain | Patients with dyslipidemia | Improve cardio protective behaviours (heart protective diet, PA, weight loss) and lipid level control to reduce cardiovascular risk |  | Total cholesterol  Assessment method: ? | 2-, 4-, 8- and 12-^§^ months |  | **MI:** MI-based approach in combination with clinical protocol recommendations for dyslipidemia | 7min x 1  (also at 2-, 4-, 8- and 12- months?); integrated into UC | 12-months | GP (n=18)  Age: ?  Gender: ? |  | **Standard Practice:** Advice on the necessity of changing unhealthy habits towards cardio-protective habits, according to clinical protocol recommendations for dyslipidemia | GP (n=20)  Age: ?  Gender: ? | | |
| **Other Health Behaviours** | | | |  |  |  |  |  |  |  |  |  |  |  | | |
| Godard (2011)  RCT | Department of periodontology (University Hospital)  France | Patients with periodontitis | Improve compliance with plaque control |  | Overall plaque  O’Leary Plaque Index | 1-month^†^ |  | **MI:** Guided by participant response to 5-item questionnaire (informed by Leventhal’s theory), using MI principles (clinician empathy, discrepancy between goals/values and current behaviour, lack of argumentation/confrontation), standard care and illustrated booklet | 15-20min x 1 face-to-face; 10-min x 1 phone | 1-month | Periodontist (n=2)  Age: ?  Gender: ? |  | **Standard Consultation:** Oral hygiene instruction and care per usual, illustrated booklet | Periodontist (n=2)  Age: ?  Gender: ? | | |
| Dermen (2014)  RCT | Inpatient facility  USA | Adults in inpatient treatment for alcohol use disorders | Improve oral hygiene (tooth brushing frequency per day) |  | Tooth brushing frequency (per day)  Item with six response options, converted to ‘per day’ units | 4-, 12-, and 24-^†^ weeks |  | **Brief MI-based Intervention:** Tailored information and feedback delivered using MI approach (eliciting permission; inviting the patient to share concerns, ask questions, and discuss the importance of oral health; offering to share information; eliciting reactions; and eliciting ideas and plans for change) | 30min x 1 | Single session | Dentist (n=1); dental hygienist (n=1)  Age: ?  Gender: ? |  | **Traditional Didactic Control Intervention:** Standard information provision, instruction and demonstration | Dentist (n=1); dental hygienist (n=1)  Age: ?  Gender: ? | | |
| Cornman (2008)  Pilot RCT | Urban hospital  South Africa | HIV infected patients | Reduce risky sexual behaviour |  | Total number of unprotected sex events  Structured questionnaire | 6-months^†^ |  | **Options for Health:** Based on Information-Motivation-Behavioural skill model, uses MI techniques to deliver HIV risk reduction information, motivation, and behavioural skills content | ~15min x 3-monthly | 6-months | Lay counsellor (n=3)  Age: ?  Gender: ? |  | **Standard of Care Control:** Standard HIV counseling about HIV, antiretroviral therapy, medication adherence, and nutrition | Lay counsellor (n=2)  Age: ?  Gender: ? | | |
| Hegarty (2013)  Cluster RCT | Family practice  Australia | Women experiencing intimate partner violence | Improve perceived support and comfort to discuss abuse (to enhance safety planning) |  | Quality of Life (12 months)  WHOQOL-BREF | 6-^†^ and 12-^§^ months post study invite |  | **Women’s Evaluation of Abuse and Violence Care in General Practice (WEAVE) Intervention:** Patient centred brief counselling intervention using active listening, MI, and problem-solving techniques | 30min x 1-6  (per patient need) over 6-months | 6-months | Family doctor (n=25)  *M*=49.3 (*SD*=8.4) years old  Female: n=14 (56%) |  | **UC:** Usual GP care, written resources | Family doctor (n=27)  *M*=46.9 (*SD*=7.7) years old  Female: n=18 (67%) | | |
| Fisher (2014)  Cluster RCT | HIV clinical care sites  South Africa | People living with HIV on antiretroviral therapy | Reduce HIV risk behaviour |  | Number of sexual events without a condom (last 4-weeks)  Audio computer assisted interview reported events | 6-,^†^12- and 18-^§^ months |  | **Options for Health:** Based on Information-Motivation-Behavioural skill model, uses MI techniques to assess risk behaviour; identify and address barriers; negotiate an achievable, individually tailored behaviour change (or maintenance) goal. | 10-15min x routine consultations | 18-months | Lay counsellor (n=48)  Age: ?.  Gender: ? |  | **Standard of Care Control:** Routine clinical care, including antiretroviral therapy counselling and safer sex promotion messages. | Provider: ?  Age: ?  Gender: ? | | |
| Britton (2019)  Stepped wedge cluster RCT | Hospital RT departments  Australia | Head and neck cancer patients undergoing RT | Improve nutritional status (prevent malnutrition) |  | Nutritional status (end of RT)  PGSGA | End of RT,^† e^ 1- and 3-months post-RT |  | **Eating As Treatment (EAT):** BCC and CBT incorporated into routine dietetic consultations, depression screening and referral, practice change strategies | ~20min x weekly during RT, fortnightly thereafter | 3-months | RT dietitian (n=18)  *M*=38 (*SD*=11.4) years old  Female: n=13 (92%) |  | **TAU:** Routine dietetic consultations guided by national guidelines | RT dietitian (n=24)  Age: ?  Gender: ? | | |

*Note*. ^†^ Follow-up interval included in meta-analysis of short-term outcomes; ^§^ Follow-up interval included in meta-analysis of long-term outcomes; ^a^The order of studies reflects the order of NIH fidelity checklist scores from lowest to highest; ^b^Post-randomisation unless otherwise specified ^c^ Reports overall data across conditions only; ^d^ Not included in the meta-analysis, short-term outcomes from the above study and 6-months is the longest ‘short-term’ follow-up duration reported; ^e^ Pre-specified end point; ^f^ Not included in meta-analysis (BCC intervention not compared to a control condition); ^g^ Not explicitly defined, but cluster level randomisation used; ^h^ In publications with no or multiple primary outcomes, extraction was guided by the Cochrane Community’s published definition (i.e. the outcome of greatest importance (CochraneCommunity, 2018). This was determined independently by XXX and XX by considering a) the variable of interest cited in the study aims/hypotheses and b) which outcome was reported first in the results section; ^i^Elements of the BCC intervention are summarised in Supplementary Table 2.

ACQ: Asthma Control Questionnaire; ARIC: Atherosclerosis Risk In Communities; ASI: Addiction Severity Index; ATTC: Addiction Technology Transfer Center; AUDIT: The Alcohol Use Disorders Identification Test; AUDIT-C: The Alcohol Use Disorders Identification Test-Consumption; BCC: behaviour change counselling; BI: Brief intervention; BNI: Brief Negotiation Interview; BP: blood pressure; CBT: Cognitive behavioural therapy; COPD: Chronic Onstructive Pulmonary Disease; CVD: Cardiovascular disease; DINE: Dietary Instrument for Nutrition Evaluation; ESH/ESC: European Society of Hypertension/ European Society of Cardiology; FDA: The Food and Drug Administration; GP: General Practitioner; HbA1c: Haemoglobin A1c; HIV: Human Immunodeficiency Virus; IPAQ-SF: International Physical Activity Questionnaire: Short Form; ISF: Implementation and Sustainment Facilitation; MAP: Maudsley Addiction Profile; MATCH: Matching Alcoholism Treatments to Client Heterogeneity; MET: Motivational Enhancement Therapy; MI: Motivational Interviewing; MIBI: Motivational Interviewing-based Brief Intervention; NICE: National Institute for Health & Care Excellence; NIH/NIAAA: The National Institutes of Health/ National Institute on Alcohol Abuse and Alcoholism; NRT: Nicotine Replacement Therapy; PA: Physical activity; PACIC: Patient Assessment of Chronic Illness Care; PAQ: Physical Activity Questionnaire; PGSGA: Patient Generated Subjective Global Assessment; RCT: Randomised controlled trial; RT: Radiotherapy; QOL: quality of life; SATS: Substance Abuse Treatment Scale; SDT: Self-determination theory; SQUASH: Short QUestionnaire to ASsess Health enhancing physical activity; T2D: Type 2 diabetes; TAU: Treatment as Usual; TB: tuberculosis; TLFB: Timeline Follow Back; TTM: Transtheoretical Model; UC: usual care; USA: United States of America; WHO: World Health Organisation; WHOQOL-BREF: Abbreviated World Health Organisation Quality of Life
