# Supplementary material for: Adapted motivational interviewing for brief healthcare consultations: A systematic review and meta‐analysis of treatment fidelity in real‐world evaluations of behaviour change counselling
Source: Br J Health Psychol. 2023 May 4;28(4):972–99. doi: 10.1111/bjhp.12664 (PMC10947272; doi:10.1111/bjhp.12664)
Supplement: Supplementary file 10 — Table S2 [file BJHP-28-972-s001.docx]

**Supplementary Table 2**

*Assessment and Reporting of Fidelity of Intervention Delivery Within Each of the Included Studies, Grouped According to the Health Behavior(s) Targeted by the BCC Intervention*

| **Study** | **Assessment** | | | |  | **Details of any consultations rated** | | | | | |  | **Fidelity of intervention delivery** | | |
| --- | --- | --- | --- | --- | --- | --- | --- | --- | --- | --- | --- | --- | --- | --- | --- |
|  | Instrument | Validated MI/ BCC instrument used? | Method/ details | Study arm(s) sampled |  | Number | | Selection method | Number of rater(s) | Rater training/ skills/ qualifications | Blind? |  | Validated MI/ BCC instrument reported? | Outcome(s) reported/  I vs C comparisons conducted | Level of intervention fidelity^a^ |
| **Substance Use** | |  |  |  |  | |  |  |  |  |  |  |  |  |  |
| Mitcheson et al (2007) | No fidelity assessment | --- | --- | --- |  | | --- | --- | --- | --- | --- |  | --- | --- | --- |
| Gryczynski et al (2015) | No fidelity assessment | --- | "We did not record IBI sessions or otherwise monitor fidelity, because such practices would not be typical in normal clinical delivery of IBI services" | --- |  | | --- | --- | --- | --- | --- |  | --- | --- | --- |
| Jaffray et al (2014) | Patient questionnaire (asked for feedback about interactions with pharmacists) | N | Participant self-report (6-months) | I, C |  | | --- | --- | --- | --- | --- |  | --- | % intervention vs control participants reporting a difference in their interaction with their pharmacist  I vs C compared using Fisher’s exact test | Intervention 13.1% (n=43) vs control 6.4% (n=21) said the ‘pharmacist spoke more’ (p=0.056); and this discussion was useful (intervention 50.6%, n=44 vs control 35.6%, n=31; p =0.047) |
| Mertens et al (2014) | BECCI | Y | Rating of audio recorded consultations | I |  | | Sample of tapes used during supervision | Random | 1? | ‘Experienced practitioner and trainer' | N (trainer/ supervisor) |  | N | --- | Findings not reported |
| Garner et al (2020) | ITRS | Y | Rating of audio recorded consultations | I |  | | ? | ? | 15 | The lead developer of the MIBI protocol (co-author SM) | Y |  | Y | Overall ‘sum quality score’ (standardised score incorporating adherence and competence for all staff) | MIBI + ATTC:  *M*=560 (*SD*=780)  MIBI + ATTC + ISF: *M*=1324 (*SD*=1054). |
| Darker et al (2016) | Modified version of BNI Adherence and Competence Scale (23 items) | N | Rating of audio recorded consultations | I |  | | 1 per random selection of providers | Providers randomly selected for 1 x randomly selected BI session to be audio-recorded (within 1 month post-training and >2 BI sessions completed). | 1 | Study author (LW) | ? |  | --- | Number of fidelity checks within the BNI scale competence categories | “All intervention fidelity checks except for one were in the ‘perfect’ category on the modified BNI Adherence Scale with the remaining one being in the ‘acceptable’ category.” |
| **Physical Activity** | |  |  |  |  | |  |  |  |  |  |  |  |  |  |
| Jackson et al (2007) | Fidelity not assessed | --- | --- | --- |  | | --- | --- | --- | --- | --- |  | --- | --- |  |
| Elley et al (2003) | Follow-up interview (recollection of receiving a green prescription during the previous year). | N | Participant recall (12-month follow-up) | I, C |  | | --- | --- | --- | --- | --- |  | --- | Proportion of I and C patients ‘yes’  No between groups comparison | Intervention 95% (370/ 389) and Control 3% (10/361) |
| Dennett et al (2018) | MITI | Y | Rating of role-play (commencement and mid-point) | I |  | | 1 x trial commencement; 1 x mid-way | ? | ? | 1? | ‘independent’ |  | N | Description only (no data) | “rated the physiotherapist as ‘competent’ on the global clinician rating at baseline and ‘competent to proficient’ at mid-intervention”. |
| O’Halloran et al (2016) | MITI | Y | Rating of role-play (commencement and mid-point) | I |  | | 2? | ? Selected from intervention commencement and midpoint | 1? | ? | ‘independent’ |  | N | Description only (no data) | “The physiotherapist’s proficiency in using MI was rated as competent on the global clinician rating at both assessments. There was improvement on two of counts (a percentage of open questions and reflection to question ratio) from beginning proficiency to competency at the midpoint of the intervention” |
| van der Weegen et al (2015) | Session checklists (session content) | N | Provider self-report (after every session) | I |  | | --- | --- | --- | --- | --- |  | --- | Proportion of participants who received the intervention ‘as intended’ | 83% |
|  | Participant interviews (intervention experience) |  | Participant recall (9-month follow-up) |  |  | |  |  |  |  |  |  |  |  |  |
| **Smoking** |  |  |  |  |  | |  |  |  |  |  |  |  |  |  |
| Louwagie et al (2014) | Fidelity not assessed | --- | --- | --- |  | | --- | --- | --- | --- | --- |  | --- | --- | “Unable to monitor intervention fidelity by video-taping actual MI sessions with real patients, as it is considered invasive to patients within the context of standard clinical care” |
| Cabezas et al (2011) | ‘Quality' and 'compliance' assessment (tool not specified) | N | Rating of audio recorded consultations | I |  | | ? | ‘Recorded randomly' | ? | ? | ? |  | --- | --- | Findings not reported |
| Meyer et al (2012) | Fidelity not assessed (only number of interventions delivered) | --- | --- | --- |  | | --- | --- | --- | --- | --- |  | --- | --- | --- |
| Cossette et al (2012) | ‘Intervention grid' (42 potential intervention elements) | N | Unclear if provider self-report or researcher rated | I |  | | ? | ? | ? | ? | ? |  | --- | Number of patients receiving each individual intervention element within each of the six intervention sessions. | Highest: 95% (n=?) ‘interventions targeting conviction and confidence’  Lowest: 0% (n=0) ‘interventions in the pre-contemplation and maintenance stages’ |
| Glasgow et al (2000) | Tool not specified | N | “Initially, research staff were present in the clinics daily to observe interventions and protocol adherence and to provide feedback” | I, C |  | | --- | --- | --- | --- | --- |  | --- | % of patients receiving intervention components  No between groups comparison | Highest: 93% (n=?) ‘counselling’  Lowest: 10% (n=?) ‘both follow-up calls’ |
| Ershoff et al (1999) | Session checklist (date and length of call, stage of change, topics discussed) | N | Provider self-report (after every session) | I |  | | --- | --- | --- | --- | --- |  | --- | --- | Findings not reported |
| Butler et al (1999) | Telephone interview (describe their standard practice when using the method) | N | Provider recall (2-months post training, n=24 providers) | I |  | | --- | --- | --- | --- | --- |  | --- | Number of providers using each of the 7 intervention components (% yes for each individual component). | Highest: 79.1% (n=19) ‘assessing motivation and confidence’  Lowest: 16.6% (n=4) ‘non-judgemental information sharing’; ‘Identifying and responding to the patients biggest worry about stopping smoking’ |
| Borrelli et al (2005) | Supervision (tool not specified) | N | Supervision on live and audio-recorded consultations (when?) | I? |  | | ? | ? | ? | ? | ? |  | --- | --- | Findings not reported |
|  | Patient exit interviews |  | Participant recall (when?) |  |  | |  |  |  |  |  |  |  |  |  |
|  | ‘Documentation’ (time spent discussing smoking and components delivered) |  | Provider self-report (every session?) |  |  | |  |  |  |  |  |  |  |  |  |
| Hollis et al (2007) | Tool not specified (‘adherence to key elements of each protocol’) | N | Rating of audio recorded consultations | I, C |  | | ? | ? | ? | ? | ? |  | --- | --- | Findings not reported |
| **Treatment Adherence/ Engagement** | |  |  |  |  | |  |  |  |  |  |  |  |  |  |
| Leiva et al (2014) | ‘Application of the different components of the multifactorial intervention’ (tool not specified) | N | ? | I |  | | --- | --- | --- | --- | --- |  | --- | % (n) patients receiving each intervention element | Highest: 66.7% (n=70) ‘participated in all 3 MI sessions’  Lowest: 5.7% (n=6) ‘had dose regimes simplified’ |
| Eyler et al (2016) | Fidelity not assessed | --- | --- | --- |  | | --- | --- | --- | --- | --- |  | --- | --- | --- |
| Drevenhorn et al (2012) | Patient centredness' (3 point Likert scale) | N | Rating of audio recorded consultations (pre- post- training) | I |  | | 36 pre, 35 post training | Appointment 1 before training and within 3-months post-training | ? | Researchers (ED, AB, KK) | Unlikely |  | --- | Total number of times each patient centredness and utterance item was rated ‘yes’ before and after training for each provider | Highest: 100% (n=35/35) ‘mentioning exercise’  Lowest: 62% (22/35) ‘mentioning blood lipids’ |
|  |  |  |  |  |  | |  |  |  |  |  |  |  | % consultations met ‘all the criteria for paying attention to a patient’s readiness to change’ | 25% (n=9/35) |
| Cook et al (2017) | MISC | Y | Rating of audio recorded consultations | I, C |  | | ? (10% random sample) | random | 2? | Experienced mental health professional and MI trainer, attended a coding group to maintain reliability. | Y |  | Y | % or M(SD) for MI-consistent statements, % reflection; % open questions; % direct instruction;  I scores relative to benchmarks | MI skills:  Intervention *M*=5.77(*SD*=0.47) and Control: *M*=2.57 (*SD*=0.86)  Mean intervention score exceeded the competence benchmark (5.07) |
| Graham et al (2016) | Tool not specified | N | “During supervision, the standard of delivery was regularly monitored and assessed for fidelity and adherence” | ? |  | | ? | ? | ? | ? | ? |  | --- | --- | Findings not reported |
| Hedegaard et al (2016) | Modified version of the MITI | N | Audio-recordings 'reviewed three times during the study' | I |  | | ? | ? | 2 | consulting pharmacist and a second pharmacist | ? |  | --- | MITI data not presented | --- |
|  | Drug related problems and actions taken (tool not specified) |  | Unclear how session content captured | ? | ? | |  | ? | ? | ? | ? |  | --- | M(SD), range for overall number of intervention elements delivered | Actions delivered *M*=3.4(*SD*=1.9) Range between pharmacist=2.7 to 4.6 actions per patient |
|  |  |  |  |  |  | |  |  |  |  |  |  |  | % patients who received each individual step of the intervention | Highest: 93.6% (n=315) ‘MI’  Lowest: 2.6% (n=4) ‘Cost reduction’ |
| George et al (2021) | BNI Adherence and competence Scale | N | Rating of audio recorded consultations | I,C |  | | ? | Uploaded by providers | ? | ? | ? |  | --- | % consultations addressing each individual step of the intervention | Highest: 96.6% (n=?) ‘Step 2’  Lowest: 73.7% (n=?) ‘Step 4’ |
| **Alcohol** |  |  |  |  |  | |  |  |  |  |  |  |  |  |  |
| Bager et al (2010) | No fidelity assessment | --- | --- | --- |  | | --- | --- | --- | --- | --- |  | --- | --- | --- |
| Noknoy et al (2010) | No fidelity assessment | --- | --- | --- |  | | --- | --- | --- | --- | --- |  | --- | --- | Absence of fidelity assessment acknowledged as a study limitation |
| Aalto et al (2000; 2001) | No fidelity assessment | --- | --- | --- |  | | --- | --- | --- | --- | --- |  | --- | --- | --- |
| L'Engle et al (2014) | “Data, assessment, and plan form” (session counselling notes) | N | Provider self-report (each session) | I |  | | ? | ? | ? | ? | ? |  |  | --- | Findings not reported |
|  | Tool not specified |  | Direct observation (monthly) |  |  | |  |  |  |  |  |  | --- |  |  |
| Schaus et al (2009) | ‘Competency assessment' (tool not specified) | N | Rating of audio recorded consultation (monthly) | I |  | | ? | ? | ? | Mental health counselor trained in MI | ? |  | --- | --- | Findings not reported |
|  | 'Participant comment form’ (provider empathy and MI statements) |  | Participant self-report (after every session) | I |  | | --- | --- | --- | --- | --- |  | --- | --- |  |
|  | 'Provider comment form’ (MI skills and BASICS components) |  | Provider self-report (after ever session) | I |  | | --- | --- | --- | --- | --- |  | --- | --- |  |
| Fleming et al (2010) | Form "to document that patients had received the protocol and had agreed to reduce their alcohol use." | N | Provider self-report (post-intervention session) | I |  | | --- | --- | --- | --- | --- |  | --- | --- | Findings not reported |
| Dhital et al (2015) | Telephone interview: ‘Asked if they recalled having a discussion with the pharmacist about their drinking following the AUDIT questions’ | N | Participant recall  (3-months) | I, C |  | | --- | --- | --- | --- | --- |  | --- | % ‘yes’ presented for intervention and control participants  No between groups comparison | Intervention: 77% (n=130); |
| Ockene et al (1999) | Standardised patient exit interview (which 15 intervention steps were received) | ? | Participant recall (post-consultation) with a random subset (N=344; 65%) of participants | I, C |  | | --- | --- | --- | --- | --- |  | --- | M % yes for intervention vs control participants  Mixed-model analysis of covariance | Intervention *M*=9.8(*SD*=?) vs Control *M*=1.7(*SD*=?), p=0.0001 |
| Zatzick et al (2014) | MITI | Y | Rating of 7 x 20min standardised patient-actor telephone interviews per provider (Baseline + 1-week, 1, 4, 7, 17 and 27 months) | I, C |  | | 30 | Standardised scenarios at pre-determined assessment time points | 2 (every 5th recording rated twice for IRR) | Trained by expert trainer (CD) | ? |  | Y | 5 x MITI domains ((% open ended questions; % complex reflections; Reflection/ question ratio; % MI adherence; Global MI spirit) for intervention and control providers at baseline and 6 x f/up time points.  I vs C compared using mixed effects hierarchical regression models | % MI adherence  Highest: *M*=90.4(*SD*=22)  Lowest: *M*=58.8(*SD*=42.1)  Intervention vs Control: significantly higher % MI adherence (F(6,153)=4.69,p=0.0002) |
| D'Onofrio et al (2008) | BNI Adherence and Competence scale | N | Rating of audio recorded consultations | I, C |  | | 25 (reliability) + 367 | ? | 3 | Trained for reliability (4-hour training session). | Y |  | --- | Mean BNI adherence score for intervention and control providers  Between groups analysis not specified | Intervention: *M*=8.9 out of 13(*SD*=?) |
| Shin et al (2013) | ‘Fidelity assessment measure’ (adapted from BECCI)  Brief form’ (TAU) | N | Rating of audio recorded consultations | I, C |  | | 10% of recorded sessions | ? | 2 | RA’s trained by trainer and US co-investigator; rating of mock-interviews used to certify raters | ? |  | --- | % of encounters 'meeting criteria for procedural adherence'  No between groups comparison | Intervention 98% (n=?) |
| **Sub-optimal Glycaemic Control** | |  |  |  |  | |  |  |  |  |  |  |  |  |  |
| Lauffenburger et al (2019) | Clinical notes (intervention components) | N | Researcher rating of session content |  |  | | --- | --- | --- | --- | --- |  | --- | --- | Findings not reported |
| Juul et al (2014) | ‘Evaluation questionnaire’ (perceived autonomy supportive competences before and after training; use of tools) | N | Provider self-report (11months post workshop) | I |  | | --- | --- | --- | --- | --- |  | --- | The number of providers scoring 1(rarely) through to 10 (always) before and after training for all 14 items (descriptive counts) | 70% (n=19/27) “reported perceived improvement in at least three of the autonomy supportive competences” |
|  | HCCQ (perceived autonomy support) |  | Participant self-report (~18-months follow-up) | I, C |  | |  |  |  |  |  |  |  | I vs C HCCQ: M (95% CI), mixed additive model | Intervention: *M*=6.2(95% CI=5 to 7) vs Control: *M*=6.0(95% CI=5 to7), p=0.43 |
| Ismail et al (2018) | MITI | Y | Rating of audio recorded consultations | I, C |  | | 266 recordings (from 151 patients and 17 nurses) | Stratified by nurse and patient; random selection (of ≥20 minutes) x 3 patients per nurse from session 2, 3 and 4. | 2 (1 rater per scale) | ‘Suitable training for whichever scale they used' | Y |  | Y | MITI M(SD) or Median (IQR) for Global spirit, Global empathy, Proportion complex reflections, Proportion open questions, Reflection/ Question ratio, % MI adherent | % MI adherent:  Intervention *M*=0.58(*SD*=0.32) vs. Control *M*=0.54(*SD*=0.28), p=0.51 |
|  | BECCI | Y | As above | As above |  | | As above | As above | As above | As above | As above |  | Y | BECCI M(SD) | Intervention *M*=1.33(*SD*=0.56) vs. Control *M*=1.12(*SD*=0.55), p=0.12 |
|  |  |  |  |  |  | |  |  |  |  |  |  |  | I vs C compared using T-test or Mann-Whitney U-test |  |
| **Multiple Health Behavior Change** | |  |  |  |  | |  |  |  |  |  |  |  |  |  |
| Christian et al (2011) | Fidelity not assessed | --- | --- | --- |  | | --- | --- | --- | --- | --- |  | --- | --- | --- |
| Christian et al (2008) | Fidelity not assessed | --- | --- | --- |  | | --- | --- | --- | --- | --- |  | --- | --- | --- |
| Lakerveld et al (2013) | MITI | Y | Rating of audio recorded consultations | I |  | | 2 x sessions of ≥15 minutes from all practice nurses + 10 extra | random (using a computerized randomization program) | 2 | Researchers | ? |  | Y | M (range) for coder 1 and coder 2 for reflection question ratio; % MI adherence; empathy; MI spirit; direction | % MI adherence:  Coder 1: *M*=94.5 (Range=87-100)  Coder 2: *M*=93.2 (Range=82.6-8.9) |
|  | MISC (% Change talk) | Y | As above | As above |  | | As above | As above | As above | As above | As above |  | Y | M (range) for coder 1 and coder 2 | Coder 1: *M*=58.2 (Range=10-83)  Coder 2: *M*=74.8 (Range=62-82) |
|  | PSCC | N | As above | As above |  | | As above | As above | As above | As above | As above |  | --- | M (range) for coder 1 and coder 2 | General therapeutic skills/ problem-solving skills  Coder 1: *M*=2.93 (Range=1-4)/ *M*=2.11 (Range=1-4)  Coder 2: *M*=3.33 (Range=2-4)/ *M*=2.84 (Range=1-4) |
| Heinrich et al (2010) | Tool not specified | N | Rating of audio recordings | I? |  | | 2 + 3 x direct observation | ? | ? | ? | Unlikely |  | --- | --- | Findings not presented |
|  | Tool not specified | N | Direct observation (6, 9, 11-months post training) |  |  | |  | ? | 2 x observers (MI-trained researcher/ MI-trained teaching nurse) | MI-trained researcher/ MI-trained teaching nurse | N |  | --- | --- | Findings not presented |
| Whittemore et al (2009) | “Session documentation forms” (session components itemised) | N | Provider self-report (after every session) | I |  | | --- | --- | --- | --- | --- |  | --- | % implementation (number of components delivered/ number of components to deliver) | 76% (n=?) |
| Verweij et al (2012) | BECCI | Y | Rating of audio recorded consultations | I |  | | 3 per provider | Unclear how the consultations themselves were selected, but 10minutes were randomly selected and scored | 1 | 1 ( MI trainer) | N |  | Y | Mean BECCI score | *M*=2.1(*SD*=?) |
|  | ‘Matrix of performance indicators’ | N | Researcher rated from provider completed 'registration forms' | I |  | | --- | --- | --- | --- | --- |  | --- | % ‘guideline adherence’ | 47% (n=?) |
| Koelewijn-van Loon et al (2010; 2009) | Tool not specified | N | Rating of audio recorded consultations | I |  | | 3 per provider | 1 x initial, two later (nurse selected?) | ? | ? | ? |  | --- | --- | Findings not reported |
|  | ‘Short standardised questionnaire’ (intervention components done/ not done) | N | Provider self-report (after every session) | I |  | | --- | --- | --- | --- | --- |  | --- | --- | Findings not reported |
| Nanchahal et al (2012) | MITI | Y | Rating of audio recorded consultations (22/104) from intervention mid-point | I |  | | 22 (104 recordings from 42 participants: 34 had final weight outcomes; 27 had all 3 mid-intervention sessions; 22 were sufficient quality) | ? | 3? | Authors LA, GB and LN | Y |  | Y | MITI M(SD) overall for reflection/question ratio, % open questions, % complex reflections, % MI adherent, all relative to proficiency benchmarks (also M(SD) rater one and rater 2 for evocation, collaboration, autonomy/ support, direction, empathy, spirit and behavior counts) | % MI adherent *M*=53.1%(*SD*=17.2%), below ‘beginning proficiency’ |
|  | Study checklists (scheduled topics and activities) | N | As above | As above |  | | As above | As above | As above | As above | As above |  | --- | % core scheduled content delivered within each session and overall (based on 8-items common to each session) | *M*=41% (SD=?) of scheduled content  Highest: 98% (n=?) ‘taking weight and waist measurements’  Lowest: 8% (n=?) ‘reviewing participants use of the previous session’s topic and handouts’ |
|  | Patient Assessment of Care for Chronic Conditions |  | Participant recall (12-month follow-up) | I |  | | --- | --- | --- | --- | --- |  | --- | Intervention M(SE) PACIC score for patient activation, delivery system design/ decision support, goal setting, problem solving/ contextual counselling subscale scores | Highest: Intervention *M*=3.29(*SE*=0.17) vs control *M*=1.46 (*SE*=0.13), p<.0001 ‘patient activation’  Lowest: Intervention *M*=3.34(*SE*=0.16) vs control *M*=1.66(*SE*=0.15), p<.0001 ‘contextual counselling’ |
| Butler et al (2013) | BECCI | Y | Rating of simulated consultations (within 1-month and 7-8-months post-training) | I (providers) |  | | ? | ? | ? | Facilitator trained in BCC. | ? |  | Y | BECCI (M) for both time-points;  Number of providers scoring ‘some extent or greater’ benchmark | <1 month post training *M*=1.196 (*SD*=?) vs. f/up *M*=1.566 (*SD*=?); p=0.140;  50% (n=11) > 'some extent' |
|  | Tool not specified | N | Participant recall of discussion about behavior change | I, C (patients) |  | | --- | --- | --- | --- | --- |  | --- | I vs C % participants ‘yes’ | Intervention 91.1% (n=724) vs Control 55% (n=531) recalled behavior change discussion (OR=12.44, 95% CI=5.85 to 26.46). |
| Jansink et al (2013) | BECCI  Checklist comprising MITI (global empathy, global spirit and global structure in consultation) and 10 MI items addressed during training | Y | Rating of video consultations (baseline vs 1-year) | I, C |  | | 75 x intervention; 100 x control | Nurse self-selected (5 pre, 5 post per provider) | ? | The first author (RJ) trained two judges (CS and NV) | yes |  | Y | M(SD) global score and individual items for intervention and control providers at baseline and 1-year f/up  Intervention vs control difference in change between groups: multilevel linear regression analysis | BECCI Intervention baseline *M*=1.53(*SD*=0.47) vs f/up *M*=1.63(*SD*=0.65) and Control baseline *M*=1.49(*SD*=0.45) vs f/up *M*=1.42(*SD*=0.52); p=0.237  Checklist Intervention baseline *M*=1.21(*SD*=0.25) vs f/up *M*=1.42(*SD*=0.36) and Control baseline *M*=1.36(*SD*=0.23) vs f/up *M*=1.24, *SD*=0.23); p=0.133 |
|  | ‘Record keeping’ (degree to which the nurses work according to the MI principle) | N | Provider self-report (every session) | ? |  | | --- | --- | --- | --- | --- |  | --- | ? | Findings not presented |
|  | Medical files (diabetes check-ups) |  | Chart review (14-months) | ? |  | | --- | --- | --- | --- | --- |  | --- | ? | Findings not presented |
| Bóveda-Fontán et al (2015) | Motivational Interviewing Assessment Scale | Y | Rating of video recorded consultations (2-4-8-12-month visits) | I, C |  | | 4 x sessions x 1 patient per provider | Based on pre-determined follow up periods 2,4,8 and 12 months | GPs and 'researchers' | ? | ? |  | Y | Mean EVEM score for intervention providers before vs. after training and 1^st^ vs final patient visit. | Intervention *M*=23.63(*SD*=?) pre- vs. *M*=38.57(*SD*=?) post; p < 0.001  Intervention *M*=22.51(*SD*=?) 1^st^ visit vs. *M*=24.96(*SD*=?) final visit; p = 0.023 |
| **Other Health Behaviors** | | |  |  |  |  | |  |  |  |  |  |  |  |  |
| Godard et al (2011) | Fidelity not assessed | --- | --- | --- |  | --- | | --- | --- | --- | --- |  | --- | --- | --- |
| Dermen et al (2014) | MITI | Y | Rating of audio recorded consultations | I, C |  | 80 (10 per condition-practitioner combination) | | ? | ? | ‘expert coders' | yes |  | N | --- | Findings not reported |
| Cornman et al (2008) | Patient record form (session content) | N | Provider self-report (after every session) | I |  | --- | | --- | --- | --- | --- |  | --- | % of sessions that implement 7 of 8 steps | *M*=7.2(*SD*=?), Mode=8; IQR=1.0 |
|  | Patient exit interviews (intervention implementation) | N | Participant recall (N=20; 6-months) |  |  |  | |  |  |  |  |  |  |  |  |
| Hegarty et al (2013) | Tool not specified | N | Rating of audio recorded consultations | I, C |  | ? | | ? | ? | ? | ? |  | --- | --- | Planned, but no findings reported |
|  | Encounter forms (what GPs did) |  | Provider self-report (during each consultation) | I |  | --- | | --- | --- | --- | --- |  | --- | --- |  |
|  | Semi-structured interviews (satisfaction, changes to usual care; for providers only: impact on practice and sustainability of skills) |  | Provider and participant recall (12-months) | I, C |  | --- | | --- | --- | --- | --- |  | --- | --- |  |
| Fisher et al (2014) | ‘Options for Health’ record form (session content) | N | Provider self-report (after every session) | I |  | --- | | --- | --- | --- | --- |  | --- | --- | Findings not reported |
|  | Patient exit questionnaire (session content) | N | Participant self-report (after every session) | I |  | --- | | --- | --- | --- | --- |  | --- | --- | Findings not reported |
| Britton et al (2019) | BECCI (MI) | Y | Rating of audio recorded consultations | I, C |  | 20% (control n=196; intervention n = 194). | | Stratified randomisation | 2 (inter- and intra-rater reliability assessed) | Trained to pre-sepecified benchmarks before rating | Y |  | Y | BECCI (M,SD)  % sessions meeting benchmark (M=2.57) | BECCI: Intervention *M*= 2.14 (*SD*=0.42) vs Control *M*=2.01 (*SD*=0.39), p <.0001  Benchmark: Intervention 15.5% (n=30) vs 6.1% (n=12), p=.0001 |
|  | Study checklist (study specific components) | N |  |  |  |  | |  |  |  |  |  |  | % sessions rated ‘yes’  for each of 6 key skills  (relative to 80% benchmark) | Highest: Intervention 45.3% (n=88) vs Control: 25% (n=49), p<.001 ‘eating as integral to RT treatment’  Lowest: Intervention 18.5% (n=30) vs Control 1.2% (n=2), p=0.213 ‘review of the nutrition plan’ |
|  | CTS-R items (competence and interpersonal effectiveness) | N |  |  |  |  | |  |  |  |  |  |  | CTS-R (M,SD) | Intervention *M*=2.72(*SD*=1.34) vs Control *M*=2.21(*SD*=1.08), p<.001 |
|  |  |  |  |  |  |  | |  |  |  |  |  |  | % sessions meeting benchmark (M=3) | Benchmark: Intervention 56.2% (n=109) vs Control 35.3% (n=69), p<.001 |
|  |  |  |  |  |  |  | |  |  |  |  |  |  | I vs C compared using multiple logistic regression |  |

*Note*. ^a^When multiple values are presented findings reported are limited to the highest and lowest levels of fidelity reported for that outcome and any between group comparisons (i.e. differentiation)

ATTC: Addiction Technology Transfer Center; AUDIT: The Alcohol Use Disorders Identification Test; BASICS: Brief Alcohol Screening and Intervention for College Students; BCC: Behavior Change Counselling; BECCI: Behavior Change Counselling Index; BNI: Brief Negotiation Interview; C: Control; CTS-R: Cognitive Therapy Scale-Revised; EVEM: Motivational Interviewing Assessment Scale; GP: General Practitioner; HCCQ: The Health Care Climate Questionnaire; I: Intervention; IRR: Inter-rater reliability; ISF: Implementation and Sustainment Facilitation; ITRS: Independent Tape Rater Scale; M: Mean; MI: Motivational Interviewing; MIBI: Motivational Interviewing-based Brief Intervention; MISC: Motivational Interviewing Skill Code; MITI: Motivational Interviewing Training Integrity scale; PACIC: The Patient Assessment of Chronic Illness Care; SD: Standard Deviation; TAU: Treatment as usual;
