# Supplementary material for: Adapted motivational interviewing for brief healthcare consultations: A systematic review and meta‐analysis of treatment fidelity in real‐world evaluations of behaviour change counselling
Source: Br J Health Psychol. 2023 May 4;28(4):972–99. doi: 10.1111/bjhp.12664 (PMC10947272; doi:10.1111/bjhp.12664)
Supplement: Supplementary file 11 — Table S3 [file BJHP-28-972-s003.docx]

**Supplementary Table 3**

*Summary of BCC Components^‡^ Mentioned and Definition(s) Provided, Grouped According to the Health Behavior(s) Targeted by the BCC Intervention*

|  | Goals | | | | |  | Style | | |  | Skills | | | | | | | | | | | |  | Definition(s) |
| --- | --- | --- | --- | --- | --- | --- | --- | --- | --- | --- | --- | --- | --- | --- | --- | --- | --- | --- | --- | --- | --- | --- | --- | --- |
|  | **Establish rapport** | **Identify client goals** | **Exchange information** | **Choose strategies based on client readiness** | **Build motivation for change** |  | **Empathic** | **Collaborative** | **Active participant** |  | **Open questions** | **Affirmations** | **Summaries** | **Ask permission** | **Encourage recipient choice and responsibility in decision making** | **Provide advice** | **Reflective Listening** | **Directive use of reflective listening** | **Variation in depth of reflections** | **Elicit change talk** | **Roll with resistance** | **Elicit values** |  |  |
| **Substance Use** | | | |  |  |  |  |  |  |  |  |  |  |  |  |  |  |  |  |  |  |  |  |  |
| Mitcheson (2007) |  | Y |  | Y | Y |  |  | Y | Y |  | Y |  |  |  | Y |  |  |  |  | Y |  |  |  | “MI is a counselling approach aimed at exploring and resolving clients’ ambivalence about behaviour change [16, 17] . MI and its adaptations tend to be relatively brief in com-parison with other addiction interventions. It may be targeted at assisting clients to make a decision to change some aspect of their drug-using behaviour, rather than itself providing skills to maintain change. It aims to promote reflection on drug use and personal consequences in the context of the individual’s goals and values.” (p7) |
| Gryczynski (2015) |  |  | Y^a^ | Y | Y |  | Y |  | Y |  | Y | Y^b^ |  |  | Y |  |  |  |  |  |  |  |  | “Brief interventions are designed to be short but potent encounters that can catalyze motivation and behaviour change”(p3) |
| Schwartz (2014) |  |  | As above | As above | As above |  | As above |  | As above |  | As above | As above |  |  | As above |  |  |  |  |  |  |  |  | As above |
| Jaffray (2014)^c^ |  |  |  |  | Y |  |  | Y |  |  |  |  |  |  |  |  |  |  |  |  |  |  |  | “Related to the transtheroretical (TIM) model of change,[3] MI is ‘a collaborative person-centred form of guiding to elicit and strengthen motivation for change’.[2]”(p5) |
| Mertens (2014) |  |  |  |  |  |  |  |  |  |  |  |  |  |  |  |  |  |  |  |  |  |  |  | No definition, intervention description/ rationale only† |
| Garner (2020) | Y | Y | Y^a^ | Y | Y |  |  | Y | Y |  | Y |  | Y |  | Y |  | Y | Y |  | Y |  |  |  | No definition, intervention description/ rationale only |
| Darker (2016) |  | Y | Y^a^ | Y | Y^d^ |  | Y | Y | Y |  |  | Y^b^ | Y |  | Y | Y |  |  |  | Y | Y |  |  | “BIs are based upon motivational interviewing (MI) principles’ (Miller & Rollnick,2002), stages of change (Prochaska & DiClemente,1982) and also the FRAMES technique (Bien et al.,1993). BIs constitute a variety of activities characterized by their low intensity and short duration (Bien et al.,1993). Elements of a BI include (1) give screening results; (2) identify risks and discuss consequences; (3) provide advice; (4) solicit patient commitment; (5) identify goal; (6) give advice and encouragement” (p1105) |
| **Physical Activity** | | | | |  |  |  |  |  |  |  |  |  |  |  |  |  |  |  |  |  |  |  |  |
| Jackson (2007) |  | Y | Y^a^ | Y | Y |  |  | Y |  |  |  | Y |  |  |  | Y |  |  |  | Y |  |  |  | No definition, intervention description/ rationale only |
| Elley (2003) |  | Y | Y^a^ |  |  |  |  | Y |  |  |  |  | Y^f^ |  |  | Y |  |  |  |  |  |  |  | No definition, intervention description/ rationale only |
| Dennett (2018) | Y | Y | Y^a^ | Y | Y |  |  |  |  |  |  | Y^b^ |  |  |  |  |  |  |  | Y |  |  |  | “MI is a patient-centred style of behavioural counselling that aims to increase physical activity through addressing ambivalence about behaviour change.13 It differs from other behaviour change interventions, such as health coaching, because the primary emphasis is on people producing their own arguments for change.13” (p256) |
| O’Halloran (2016) |  |  |  |  | Y |  |  | Y |  |  |  |  |  |  |  |  |  |  |  | Y |  |  |  | “MI is a directive style of communication where the person delivering the intervention works collaboratively with the client to assist them to increase his or her motivation to change their behaviour” (p1110) |
| Van der Weegen (2015) |  | Y | Y^a^ | Y | Y |  |  | Y |  |  |  | Y^b^ |  |  |  | Y |  |  |  | Y |  |  |  | “Motivational interviewing is considered a positive way of approaching people with chronic diseases” (Verwey et al., 2016; p349) |
| Smoking |  |  |  |  |  |  |  |  |  |  |  |  |  |  |  |  |  |  |  |  |  |  |  |  |
| Louwagie (2014) |  | Y |  | Y | Y^d^ |  | Y^e^ |  |  |  |  |  |  |  |  | Y |  |  |  |  |  |  |  | “MI is a ‘directive, client-centred counselling style foreliciting behaviour change by helping clients to exploreand resolve ambivalence’ [31]… ‘Brief MI’ is a short form developed for busy clinical settings.” (p1944) |
| Cabezas (2011) |  | Y | Y^a^ | Y | Y^d^ |  |  |  |  |  |  | Y^b^ |  |  |  | Y |  |  |  |  |  |  |  | “MI helps people to explore and resolve ambivalence about behaviour change.” (p1696) |
| Meyer (2012) |  |  |  |  |  |  |  |  |  |  |  |  |  |  |  |  |  |  |  |  |  |  |  | No definition, intervention description/ rationale only^†^ |
| Cossette (2012) |  | Y | Y^a^ | Y | Y |  |  | Y |  |  |  | Y |  |  |  |  | Y | Y |  | Y |  | Y^f^ |  | “With this approach [MI], smokers’ motivation towards smoking cessation can be evaluated by both their perceived level of conviction to quit smoking, as well as their perceived level of confidence in being able to quit...Studies testing the effect of motivational interviewing on behavioural change often include interventions that draw on the Stages of Change model (Tomlin & Richardson, 2004).” (p17) |
| Glasgow (2000) |  | Y | Y | Y |  |  | Y^e^ |  |  |  | Y |  |  |  |  | Y |  |  |  |  |  |  |  | No definition, intervention description/ rationale only |
| Ershoff (1999) |  | Y |  | Y | Y^d^ |  | Y^e^ | Y | Y |  |  |  | Y^f^ |  |  | Y | Y |  |  | Y |  |  |  | “MI has been defined as ‘[a] directive, client-centered counseling style for helping clients explore and resolve ambivalence about behavior change.’23” (p164) |
| Butler (1999) |  | Y | Y | Y | Y |  | Y^e^ |  | Y |  |  |  |  |  |  |  | N | N |  | Y |  |  |  | “Motivational consulting is based on inviting patients to numerically rate their motivation and confidence to quit smoking (phase 1). Clinicians respond to these scores using specific questions and strategies (phase 2). The aim is to build motivation or confidence by encouraging the patient to identify arguments for change (motivation) or practical, attainable steps for quitting (confidence). Finally, patients are invited to set meaningful targets for themselves (phase 3).24” (p612) |
| Borrelli (2005) |  | Y | Y |  | Y |  | Y^e^ |  | Y |  | Y | Y |  |  | Y |  |  | Y |  | Y |  | Y |  | “ME was based on the principles of Motivational Interviewing (Miller and Rollnick, 1991, 2002), delivered in a client-centered manner and focused on exploring and resolving ambivalence about quitting smoking.” (p817) |
| Hollis (2007) | Y | Y |  |  | Y |  | Y^e^ |  |  |  |  |  |  |  |  | Y |  |  |  |  |  |  |  | No definition, intervention description/ rationale only |
| **Treatment Adherence/ Engagement** | | | | | |  |  |  |  |  |  |  |  |  |  |  |  |  |  |  |  |  |  |  |
| Leiva (2014)^c^ |  |  |  |  | Y^d^ |  |  |  |  |  |  |  |  |  |  |  |  |  |  |  |  |  |  | No definition, intervention description/ rationale only |
| Eyler (2016) |  |  |  |  | Y^d^ |  | Y^e^ | Y | Y |  | Y | Y^b^ |  |  | Y |  |  |  |  | Y | Y |  |  | “MI is a unique, patient-centered counseling style that focuses on facilitating changes in behavior by exploring and resolving ambivalence and resistance.1...Health care professionals can apply MI to assess a patient’s readiness to act on a specific behavior while using specific skills and strategies that respect patient autonomy and facilitate confidence and decision-making.2-4,8… core communication skills include: expressing empathy, supporting self-efficacy, avoiding argumentation, rolling with resistance, and developing discrepancy.2,3” (p38) |
| Drevenhorn (2012) | Y |  |  | Y | Y^d^ |  | Y^e^ |  | Y |  |  |  |  |  | Y |  |  |  |  | Y |  |  |  | “Counselling in a patient-centred way is designed to facilitate the active involvement of the patient to manage the illness” (Drevenhorn et al., 2007 p1)… MI is a non-confrontational way of calling attention to risky behaviours, which is important in order not to offend or scare away the patient but to create a comforting climate for a constructive discussion about the patient’s risk profile” (Drevenhorn et al., 2007 p47) |
| Cook (2017) | Y |  |  |  | Y |  | Y^e^ | Y |  |  | Y |  |  |  |  | Y | Y |  |  | Y |  |  |  | “Motivational interviewing (MI) is a patient-centered psychological counseling method…Key components of MI include a recognition that all people are ambivalent about change, a guiding and egalitarian style rather than a directive “expert” role, and the use of strategies like reflective listening and open-ended questions to draw out patients’ own statements about their motivators, challenges, and decisions related to health behaviors (Miller & Rollnick, 2013).” (p146) |
| Graham (2016) |  | Y | Y^a^ |  | Y |  |  | Y |  |  |  |  |  |  |  |  |  |  |  | Y |  |  |  | No definition, intervention description/ rationale only |
| Hedegaard (2015) |  |  |  |  | Y^d^ |  | Y^e^ |  |  |  |  | Y^b^ |  |  |  |  |  |  |  |  |  |  |  | No definition, intervention description/ rationale only |
| George (2020) |  | Y | Y^a^ |  | Y |  | Y^e^ | Y | Y |  | Y |  | Y | Y | Y | Y |  |  |  | Y |  |  |  | “MI is a patient‐centred counselling approach that elicits behavioural change by helping patients explore and resolve ambivalence towards change by engaging them in collaborative partnerships, focusing on connecting behaviour to outcomes, evoking the individual's internal motivation to change and planning a course of action (Miller & Rollnick, 2007).” (p877) |
| **Alcohol** |  |  |  |  |  |  |  |  |  |  |  |  |  |  |  |  |  |  |  |  |  |  |  |  |
| Bager (2010)^c^ |  |  |  | Y |  |  | Y^e^ |  |  |  |  |  |  |  |  |  |  |  |  |  |  |  |  | No definition, intervention description/ rationale only |
| Noknoy (2010) |  | Y |  | Y | Y |  | Y |  |  |  | Y | Y^b^ |  |  |  | Y | Y |  |  | Y |  |  |  | “Motivational Enhancement Therapy (MET) was originally developed as a brief four-session adaptation of Motivational Interviewing in Project MATCH (Miller et al., 1992). This approach uses patient-centred interviewing techniques to enhance patients’ motivation to change their drinking behaviour” (p2) |
| Aalto (2000) |  |  | Y^a^ | Y |  |  | Y | Y | Y |  |  | Y^b^ |  |  | Y | Y |  |  |  |  |  |  |  | “Brief intervention refers to any therapeutic or preventive consultation of short duration undertaken by a health care professional. In previous studies, brief interventions have included one to five sessions. In contrast to conventional alcoholism treatment, brief intervention is often performed by a healthcare worker who is not a specialist in addiction treatment. Generally, it takes place elsewhere than in an addiction treatment setting and the usual treatment goal is moderate drinking rather than total abstinence” (Aalto et al., 2001; p224). |
| Aalto (2001) |  |  | As above | As above |  |  | As above | As above | As above |  |  | As above |  |  | As above | As above |  |  |  |  |  |  |  |  |
| L'Engle (2014) |  |  | Y^a^ | Y | Y |  |  |  |  |  |  | Y^b^ |  |  |  |  |  |  |  |  |  |  |  | No definition, intervention description/ rationale only |
| Schaus (2009) | Y |  | Y^a^ |  | Y |  | Y |  |  |  |  |  |  |  |  |  | Y | Y |  | Y | Y |  |  | “BMIs combine cognitive-behavioral skills with personalized feedback, norms clarification, risk-reduction strategies, and motivational enhancement (p132)...The MI framework included clinician Empathizing, reflecting, reframing negative talk into change talk, rolling with resistance, avoiding argumentation, developing discrepancy between negative or ambivalent feelings toward alcohol, supporting self-efficacy through contemplation of past success, and acknowledging reluctance to change.” (p134) |
| Fleming (2010) | Y | Y | Y^a^ |  | Y |  |  |  |  |  | Y | Y |  |  |  | Y |  |  |  | Y |  | Y^f^ |  | No definition, intervention description/ rationale only |
| Dhital (2015) | Y | Y | Y | Y | Y |  |  |  | Y |  | Y |  | Y | Y | Y | Y |  |  |  | Y |  |  |  | BIs are discussions which seek to change views of the personal acceptability of excessive drinking and to encourage self-directed behaviour change. They include simple forms of structured advice and brief counsel-ling. Typically, questions about alcohol use are asked to motivate the person to take action to change drinking where this may be beneficial [4].” (Dhital et al., 2013; p1) |
| Ockene (1999) |  | Y | Y |  | Y |  |  | Y | Y |  | Y |  | Y |  | Y |  |  |  |  | Y |  |  |  | No definition, intervention description/ rationale only |
| Zatzick (2014) | Y | Y | Y^a^ | Y | Y |  | Y | Y | Y |  | Y | Y | Y | Y | Y | Y |  |  |  | Y |  |  |  | “MI approaches encourage movement in the direction of reductions in risky drinking behaviors by collaboratively promoting conversations that aim to strengthen an individual patient’s motivation and commitment to change [31].” (p756) |
| D'Onofrio (2008) | Y | Y | Y^a^ | Y | Y |  | Y^e^ | Y | Y |  | Y | Y | Y | Y | Y | Y | Y | Y |  | Y | Y |  |  | “Brief interventions are counseling sessions ranging from 10 to 45 minutes, typically performed by non-addiction specialists… that incorporate feedback, advice, and motivational enhancement techniques to assist the patient in reducing his or her alcohol consumption to low-risk guidelines, thereby reducing the risk of illness/injury.” (p743) |
| Shin (2013) |  | Y | Y | Y | Y^d^ |  | Y | Y | Y |  |  | Y^b^ |  | Y | Y | Y |  |  |  |  |  |  |  | “Motivational Interviewing (MI) represents a therapeutic style of counseling rather than a single intervention (Rollnick andMiller, 1995)... the MI style is not confrontational; instead, patient-centered yet directive counseling encourages collaboration and assists the client in exploring his or her own ambivalence about behavior change.” (Greenfield et al., p25) |
| **Sub-optimal Glycaemic Control** | | | | |  |  |  |  |  |  |  |  |  |  |  |  |  |  |  |  |  |  |  |  |
| Lauffenburger (2019) | Y | Y | Y^a^ | Y | Y |  |  | Y | Y |  | Y |  |  |  | Y | Y |  |  |  | Y |  |  |  | “Brief negotiated interviewing is a form of motivational interviewing that involves short counseling sessions of feedback, advice, and motivational enhancement techniques” (p2) |
| Juul (2014) |  | Y | Y |  | Y |  |  |  | Y |  | Y | Y^b^ |  |  | Y | Y |  |  |  | Y |  | Y |  | “The method Motivational interviewing (MI) has been assumed to be largely in accordance with SDT [27]” (p8) |
| Ismail (2018) | Y | Y | Y | Y | Y |  | Y | Y | Y |  | Y | Y | Y | Y | Y |  | Y | Y | Y | Y | Y |  |  | MI is a collaborative, person-centred approach to working with people in order to elicit and strengthen their motivation and commitment to change [19]. (Magill et al., 2018; p3) |
| **Multiple Health Behavior Change** | | | | |  |  |  |  |  |  |  |  |  |  |  |  |  |  |  |  |  |  |  |  |
| Christian (2011) |  | Y | Y^a^ | Y | Y |  |  |  |  |  |  | Y^b^ |  |  |  | Y |  |  |  |  |  |  |  | “MI techniques provide patients with an opportunity to take a more active role in the change process. Use of motivational inter-viewing techniques by clinicians focuses on resolving patient ambivalence as a method to enhance motivation for change (29). This approach uses patient resistance to give the provider information about readiness for change and is usually more satisfying to both the patient and the clinician. This brief intervention is based on principles of effective health behavior change interventions, and can be successfully employed in primary care patient encounters (54)” (p76) |
| Christian (2008) |  | Y | Y^a^ | Y | Y |  |  |  |  |  |  |  |  |  |  | Y |  |  |  |  |  |  |  | “Motivational interviewing techniques provide patients with an opportunity to take a more active role in the change process. Use of motivational interviewing techniques by clinicians encourages patients to reflect on and express their own motivation for change.32 This process reduces or sidesteps patient resistance and is usually more satisfying to both the patient and the clinician.” (p2) |
| Lakerveld (2013) |  | Y |  | Y | Y |  | Y | Y | Y |  |  | Y^b^ |  |  | Y |  | Y | Y |  | Y | Y |  |  | No definition, intervention description/ rationale only |
| Heinrich (2010) | Y | Y | Y | Y | Y |  | Y^e^ | Y | Y |  | Y | Y^b^ | Y | Y | Y |  | Y |  |  | Y | Y |  |  | No definition, intervention description/ rationale only |
| Whittemore (2009)^b^ |  | Y | Y^a^ |  | Y |  |  | Y |  |  |  |  |  |  |  |  |  |  |  |  |  |  |  | “MI is a collaborative counseling method for enhancing motivation to change by exploring and resolving ambivalence when individuals are having difficulty meeting mutually determined treatment goals (Rollnick, Miller, & Butler, 2007)” (p2) |
| Verweij (2012) |  | Y | Y |  | Y |  |  |  | Y |  |  |  |  |  | Y |  |  |  |  | Y |  |  |  | Behavioral change counseling is an adapted form of motivational interviewing, suitable for brief consultations in healthcare settings [53].” (p501) |
| Koelewijn-vanLoon (2010) |  | Y | Y | Y | Y |  | Y | Y | Y |  |  |  |  |  | Y |  |  | Y |  | Y | Y | Y |  | “Motivational interviewing is a directive, client-centred counselling style that helps clients explore and resolve ambivalence about behaviour change.29 Adapted motivational interviewing has been used to assess patients’ motivations for behaviour change and build motivation for healthy behaviours, to clarify values, to achieve goal setting and concrete action plans and to reveal both positive and negative consequences of behaviour change.30–32” (Koelewijn-vanLoon, 2009; E268) |
| Koelewijn-vanLoon (2009) |  | As above | As above | As above | As above |  | As above | As above | As above |  |  |  |  |  | As above |  |  | As above |  | As above | As above | As above |  |  |
| Nanchahal (2012) |  | Y | Y | Y | Y |  | Y | Y | Y |  |  | Y^b^ |  |  | Y |  |  |  |  | Y |  |  |  | “MI aims to support this by identifying and enhancing an individual’s own motivation and self-efficacy. The health professional employs an empathic, supportive and collaborative approach, emphasising the individual’s autonomy and encouraging the person to explore their own reasons for, and ambivalence about, changing the target behaviour [14]…Whilst motivational interviewing is effective in promoting behaviour change, many health professionals are ‘generalists’, using a variety of approaches rather than a single, ‘pure’ approach. ‘Motivational interviewing-style’ approaches…employ some of the elements (such as empathy) without using the full range of techniques [15].” (Noble et al., 2018; p2) |
| Butler (2013) | Y | Y | Y | Y | Y |  | Y^e^ | Y | Y |  | Y |  |  |  | Y | Y | Y |  |  | Y | Y |  |  | MI is defined as “a person-centered counselling style for addressing the common problem of ambivalence about change,”23 and refined over 15 years into a method for addressing the challenge of efficient, respectful, and effective consultations in primary care about multiplebe haviours.24”… BCC emphasises engaging the patient, and patient and health professional together choosing which lifestyle behaviours the patient might focus on. It recognised also that many people were likely to feel ambivalent about change, and would not necessarily respond well to straightforward advice. Instead, people would be supported with information to make their own decisions about why and how they might change.” (p2) |
| Jansink (2013) |  |  |  |  | Y |  | Y | Y | Y |  | Y | Y^b^ | Y |  | Y |  | Y | Y |  | Y | Y |  |  | “MI is formally defined as a patient centred, directive method for enhancing intrinsic motivation to change by exploring and resolving ambivalence [10]. Patient and professionals are jointly responsible for the treatment plan [11]. There are four general techniques of MI: (1) express empathy, (2) develop discrepancies, (3) roll with resistance, and (4) support self-efficacy…Five specific methods (open questions, affirming, reflecting, summarizing, and eliciting change talk) can be useful throughout the MI. Also agenda setting, scaling questions, and assessing importance and confidence in changing life-style can be used as techniques to support MI [12].” (p2) |
| Bóveda-Fontán (2015) |  |  |  |  | Y |  | Y^e^ | Y | Y |  |  |  |  |  | Y |  |  |  |  | Y |  |  |  | “Motivational Interviewing, as defined [3] is a clinical approach aimed at increasing patients' inherent motivation for change by helping them explore and solve their ambivalences and resistance to change from a patient-centered approach. In the authors' words, "MI is a collaborative goal-oriented method of communication with particular attention to the language of change" [4]. It involves the conscious and disciplined use of specific communication principles and strategies…”(p2) |
| Other Health Behaviors |  |  |  |  |  |  |  |  |  |  |  |  |  |  |  |  |  |  |  |  |  |  |  |  |
| Godard (2011) | Y | Y | Y | Y | Y |  | Y |  |  |  | Y |  | Y |  |  | Y | Y | Y |  | Y | Y |  |  | “MI is a client-centered directive method for enhancing intrinsic motivation to change by exploring and resolving ambivalence” (Miller & Rollnick2006)...The motivational interview has proven very effective in consultations in which ambivalence and motivation are central to the process of change. Open questions regarding patient support and value, and listening to, summarizing and restating sentences are key principles in motivational interview.” (p1100) |
| Dermen (2014) |  | Y | Y |  | Y |  |  |  | Y |  | Y |  |  | Y | Y | Y |  |  |  |  |  |  |  | “…motivational interviewing (MI; Rollnick, Miller, & Butler, 2008), in which patients choose their own goals for change (Freeman, 1999; Koerber, 2006).” (p392) |
| Cornman (2008) |  | Y |  | Y | Y |  | Y^e^ | Y | Y |  |  |  |  |  | Y |  |  |  |  | Y |  |  |  | “MI is an empirically validated, brief, patient-centered strategy for promoting risk behavior change in clinical settings.” (Fisher et al., 2006; p 45) |
| Hegarty (2013) |  |  |  | Y | Y |  | Y | Y | Y |  |  | Y^b^ |  |  | Y |  | Y | Y |  |  | Y |  |  | “Motivational interviewing (Miller & Rollnick 2002) is one option for health providers to apply in assisting women, tending to be most effective for those who are in contemplation (committed but questioning). The main features of motivational interviewing are to demonstrate support and empathy, create discrepancy between the individual's current and desired states, roll with resistance, avoid argumentation and build self­ efficacy.” (Hegarty et al., 2008; p 381) |
| Fisher (2014) |  | Y |  | Y | Y |  | Y^e^ | Y | Y |  |  |  |  |  | Y |  |  |  |  | Y |  |  |  | “MI is an empirically validated, brief, patient-centered strategy for promoting risk behavior change in clinical settings.” (Fisher et al., 2006; p 45) |
| Britton (2019) |  | Y | Y |  | Y |  | Y | Y | Y |  | Y |  |  | Y | Y |  |  | Y |  | Y |  | Y |  | “BCC is a collaborative, patient-centred approach that incorporates motivational interviewing and behavioural strategies. Motivational interviewing posits that elements of the therapeutic encounter (including empathy, collaboration, exploring ambivalence and eliciting reasons for change) are critical for strengthening commitment, thereby promoting momentum towards behaviour change and/or maintenance [5].” (Beck et al., 2017, p405) |

*Note*

*^‡^* ^BCC components have been defined according to the definition offered by Rollnick et al (2002)^

^a^ ‘Feedback’, ‘information’ or ‘education’ provided, without explicit mention of how interactive (i.e. exchange) this was; ^b^ Support self-efficacy, encouragement or reinforcement/ positive feedback; ^c^ MI or ‘MI techniques’ utilised, without explicit mention of specific skills; ^d^ Explore and resolve ambivalence without explicit mention of motivation; ^e^client/ patient centred, supportive, caring, non-confrontational/ non-stigmatising/ non-judgemental as opposed to explicitly referencing empathy; ^e^ Written summary; ^f^personal and affect-related motives/ life goals; ^†^MI informed intervention not defined, but BCC clearly cited
