# Supplementary material for: Adapted motivational interviewing for brief healthcare consultations: A systematic review and meta‐analysis of treatment fidelity in real‐world evaluations of behaviour change counselling
Source: Br J Health Psychol. 2023 May 4;28(4):972–99. doi: 10.1111/bjhp.12664 (PMC10947272; doi:10.1111/bjhp.12664)
Supplement: Supplementary file 12 — File S1 [file BJHP-28-972-s011.docx]

**Supplementary File 1**

**Included Articles**

| **Study** | **Citation Details** |
| --- | --- |
| **Substance Use** | |
| 1 | Mitcheson L, McCambridge J, Byrne S. Pilot cluster-randomised trial of adjunctive motivational interviewing to reduce crack cocaine use in clients on methadone maintenance. Eur Addict Res. 2007;13(1):6-10, doi:10.1159/000095809. |
| 2a^a^ | Gryczynski J, Mitchell SG, Gonzales A, Moseley A, Peterson TR, Ondersma SJ, et al. A randomized trial of computerized vs. in-person brief intervention for illicit drug use in primary care: Outcomes through 12months. J Subst Abuse Treat. 2015;50:3-10, doi:10.1016/j.jsat.2014.09.002. |
| 2b^a^ | Schwartz RP, Gryczynski J, Mitchell SG, Gonzales A, Moseley A, Peterson TR, et al. Computerized versus in-person brief intervention for drug misuse: A randomized clinical trial. Addiction. 2014;109(7):1091-8, doi:10.1111/add.12502 |
| 3 | Jaffray M, Matheson C, Bond CM, Lee AJ, McLernon DJ, Johnstone A, et al. Does training in motivational interviewing for community pharmacists improve outcomes for methadone patients? A cluster randomised controlled trial. Int J Pharm Pract. 2014;22(1):4-12, doi:10.1111/ijpp.12049. |
| 4a | Mertens JR, Ward CL, Bresick GF, Broder T, Weisner CM. Effectiveness of nurse-practitioner-delivered brief motivational intervention for young adult alcohol and drug use in primary care in South Africa: A randomized clinical trial. Alcohol Alcohol. 2014;49(4):430-8, doi:10.1093/alcalc/agu030. |
| 4b | Ward C, R. Mertens J, F. Bresick G, Little F, Weisner C. Screening and brief intervention for substance misuse: Does it reduce aggression and HIV-related risk behaviours?2015. |
| 5a | Garner BR, Gotham HJ, Chaple M, Martino S, Ford JH, Roosa MR, et al. The implementation and sustainment facilitation strategy improved implementation effectiveness and intervention effectiveness: Results from a cluster-randomized, type 2 hybrid trial. Implementation Research and Practice. 2020;1:2633489520948073, doi:10.1177/2633489520948073. |
| 5b | Garner BR, Gotham HJ, Tueller SJ, Ball EL, Kaiser D, Stilen P, et al. Testing the effectiveness of a motivational interviewing-based brief intervention for substance use as an adjunct to usual care in community-based AIDS service organizations: Study protocol for a multisite randomized controlled trial. Addict Sci Clin Pract. 2017;12(1):31, doi:10.1186/s13722-017-0095-8. |
| 5c | Garner BR, Gotham HJ, Tueller SJ, Ball EL, Kaiser D, Stilen P, et al. Testing the effectiveness of a motivational interviewing-based brief intervention for substance use as an adjunct to usual care in community-based AIDS service organizations: Study protocol for a multisite randomized controlled trial. Addict Sci Clin Pract. 2017;12(1):31, doi:10.1186/s13722-017-0095-8. |
| 6a | Darker CD, Sweeney B, Keenan E, Whiston L, Anderson R, Barry J. Screening and brief interventions for illicit drug use and alcohol use in methadone maintained opiate-dependent patients: Results of a pilot cluster randomized controlled trial feasibility study. Subst Use Misuse. 2016;51(9):1104-15, doi:10.3109/10826084.2016.1160118. |
| 6b | Darker C, Sweeney B, Keenan E, Whiston L, Anderson R, Barry J. Tailoring a brief intervention for illicit drug use and alcohol use in Irish methadone maintained opiate dependent patients: A qualitative process. BMC Psychiatry. 2016;16(1):373, doi:10.1186/s12888-016-1082-4. |
| **Physical Activity** | |
| 7 | Jackson R, Asimakopoulou K, Scammell A. Assessment of the transtheoretical model as used by dietitians in promoting physical activity in people with type 2 diabetes. J Hum Nutr Diet. 2007;20(1):27-36, doi:10.1111/j.1365-277X.2007.00746.x. |
| 8 | Elley CR, Kerse N, Arroll B, Robinson E. Effectiveness of counselling patients on physical activity in general practice: Cluster randomised controlled trial. BMJ. 2003;326(7393):793, doi:10.1136/bmj.326.7393.793. |
| 9 | Dennett AM, Shields N, Peiris CL, Prendergast LA, O'Halloran PD, Parente P, et al. Motivational interviewing added to oncology rehabilitation did not improve moderate-intensity physical activity in cancer survivors: A randomised trial. J Physiother. 2018;64(4):255-63, doi:10.1016/j.jphys.2018.08.003. |
| 10 | O'Halloran PD, Shields N, Blackstock F, Wintle E, Taylor NF. Motivational interviewing increases physical activity and self-efficacy in people living in the community after hip fracture: A randomized controlled trial. Clin Rehabil. 2016;30(11):1108-19, doi:10.1177/0269215515617814. |
| 11a | van der Weegen S, Verwey R, Spreeuwenberg M, Tange H, van der Weijden T, de Witte L. It's LiFe! Mobile and web-based monitoring and feedback tool embedded in primary care increases physical activity: A cluster randomized controlled trial. J Med Internet Res. 2015;17(7):e184, doi:10.2196/jmir.4579.es |
| 11b | Verwey R, van der Weegen S, Spreeuwenberg M, Tange H, van der Weijden T, de Witte L. Process evaluation of physical activity counselling with and without the use of mobile technology: A mixed methods study. Int J Nurs Stud. 2016;53:3-16, doi:10.1016/j.ijnurstu.2015.10.008. |
| 11c | Verwey R, van der Weegen S, Spreeuwenberg M, Tange H, van der Weijden T, de Witte L. A monitoring and feedback tool embedded in a counselling protocol to increase physical activity of patients with COPD or type 2 diabetes in primary care: Study protocol of a three-arm cluster randomised controlled trial. BMC Fam Pract. 2014;15(1):93, doi:10.1186/1471-2296-15-93. |
| 11d | Verwey R, van der Weegen S, Spreeuwenberg M, Tange H, van der Weijden T, de Witte L. Upgrading physical activity counselling in primary care in the Netherlands. Health Promotion International. 2016;31(2):344-54, doi:10.1093/heapro/dau107. |
| 11e | Verwey R, van der Weegen S, Spreeuwenberg M, Tange H, van der Weijden T, de Witte L. A pilot study of a tool to stimulate physical activity in patients with COPD or type 2 diabetes in primary care. J Telemed Telecare. 2014;20(1):29-34, doi:10.1177/1357633x13519057. |
| **Smoking** | |
| 12 | Louwagie GMC, Okuyemi KS, Ayo-Yusuf OA. Efficacy of brief motivational interviewing on smoking cessation at tuberculosis clinics in Tshwane, South Africa: A randomized controlled trial. Addiction. 2014;109(11):1942-52, doi:10.1111/add.12671. |
| 13a | Cabezas C, Advani M, Puente D, Rodriguez-Blanco T, Martin C. Effectiveness of a stepped primary care smoking cessation intervention: Cluster randomized clinical trial (ISTAPS study). Addiction. 2011;106(9):1696-706, doi:10.1111/j.1360-0443.2011.03491.x. |
| 13b | Cabezas C, Martin C, Granollers S, Morera C, Ballve JL, Zarza E, et al. Effectiveness of a stepped primary care smoking cessation intervention (ISTAPS study): Design of a cluster randomised trial. BMC Public Health. 2009;9(1):48, doi:10.1186/1471-2458-9-48. |
| 14 | Meyer C, Ulbricht S, Gross B, Kästel L, Wittrien S, Klein G, et al. Adoption, reach and effectiveness of computer-based, practitioner delivered and combined smoking interventions in general medical practices: A three-arm cluster randomized trial. Drug Alcohol Depend. 2012;121(1):124-32, doi:10.1016/j.drugalcdep.2011.08.019. |
| 15 | Cossette S, Frasure-Smith N, Robert M, Chouinard MC, Juneau M, Guertin MC, et al. A pilot randomized trial of a smoking cessation nursing intervention in cardiac patients after hospital discharge. Can J Cardiovasc Nurs. 2012;22(4):16-26. |
| 16 | Glasgow RE, Whitlock EP, Eakin EG, Lichtenstein E. A brief smoking cessation intervention for women in low-income planned parenthood clinics. Am J Public Health. 2000;90(5):786-9, doi:10.2105/ajph.90.5.786. |
| 17 | Ershoff DH, Quinn VP, Boyd NR, Stern J, Gregory M, Wirtschafter D. The Kaiser Permanente prenatal smoking-cessation trial: When more isn't better, what is enough? Am J Prev Med. 1999;17(3):161-8, doi:10.1016/s0749-3797(99)00071-9. |
| 18a | Butler CC, Rollnick S, Cohen D, Bachmann M, Russell I, Stott N. Motivational consulting versus brief advice for smokers in general practice: A randomized trial. The British Journal of General Practice. 1999;49(445):611-6. |
| 18b | Rollnick S, Butler CC, Stott N. Helping smokers make decisions: The enhancement of brief intervention for general medical practice. Patient Educ Couns. 1997;31(3):191-203, doi:10.1016/S0738-3991(97)01004-5. |
| 19 | Borrelli B, Novak S, Hecht J, Emmons K, Papandonatos G, Abrams D. Home health care nurses as a new channel for smoking cessation treatment: Outcomes from project CARES (Community-nurse Assisted Research and Education on Smoking). Prev Med. 2005;41(5):815-21, doi:10.1016/j.ypmed.2005.08.004. |
| 20 | Hollis JF, McAfee TA, Fellows JL, Zbikowski SM, Stark M, Riedlinger K. The effectiveness and cost effectiveness of telephone counselling and the nicotine patch in a state tobacco quitline. Tob Control. 2007;16 Suppl 1(Suppl 1):i53-i9, doi:10.1136/tc.2006.019794. |
| **Treatment Adherence/ Engagement** | |
| 21 | Leiva A, Aguilo A, Fajo-Pascual M, Moreno L, Martin MC, Garcia EM, et al. Efficacy of a brief multifactorial adherence-based intervention in reducing blood pressure: A randomized clinical trial. Patient Preference and Adherence. 2014;8:1683-90, doi:10.2147/PPA.S66927. |
| 22 | Eyler R, Shvets K, Blakely ML. Motivational interviewing to increase postdischarge antibiotic adherence in older adults with pneumonia. The Consultant Pharmacist. 2016;31(1):38-43, doi:10.4140/TCP.n.2016.38. |
| 23a^a^ | Drevenhorn E, Bengtson A, Nilsson PM, Nyberg P, Kjellgren KI. Consultation training of nurses for cardiovascular prevention – A randomized study of 2 years duration. Blood Press. 2012;21(5):293-9, doi:10.3109/08037051.2012.680734. |
| 23b^a^ | Drevenhorn E, Bengtson A, Nyberg P, Kjellgren KI. Assessment of hypertensive patients' self-care agency after counseling training of nurses. J Am Assoc Nurse Pract. 2015;27(11):624-30, doi:10.1002/2327-6924.12222. |
| 23c | Drevenhorn E, Bengtson A, Allen J, Säljö R, Kjellgren K. A content analysis of patient centredness in hypertension care after consultation training for nurses. The Internet Journal of Advanced Nursing Practice. 2007;8(2). |
| 23d | Drevenhorn E, Bengtson A, K Allen J, Säljö R, Kjellgren K. Counselling on lifestyle factors in hypertension care after training on the stages of change model2007. 46-53 p. |
| 24 | Cook PF, Schmiege SJ, Mansberger SL, Sheppler C, Kammer J, Fitzgerald T, et al. Motivational interviewing or reminders for glaucoma medication adherence: Results of a multi-site randomised controlled trial. Psychol Health. 2017;32(2):145-65, doi:10.1080/08870446.2016.1244537. |
| 25 | Graham HL, Copello A, Griffith E, Freemantle N, McCrone P, Clarke L, et al. Pilot randomised trial of a brief intervention for comorbid substance misuse in psychiatric in-patient settings. Acta Psychiatr Scand. 2016;133(4):298-309, doi:10.1111/acps.12530. |
| 26a | Hedegaard U, Kjeldsen LJ, Pottegard A, Henriksen JE, Lambrechtsen J, Hangaard J, et al. Improving medication adherence in patients with hypertension: A randomized trial. Am J Med. 2015;128(12):1351-61, doi:10.1016/j.amjmed.2015.08.011. |
| 26b | Hedegaard U, Hallas J, Ravn-Nielsen LV, Kjeldsen LJ. Process- and patient-reported outcomes of a multifaceted medication adherence intervention for hypertensive patients in secondary care. Res Social Adm Pharm. 2016;12(2):302-18, doi:10.1016/j.sapharm.2015.05.006. |
| 27a | George M, Bruzzese J-M, S. Sommers M, Pantalon MV, Jia H, Rhodes J, et al. Group-randomized trial of tailored brief shared decision-making to improve asthma control in urban black adults. Journal of Advanced Nursing. 2021;77(3):1501-17, doi:https://doi.org/10.1111/jan.14646. |
| 27b | George M, Pantalon MV, Sommers MLS, Glanz K, Jia H, Chung A, et al. Shared decision-making in the BREATHE asthma intervention trial: A research protocol. J Adv Nurs. 2019;75(4):876-87, doi:10.1111/jan.13916. |
| 27c | George M, Arcia A, Chung A, Coleman D, Bruzzese J-M. African Americans want a focus on shared decision-making in asthma adherence interventions. Patient. 2020;13(1):71-81, doi:10.1007/s40271-019-00382-x. |
| **Alcohol** | |
| 28 | Bager P, Vilstrup H. Post-discharge brief intervention increases the frequency of alcohol abstinence—A randomized trial. J Addict Nurs. 2010;21(1):37–41, doi:10.3109/10884601003628104. |
| 29 | Noknoy S, Rangsin R, Saengcharnchai P, Tantibhaedhyangkul U, McCambridge J. RCT of effectiveness of motivational enhancement therapy delivered by nurses for hazardous drinkers in primary care units in Thailand. Alcohol Alcohol. 2010;45(3):263-70, doi:10.1093/alcalc/agq013. |
| 30a^a^ | Aalto M, Saksanen R, Laine P, Forsström R, Raikaa M, Kiviluoto M, et al. Brief intervention for female heavy drinkers in routine general practice: A 3-year randomized, controlled study. Alcohol Clin Exp Res. 2000;24(11):1680-6, doi:10.1097/00000374-200011000-00010. |
| 30b^a^ | Aalto M, Seppä K, Mattila P, Mustonen H, Ruuth K, Hyvärinen H, et al. Brief intervention for male heavy drinkers in routine general practice: A three-year randomized controlled study. Alcohol Alcohol. 2001;36(3):224-30, doi:10.1093/alcalc/36.3.224. |
| 31 | L'Engle K, Mwarogo P, Kingola N, Sinkele W, Min D, H Weiner D. A randomized controlled trial of a brief intervention to reduce alcohol use among female sex workers in Mombasa, Kenya2014. |
| 32 | Schaus JF, Sole ML, McCoy TP, Mullett N, O'Brien MC. Alcohol screening and brief intervention in a college student health center: A randomized controlled trial. Journal of studies on alcohol and drugs Supplement. 2009(16):131-41, doi:10.15288/jsads.2009.s16.131. |
| 33 | Fleming MF, Balousek SL, Grossberg PM, Mundt MP, Brown D, Wiegel JR, et al. Brief physician advice for heavy drinking college students: A randomized controlled trial in college health clinics. Journal of Studies on Alcohol and Drugs. 2010;71(1):23-31, doi:10.15288/jsad.2010.71.23 |
| 34a | Dhital R, Norman I, Whittlesea C, Murrells T, McCambridge J. The effectiveness of brief alcohol interventions delivered by community pharmacists: Randomized controlled trial. Addiction. 2015;110(10):1586-94, doi:10.1111/add.12994. |
| 34b | Dhital R, Norman I, Whittlesea C, McCambridge J. Effectiveness of alcohol brief intervention delivered by community pharmacists: Study protocol of a two-arm randomised controlled trial. BMC Public Health. 2013;13:152, doi:10.1186/1471-2458-13-152. |
| 35a | Ockene JK, Adams A, Hurley TG, Wheeler EV, Hebert JR. Brief physician- and nurse practitioner–delivered counseling for high-risk drinkers: Does it work? Arch Intern Med. 1999;159(18):2198-205, doi:10.1001/archinte.159.18.2198. |
| 35b | Adams A, Ockene JK, Wheller EV, Hurley TG. Alcohol counseling: Physicians will do it. J Gen Intern Med. 1998;13(10):692-8, doi:10.1046/j.1525-1497.1998.00206.x. |
| 35c | Ockene JK, Wheeler EV, Adams A, Hurley TG, Hebert J. Provider training for patient-centered alcohol counseling in a primary care setting. Arch Intern Med. 1997;157(20):2334-41. |
| 36a | Zatzick D, Donovan DM, Jurkovich G, Gentilello L, Dunn C, Russo J, et al. Disseminating alcohol screening and brief intervention at trauma centers: A policy-relevant cluster randomized effectiveness trial. Addiction. 2014;109(5):754-65, doi:10.1111/add.12492. |
| 36b | Darnell DP, Dunn CP, Atkins DP, Ingraham LBS, Zatzick DMD. A randomized evaluation of motivational interviewing training for mandated implementation of alcohol screening and brief intervention in trauma centers. J Subst Abuse Treat. 2016;60:36, doi:10.1016/j.jsat.2015.05.010. |
| 36c | Zatzick DF, Donovan DM, Dunn C, Jurkovich GJ, Wang J, Russo J, et al. Disseminating organizational screening and brief intervention services (DO-SBIS) for alcohol at trauma centers study design. Gen Hosp Psychiatry. 2013;35(2):174-80, doi:10.1016/j.genhosppsych.2012.11.012. |
| 36d | Dunn C. Brief motivational interviewing interventions targeting substance abuse in the acute care medical setting. Semin Clin Neuropsychiatry. 2003;8(3):188-96, doi:10.1016/s1084-3612(03)00025-x. |
| 37a | D'Onofrio G, Pantalon MV, Degutis LC, Fiellin DA, Busch SH, Chawarski MC, et al. Brief intervention for hazardous and harmful drinkers in the emergency department. Annals of Emergency Medicine. 2008;51(6):742-50.e2, doi:10.1016/j.annemergmed.2007.11.028. |
| 37b | D'Onofrio G, Pantalon MV, Degutis LC, Fiellin DA, O'Connor P G. Development and implementation of an emergency practitioner-performed brief intervention for hazardous and harmful drinkers in the emergency department. Acad Emerg Med. 2005;12(3):249-56, doi:10.1197/j.aem.2004.10.021. |
| 38a | Shin S, Livchits V, Connery HS, Shields A, Yanov S, Yanova G, et al. Effectiveness of alcohol treatment interventions integrated into routine tuberculosis care in Tomsk, Russia. Addiction (Abingdon, England). 2013;108(8):1387-96, doi:10.1111/add.12148. |
| 38b | Greenfield SF, Shields A, Connery HS, Livchits V, Yanov SA, Lastimoso CS, et al. Integrated management of physician-delivered alcohol care for tuberculosis patients: Design and implementation. Alcohol Clin Exp Res. 2010;34(2):317-30, doi:10.1111/j.1530-0277.2009.01094.x. |
| 38c | Shin SS, Livchits V, Nelson AK, Lastimoso CS, Yanova GV, Yanov SA, et al. Implementing evidence-based alcohol interventions in a resource-limited setting: Novel delivery strategies in Tomsk, Russia. Harv Rev Psychiatry. 2012;20(1):58-67, doi:10.3109/10673229.2012.649121. |
| **Sub-optimal Glycaemic Control** | |
| 39a | Lauffenburger JC, Ghazinouri R, Jan S, Makanji S, Ferro CA, Lewey J, et al. Impact of a novel pharmacist-delivered behavioral intervention for patients with poorly-controlled diabetes: The ENhancing outcomes through Goal Assessment and Generating Engagement in Diabetes Mellitus (ENGAGE-DM) pragmatic randomized trial. PLoS One. 2019;14(4):e0214754, doi:10.1371/journal.pone.0214754. |
| 39b | Lauffenburger JC, Lewey J, Jan S, Nanchanatt G, Makanji S, Ferro CA, et al. Rationale and design of the ENhancing outcomes through Goal Assessment and Generating Engagement in Diabetes Mellitus (ENGAGE-DM) pragmatic trial. Contemp Clin Trials. 2017;59:57-63, doi:10.1016/j.cct.2017.05.014. |
| 40a | Juul L, Maindal HT, Zoffmann V, Frydenberg M, Sandbaek A. Effectiveness of a training course for general practice nurses in motivation support in type 2 diabetes care: A cluster-randomised trial. PLoS ONE [Electronic Resource]. 2014;9(5):e96683, doi:10.1371/journal.pone.0096683. |
| 40b | Juul L, Maindal HT, Zoffmann V, Frydenberg M, Sandbaek A. A cluster randomised pragmatic trial applying Self-determination theory to type 2 diabetes care in general practice. BMC Fam Pract. 2011;12(1):130, doi:10.1186/1471-2296-12-130. |
| 41a | Ismail K, Winkley K, de Zoysa N, Patel A, Heslin M, Graves H, et al. Nurse-led psychological intervention for type 2 diabetes: A cluster randomised controlled trial (Diabetes-6 study) in primary care. Br J Gen Pract. 2018;68(673):e531-e40, doi:10.3399/bjgp18X696185. |
| 41b | Magill N, Graves H, de Zoysa N, Winkley K, Amiel S, Shuttlewood E, et al. Assessing treatment fidelity and contamination in a cluster randomised controlled trial of motivational interviewing and cognitive behavioural therapy skills in type 2 diabetes. BMC Fam Pract. 2018;19(1):60, doi:10.1186/s12875-018-0742-5. |
| **Multiple Health Behavior Change** | |
| 42 | Christian JG, Byers TE, Christian KK, Goldstein MG, Bock BC, Prioreschi B, et al. A computer support program that helps clinicians provide patients with metabolic syndrome tailored counseling to promote weight loss. J Am Diet Assoc. 2011;111(1):75-83, doi:10.1016/j.jada.2010.10.006. |
| 43 | Christian JG, Bessesen DH, Byers TE, Christian KK, Goldstein MG, Bock BC. Clinic-based support to help overweight patients with type 2 diabetes increase physical activity and lose weight. Arch Intern Med. 2008;168(2):141-6, doi:10.1001/archinternmed.2007.13. |
| 44a | Lakerveld J, Bot SD, Chinapaw MJ, van Tulder MW, Kostense PJ, Dekker JM, et al. Motivational interviewing and problem solving treatment to reduce type 2 diabetes and cardiovascular disease risk in real life: A randomized controlled trial. International Journal of Behavioral Nutrition and Physical Activity. 2013;10(1):47, doi:10.1186/1479-5868-10-47. |
| 44b | van Wier MF, Lakerveld J, Bot SDM, Chinapaw MJM, Nijpels G, van Tulder MW. Economic evaluation of a lifestyle intervention in primary care to prevent type 2 diabetes mellitus and cardiovascular diseases: A randomized controlled trial. BMC Fam Pract. 2013;14(1):45, doi:10.1186/1471-2296-14-45. |
| 44c | Lakerveld J, Bot S, Chinapaw M, van Tulder M, Kingo L, Nijpels G. Process evaluation of a lifestyle intervention to prevent diabetes and cardiovascular diseases in primary care. Health Promotion Practice. 2012;13(5):696-706, doi:10.1177/1524839912437366. |
| 44d | Lakerveld J, Bot SD, Chinapaw MJ, van Tulder MW, van Oppen P, Dekker JM, et al. Primary prevention of diabetes mellitus type 2 and cardiovascular diseases using a cognitive behavior program aimed at lifestyle changes in people at risk: Design of a randomized controlled trial. BMC Endocr Disord. 2008;8:6, doi:10.1186/1472-6823-8-6. |
| 45 | Heinrich E, Candel MJJM, Schaper NC, de Vries NK. Effect evaluation of a motivational interviewing based counselling strategy in diabetes care. Diabetes Res Clin Pract. 2010;90(3):270-8, doi:10.1016/j.diabres.2010.09.012. |
| 46 | Whittemore R, Melkus G, Wagner J, Dziura J, Northrup V, Grey M. Translating the diabetes prevention program to primary care: A pilot study. Nurs Res. 2009;58(1):2-12, doi:10.1097/NNR.0b013e31818fcef3. |
| 47a | Verweij LM, Proper KI, Weel ANH, Hulshof CTJ, van Mechelen W. The application of an occupational health guideline reduces sedentary behaviour and increases fruit intake at work: results from an RCT. Occupational and Environmental Medicine. 2012;69(7):500, doi:10.1136/oemed-2011-100377. |
| 47b | Verweij LM, Proper KI, Weel AN, Hulshof CT, van Mechelen W. Design of the Balance@Work project: systematic development, evaluation and implementation of an occupational health guideline aimed at the prevention of weight gain among employees. BMC Public Health. 2009;9:461, doi:10.1186/1471-2458-9-461. |
| 47c | Verweij LM, Proper KI, Hulshof CT, van Mechelen W. Process evaluation of an occupational health guideline aimed at preventing weight gain among employees. J Occup Environ Med. 2011;53(7):722-9, doi:10.1097/JOM.0b013e318222af9b. |
| 48a | Koelewijn-van Loon MS, van der Weijden T, Ronda G, van Steenkiste B, Winkens B, Elwyn G, et al. Improving lifestyle and risk perception through patient involvement in nurse-led cardiovascular risk management: A cluster-randomized controlled trial in primary care. Prev Med. 2010;50(1-2):35-44, doi:10.1016/j.ypmed.2009.11.007. |
| 48b | Koelewijn-van Loon MS, van der Weijden T, van Steenkiste B, Ronda G, Winkens B, Severens JL, et al. Involving patients in cardiovascular risk management with nurse-led clinics: A cluster randomized controlled trial. CMAJ Canadian Medical Association Journal. 2009;181(12):E267-74, doi:10.1503/cmaj.081591. |
| 48c | Koelewijn-van Loon MS, van Steenkiste B, Ronda G, Wensing M, Stoffers HE, Elwyn G, et al. Improving patient adherence to lifestyle advice (IMPALA): A cluster-randomised controlled trial on the implementation of a nurse-led intervention for cardiovascular risk management in primary care (protocol). BMC Health Services Research. 2008;8:9, doi:10.1186/1472-6963-8-9. |
| 49a | Nanchahal K, Power T, Holdsworth E, Hession M, Sorhaindo A, Griffiths U, et al. A pragmatic randomised controlled trial in primary care of the Camden Weight Loss (CAMWEL) programme. BMJ Open. 2012;2(3):e000793, doi:10.1136/bmjopen-2011-000793. |
| 49b | Noble LM, Godfrey E, Al-Baba L, Baez G, Thorogood N, Nanchahal K. Treatment fidelity in the Camden Weight Loss (CAMWEL) intervention assessed from recordings of advisor-participant consultations. BMC Obes. 2018;5:24, doi:10.1186/s40608-018-0203-7. |
| 50a | Butler CC, Simpson SA, Hood K, Cohen D, Pickles T, Spanou C, et al. Training practitioners to deliver opportunistic multiple behaviour change counselling in primary care: A cluster randomised trial. BMJ. 2013;346:f1191, doi:10.1136/bmj.f1191. |
| 50b | Spanou C, Simpson SA, Hood K, Edwards A, Cohen D, Rollnick S, et al. Preventing disease through opportunistic, rapid engagement by primary care teams using behaviour change counselling (PRE-EMPT): Protocol for a general practice-based cluster randomised trial. BMC Fam Pract. 2010;11:69, doi:10.1186/1471-2296-11-69. |
| 51a | Jansink R, Braspenning J, Keizer E, van der Weijden T, Elwyn G, Grol R. No identifiable Hb1Ac or lifestyle change after a comprehensive diabetes programme including motivational interviewing: A cluster randomised trial. Scand J Prim Health Care. 2013;31(2):119-27, doi:10.3109/02813432.2013.797178. |
| 51b | Jansink R, Braspenning J, van der Weijden T, Niessen L, Elwyn G, Grol R. Nurse-led motivational interviewing to change the lifestyle of patients with type 2 diabetes (MILD-project): Protocol for a cluster, randomized, controlled trial on implementing lifestyle recommendations. BMC Health Services Research. 2009;9:19, doi:10.1186/1472-6963-9-19. |
| 51c | Jansink R, Braspenning J, Laurant M, Keizer E, Elwyn G, Weijden Tvd, et al. Minimal improvement of nurses’ motivational interviewing skills in routine diabetes care one year after training: A cluster randomized trial. BMC Fam Pract. 2013;14(44), doi:10.1186/1471-2296-14-44. |
| 52a | Bóveda-Fontán J, Barragán-Brun N, Campiñez-Navarro M, Pérula-de Torres LÁ, Bosch-Fontcuberta JM, Martín-Álvarez R, et al. Effectiveness of motivational interviewing in patients with dyslipidemia: A randomized cluster trial. BMC Fam Pract. 2015;16:1-16, doi:10.1186/s12875-015-0370-2. |
| 52b | Pérula LA, Bosch JM, Bóveda J, Campiñez M, Barragán N, Arboniés JC, et al. Effectiveness of Motivational Interviewing in improving lipid level in patients with dyslipidemia assisted by general practitioners: Dislip-EM study protocol. BMC Fam Pract. 2011;12:125, doi:10.1186/1471-2296-12-125. |
| 52c | Pérula LÁ, Campiñez M, Bosch JM, Brun NB, Arboniés JC, Fontán JB, et al. Is the Scale for Measuring Motivational Interviewing Skills a valid and reliable instrument for measuring the primary care professionals motivational skills?: EVEM study protocol. BMC Fam Pract. 2012;13:112, doi:10.1186/1471-2296-13-112. |
| **Other Health Behaviors** | |
| 53 | Godard A, Dufour T, Jeanne S. Application of self-regulation theory and motivational interview for improving oral hygiene: A randomized controlled trial. J Clin Periodontol. 2011;38(12):1099-105, doi:10.1111/j.1600-051X.2011.01782.x. |
| 54 | Dermen KH, Ciancio SG, Fabiano JA. A pilot test of motivational oral health promotion with alcohol-dependent inpatients. Health Psychol. 2014;33(4):392-5, doi:10.1037/a0033153. |
| 55 | Cornman DH, Kiene SM, Christie S, Fisher WA, Shuper PA, Pillay S, et al. Clinic-based intervention reduces unprotected sexual behavior among HIV-infected patients in KwaZulu-Natal, South Africa: Results of a pilot study. Journal of acquired immune deficiency syndromes (1999). 2008;48(5):553-60, doi:10.1097/QAI.0b013e31817bebd7. |
| 56a | Hegarty K, O'Doherty L, Taft A, Chondros P, Brown S, Valpied J, et al. Screening and counselling in the primary care setting for women who have experienced intimate partner violence (WEAVE): A cluster randomised controlled trial. The Lancet. 2013;382(9888):249-58, doi:10.1016/S0140-6736(13)60052-5. |
| 56b | Hegarty KL, Gunn JM, O'Doherty LJ, Taft A, Chondros P, Feder G, et al. Women's evaluation of abuse and violence care in general practice: A cluster randomised controlled trial (weave). BMC Public Health. 2010;10(1):2, doi:10.1186/1471-2458-10-2. |
| 56c | Hegarty KL, O’Doherty LJ, Gunn J, Pierce D, Taft AJ. A brief counselling intervention by health professionals utilising the ‘readiness to change’ concept for women experiencing intimate partner abuse: The weave project. Journal of Family Studies. 2008;14(2-3):376-88, doi:10.5172/jfs.327.14.2-3.376. |
| 57a | Fisher JDP, Cornman DHP, Shuper PAP, Christie SMPH, Pillay SM, Macdonald SBA, et al. HIV prevention counseling intervention delivered during routine clinical care reduces HIV risk behavior in HIV-infected South Africans receiving antiretroviral therapy: The Izindlela Zokuphila/Options for Health randomized trial. J Acquir Immune Defic Syndr. 2014;67(5):499, doi:10.1097/QAI.0000000000000348. |
| 57b | Fisher JD, Cornman DH, Osborn CY, Amico KR, Fisher WA, Friedland GA. Clinician-initiated HIV risk reduction intervention for HIV-positive persons: Formative research, acceptability, and fidelity of the Options Project. J Acquir Immune Defic Syndr. 2004;37 Suppl 2:S78-87, doi:10.1097/01.qai.0000140605.51640.5c. |
| 57c | Fisher JD, Fisher WA, Cornman DH, Amico RK, Bryan A, Friedland GH. Clinician-delivered intervention during routine clinical care reduces unprotected sexual behavior among HIV-infected patients. J Acquir Immune Defic Syndr. 2006;41(1):44-52, doi:10.1097/01.qai.0000192000.15777.5c. |
| 57d | Cornman DH, Christie S, Shepherd LM, MacDonald S, Amico KR, Smith LR, et al. Counsellor-delivered HIV risk reduction intervention addresses safer sex barriers of people living with HIV in KwaZulu-Natal, South Africa. Psychol Health. 2011;26(12):1623-41, doi:10.1080/08870446.2011.552180. |
| 58a | Britton B, Baker A, Wolfenden L, Wratten C, Bauer J, Beck AK, et al. Eating As Treatment (EAT): A stepped-wedge, randomised controlled trial of a health behaviour change intervention provided by dietitians to improve nutrition in patients with head and neck cancer undergoing radiotherapy. Int J Radiat Oncol Biol Phys. 2019;103(2):353-62, doi:10.1016/j.ijrobp.2018.09.027. |
| 58b | Britton B, McCarter K, Baker A, Wolfenden L, Wratten C, Bauer J, et al. Eating As Treatment (EAT) study protocol: A stepped-wedge, randomised controlled trial of a health behaviour change intervention provided by dietitians to improve nutrition in patients with head and neck cancer undergoing radiotherapy. BMJ Open. 2015;5(7), doi:10.1136/bmjopen-2015-008921. |
| 58c | Beck AK, Baker A, Britton B, Wratten C, Bauer J, Wolfenden L, et al. Fidelity considerations in translational research: Eating As Treatment - a stepped wedge, randomised controlled trial of a dietitian delivered behaviour change counselling intervention for head and neck cancer patients undergoing radiotherapy. Trials. 2015;16:465, doi:10.1186/s13063-015-0978-5. |
| 58d | Beck AK, Britton B, Baker A, Odelli C, Wratten C, Bauer J, et al. Preliminary report: Training head and neck cancer dietitians in behaviour change counselling. Psycho-Oncol. 2017;26(3):405-7, doi:10.1002/pon.4129. |
| 58e | Beck AK, Baker AL, Carter G, Wratten C, Bauer J, Wolfenden L, et al. Assessing adherence, competence and differentiation in a stepped-wedge randomised clinical trial of a complex behaviour change intervention. Nutrients. 2020;12(8), doi:10.3390/nu12082332. |

*Note*. Studies are presented in the same order as the summary of study characteristics (Table 1). Where eligible studies are described across multiple manuscripts, patient outcome papers are presented first. ^a^Patient outcomes are presented across multiple outcome papers
