# Supplementary material for: Adapted motivational interviewing for brief healthcare consultations: A systematic review and meta‐analysis of treatment fidelity in real‐world evaluations of behaviour change counselling
Source: Br J Health Psychol. 2023 May 4;28(4):972–99. doi: 10.1111/bjhp.12664 (PMC10947272; doi:10.1111/bjhp.12664)
Supplement: Supplementary file 13 — File S2 [file BJHP-28-972-s007.docx]

**Supplementary File 2**

**MEDLINE Search Strategy**

|  | ab,kw,sh,ti,tw |
| --- | --- |
| 1 | "behavior change counseling" |
| 2 | "behaviour change counseling" |
| 3 | "behavior change counselling" |
| 4 | "behavior change counseling" |
|  | ab,kw,sh,ti |
| 5 | "brief intervention" |
| 6 | "motivational interviewing" |
| 7 | 5 AND 6 |
| 8 | **1 OR 2 OR 3 OR 4 OR 6 OR 7** |
|  | ab,kw,sh,ti |
| 9 | "health care practitioner" |
| 10 | "healthcare practitioner" |
| 11 | "health care worker" |
| 12 | "healthcare worker" |
| 13 | counselor |
| 14 | counsellor |
| 15 | therapist |
| 16 | clinician |
| 17 | practitioner |
| 18 | specialist |
| 19 | technician |
| 20 | psychologist |
| 21 | "social worker" |
| 22 | chiropractor |
| 23 | dentist |
| 24 | dietitian |
| 25 | nutritionist |
| 26 | optometrist |
| 27 | pharmacist |
| 28 | Physician |
| 29 | doctor |
| 30 | "general practitioner" |
| 31 | surgeon |
| 32 | "occupational therapist" |
| 33 | "physical therapist" |
| 34 | "radiation therapist" |
| 35 | "recreational therapist" |
| 36 | "respiratory therapist" |
| 37 | "speech therapist" |
| 38 | "exercise physiologist" |
| 39 | nurse |
| 40 | anesthetist |
| 41 | midwive |
| 42 | audiologist |
| 43 | Anesthesiologist |
| 44 | Cardiologist |
| 45 | Dermatologist |
| 46 | Neurologist |
| 47 | Psychiatrist |
| 48 | Radiologist |
| 49 | 9 OR 10 OR 11 OR 12 OR 13 OR 14 OR 15 OR 16 OR 17 OR 18 OR 19 OR 20 OR 21 OR 22 OR 23 OR 24 OR 25 OR 26 OR 27 OR 28 OR 29 OR 30 OR 31 OR 32 OR 33 OR 34 OR 35 OR 36 OR 37 OR 38 OR 39 OR 40 OR 41 OR 42 OR 43 OR 44 OR 45 OR 46 OR 47 OR 48 |
| 50 | **8 AND 49** |
|  | ab,kw,sh,ti |
| 51 | "real world" |
| 52 | "real-world" |
| 53 | pragmatic |
| 54 | effectiveness |
| 55 | hybrid |
| 56 | "clinical care" |
| 57 | "secondary care" |
| 58 | "nursing home" |
| 59 | clinic |
| 60 | practice |
| 61 | "health center" |
| 62 | "health centre" |
| 63 | hospital |
| 64 | "emergency room" |
| 65 | "emergency department" |
| 66 | outpatient |
| 67 | rehabilitation |
| 68 | "employee assistance" |
| 69 | "Primary Healthcare" |
| 70 | "Primary health" |
| 71 | "general practice" |
| 72 | inpatient |
| 73 | "emergency health services" |
| 74 | "outpatient care" |
| 75 | 51 OR 52 OR 53 OR 54 OR 55 OR 56 OR 57 OR 58 OR 59 OR 60 OR 61 OR 62 OR 63 OR 64 OR 65 OR 66 OR 67 OR 68 OR 69 OR 70 OR 71 OR 72 OR 73 OR 74 |
| **76** | **50 AND 75** |
|  | Limit to humans and (adaptive clinical trial or clinical conference or clinical study or clinical trial, all or clinical trial or comparative study or controlled clinical trial or evaluation studies or government publications or pragmatic clinical trial or randomized controlled trial or technical report)) |
