# Supplementary material for: Adapted motivational interviewing for brief healthcare consultations: A systematic review and meta‐analysis of treatment fidelity in real‐world evaluations of behaviour change counselling
Source: Br J Health Psychol. 2023 May 4;28(4):972–99. doi: 10.1111/bjhp.12664 (PMC10947272; doi:10.1111/bjhp.12664)
Supplement: Supplementary file 14 — File S3 [file BJHP-28-972-s015.docx]

**Supplementary File 3**

**Change in Adherence (%) to the NIH Framework over Time**

**Analysis**

Articles were grouped according to three time periods: before the first version of the NIH checklist was published (< 2005); after it was published and before it was updated (2006 – 2011) and after the revised version was published (>2011). As date was inconsistently defined (i.e. variously provided for trial registration, funding, site recruitment, participant recruitment, participant enrolment and/ or study conduct), publications were grouped according to the earliest date reported. Where no date was reported, the year of publication was used. Linear regression was used to analyse the relationship between study year (< 2005 vs 2006-2011 and >2011) as the predictor variable and article adherence (%) to NIH fidelity recommendations (overall, and for each of the five fidelity domains) as separate dependent variables.

**Findings**

Eighteen studies (31%) were allocated to the group from before the NIH Checklist was first published((Aalto et al., 2000; Aalto et al., 2001; Bager & Vilstrup, 2010; Borrelli et al., 2005b; Butler et al., 1999; Cabezas et al., 2011; Christian et al., 2008; Cornman et al., 2008; D'Onofrio et al., 2008; Drevenhorn et al., 2015; Elley et al., 2003; Ershoff et al., 1999; Glasgow et al., 2000; Hollis et al., 2007; Koelewijn-van Loon et al., 2010; Koelewijn-van Loon et al., 2009; Meyer et al., 2012; Noknoy et al., 2010; Ockene et al., 1999; Schaus et al., 2009)); 28 (48%) between 2006 and 2011 ((Bóveda-Fontán et al., 2015; Butler et al., 2013; Christian et al., 2011; Cook et al., 2017; Cossette et al., 2012; Dermen et al., 2014; Fisher et al., 2014; Fleming et al., 2010; Godard et al., 2011; Gryczynski et al., 2015; Hegarty et al., 2013; Heinrich et al., 2010; Ismail et al., 2018; Jackson et al., 2007; Jaffray et al., 2014; R. Jansink et al., 2013; Juul et al., 2014; L'Engle et al., 2014; Lakerveld et al., 2013; Leiva et al., 2014; Louwagie et al., 2014; Mertens et al., 2014; Mitcheson et al., 2007; Nanchahal et al., 2012; Schwartz et al., 2014; Shin et al., 2013; Verweij et al., 2012; Whitehead et al., 2009; Zatzick et al., 2014) and 12 (20%) after the revised version of the NIH checklist was published (Britton et al., 2019; Darker et al., 2016a; Dennett et al., 2018; Dhital et al., 2015; Eyler et al., 2016; Garner et al., 2020; George et al., 2021; Graham et al., 2016; Hedegaard et al., 2016; Lauffenburger et al., 2019; O'Halloran et al., 2016; van der Weegen et al., 2015). Fidelity checklist score according to fidelity domain and study year is presented in Supplementary Figure 2. Attention to fidelity recommendations improved across time (*R*^2^=.074, *F*(1, 56)=4.46, p=0.039). Studies that commenced after the revision of the NIH Fidelity recommendations (>2011) demonstrated greater adherence (%) to the NIH checklist (*M*=71.82, *SD*=14.53) compared to those from before (*M*=58.95, *SD*=15.93; *t*(25.21)=2.28, *p*=0.016) and after (*M=*62.44, *SD=*16.26; *t*(23.24)=1.8, *p*=0.042) the first version of the NIH recommendations were published. Within individual fidelity domains, training (*R*^2^=.073, *F*(1,56)=4.379, *p*=0.041) and enactment (*R*^2^=.081, *F*(1,56)=4.95, *p*=0.030), but not design (*p*=1.41), delivery (*p*=0.056) or receipt (*p*=0.671) demonstrated a similar pattern of improvement across time (Supplementary Figure 8).
