# Supplementary material for: Adapted motivational interviewing for brief healthcare consultations: A systematic review and meta‐analysis of treatment fidelity in real‐world evaluations of behaviour change counselling
Source: Br J Health Psychol. 2023 May 4;28(4):972–99. doi: 10.1111/bjhp.12664 (PMC10947272; doi:10.1111/bjhp.12664)
Supplement: Supplementary file 15 — File S4 [file BJHP-28-972-s013.docx]

**Supplementary File 4**

**Provider Fidelity to Behavior Change Counselling**

**Assessment**

Forty-six studies (79.31%) reported using methods to evaluate the fidelity of intervention delivery: 4/6 (66.67%) substance use(Darker et al., 2016b; Darker et al., 2016a; Garner et al., 2020; Garner, Gotham, et al., 2017; Garner, Zehner, et al., 2017; Jaffray et al., 2014; Mertens et al., 2014); 4/5 (80%) physical activity(Dennett et al., 2018; Elley et al., 2003; O'Halloran et al., 2016; van der Weegen et al., 2015; Renée Verwey et al., 2014; R. Verwey et al., 2014, 2016; Renée Verwey et al., 2016); 7/9 (77.77%) smoking(Borrelli et al., 2005b; Butler et al., 1999; Cabezas et al., 2011; Cabezas et al., 2009; Cossette et al., 2012; Ershoff et al., 1999; Glasgow et al., 2000; Hollis et al., 2007; Rollnick et al., 1997); 6/7 (85.71%) treatment adherence(Cook et al., 2017; E. Drevenhorn et al., 2007; Eva Drevenhorn et al., 2007; Drevenhorn et al., 2012; Drevenhorn et al., 2015; George et al., 2020; George et al., 2021; George et al., 2019; Graham et al., 2016; Hedegaard et al., 2016; Hedegaard et al., 2015; Leiva et al., 2014); 8/11 (72.72%) alcohol(Adams et al., 1998; D'Onofrio et al., 2008; D'Onofrio et al., 2005; Darnell et al., 2016; Dhital et al., 2013; Dhital et al., 2015; Dunn, 2003; Fleming et al., 2010; Greenfield et al., 2010; L'Engle et al., 2014; Ockene et al., 1999; Ockene et al., 1997; Schaus et al., 2009; Shin et al., 2013; Shin et al., 2012; Zatzick et al., 2014; Zatzick et al., 2013); 3/3 (100%) sub-optimal glycaemic control(Ismail et al., 2018; Juul et al., 2011; Juul et al., 2014; Lauffenburger et al., 2019; Lauffenburger et al., 2017; Magill et al., 2018b); 9/11 (81.81%) multiple health behaviors(Bóveda-Fontán et al., 2015; Butler et al., 2013; Heinrich et al., 2010; R. Jansink et al., 2013; Renate Jansink et al., 2013; Jansink et al., 2009; Koelewijn-van Loon et al., 2010; Koelewijn-van Loon et al., 2009; Koelewijn-van Loon et al., 2008; Lakerveld et al., 2012; Lakerveld et al., 2013; Lakerveld et al., 2008; Nanchahal et al., 2012; Noble et al., 2018; Pérula et al., 2011; Pérula et al., 2012; Spanou et al., 2010; van Wier et al., 2013; Verweij et al., 2011; Verweij et al., 2009; Verweij et al., 2012; Whittemore et al., 2009); and 5/6 (83.33%) other health behavior(Beck et al., 2015; Beck et al., 2020; Beck et al., 2017; Britton et al., 2019; Britton et al., 2015; Dermen et al., 2014; Hegarty et al., 2013; Hegarty et al., 2010; Hegarty et al., 2008) focused interventions. Observer rating was the most common method for assessing fidelity of intervention delivery (n=30), with ratings derived from audio-recordings [n=20;(Beck et al., 2015; Beck et al., 2020; Beck et al., 2017; Britton et al., 2019; Britton et al., 2015; Cabezas et al., 2011; Cabezas et al., 2009; Cook et al., 2017; D'Onofrio et al., 2008; D'Onofrio et al., 2005; Darker et al., 2016b; Darker et al., 2016a; Dermen et al., 2014; E. Drevenhorn et al., 2007; Eva Drevenhorn et al., 2007; Drevenhorn et al., 2012; Drevenhorn et al., 2015; Garner et al., 2020; Garner, Gotham, et al., 2017; Garner, Zehner, et al., 2017; George et al., 2020; George et al., 2021; George et al., 2019; Greenfield et al., 2010; Hedegaard et al., 2016; Hedegaard et al., 2015; Hegarty et al., 2013; Hegarty et al., 2010; Hegarty et al., 2008; Hollis et al., 2007; Ismail et al., 2018; Koelewijn-van Loon et al., 2010; Koelewijn-van Loon et al., 2009; Koelewijn-van Loon et al., 2008; Lakerveld et al., 2012; Lakerveld et al., 2013; Lakerveld et al., 2008; Magill et al., 2018b; Mertens et al., 2014; Nanchahal et al., 2012; Noble et al., 2018; Schaus et al., 2009; Shin et al., 2013; Shin et al., 2012; van Wier et al., 2013; Verweij et al., 2011; Verweij et al., 2009; Verweij et al., 2012; Ward et al., 2015)], video-recordings [n=2;(Bóveda-Fontán et al., 2015; R. Jansink et al., 2013; Renate Jansink et al., 2013; Jansink et al., 2009; Pérula et al., 2011; Pérula et al., 2012)] or direct observation [n=2;(Glasgow et al., 2000; L'Engle et al., 2014)] of patient consultations; standardised role-plays/ simulated consultations [n=4;(Butler et al., 2013; Darnell et al., 2016; Dennett et al., 2018; Dunn, 2003; O'Halloran et al., 2016; Spanou et al., 2010; Zatzick et al., 2014; Zatzick et al., 2013)]; or a combination thereof [n=2;(Borrelli et al., 2005b; Heinrich et al., 2010)]. Additional methods included participant recall via follow-up interview [n=8;(Adams et al., 1998; Borrelli et al., 2005b; Butler et al., 2013; Cornman et al., 2008; Dhital et al., 2013; Dhital et al., 2015; Elley et al., 2003; Hegarty et al., 2013; Hegarty et al., 2010; Hegarty et al., 2008; Nanchahal et al., 2012; Noble et al., 2018; Ockene et al., 1999; Ockene et al., 1997; Spanou et al., 2010)] or self-report questionnaire [n=4;(Cornman et al., 2008; Fisher et al., 2004; Fisher et al., 2006; Fisher et al., 2014; Jaffray et al., 2014; Juul et al., 2011; Juul et al., 2014; Schaus et al., 2009)] and provider self-report via checklist [n=10;(Cornman et al., 2008; Ershoff et al., 1999; Fisher et al., 2004; Fisher et al., 2006; Fisher et al., 2014; Fleming et al., 2010; Hegarty et al., 2013; Hegarty et al., 2010; Hegarty et al., 2008; L'Engle et al., 2014; Schaus et al., 2009; van der Weegen et al., 2015; Verweij et al., 2011; Verweij et al., 2009; Verweij et al., 2012; Renée Verwey et al., 2014; R. Verwey et al., 2014, 2016; Renée Verwey et al., 2016; Whittemore et al., 2009)], session notes [n=3;(Borrelli et al., 2005b; Hedegaard et al., 2016; Hedegaard et al., 2015; R. Jansink et al., 2013; Renate Jansink et al., 2013; Jansink et al., 2009)], or follow-up interview/ questionnaire [n=4,(Butler et al., 1999; Hegarty et al., 2013; Hegarty et al., 2010; Hegarty et al., 2008; Juul et al., 2011; Juul et al., 2014; Rollnick et al., 1997; van der Weegen et al., 2015; Renée Verwey et al., 2014; R. Verwey et al., 2014, 2016; Renée Verwey et al., 2016).

Of the 30 studies that reported using an observer rated instrument, six(Beck et al., 2015; Beck et al., 2020; Beck et al., 2017; Britton et al., 2019; Britton et al., 2015; Cabezas et al., 2011; Cabezas et al., 2009; Cook et al., 2017; Darker et al., 2016b; Darker et al., 2016a; Lakerveld et al., 2012; Lakerveld et al., 2013; Lakerveld et al., 2008; Mertens et al., 2014; van Wier et al., 2013; Ward et al., 2015) reported using randomisation to select the sample for fidelity analysis and eight(Beck et al., 2015; Beck et al., 2020; Beck et al., 2017; Britton et al., 2019; Britton et al., 2015; Cook et al., 2017; D'Onofrio et al., 2008; D'Onofrio et al., 2005; Dermen et al., 2014; Garner et al., 2020; Garner, Gotham, et al., 2017; Garner, Zehner, et al., 2017; Ismail et al., 2018; R. Jansink et al., 2013; Renate Jansink et al., 2013; Jansink et al., 2009; Magill et al., 2018b; Nanchahal et al., 2012; Noble et al., 2018) specified that the raters were blind. Of the 16 studies that reported the number of raters(Beck et al., 2015; Beck et al., 2020; Beck et al., 2017; Bóveda-Fontán et al., 2015; Britton et al., 2019; Britton et al., 2015; Cook et al., 2017; D'Onofrio et al., 2008; D'Onofrio et al., 2005; Darker et al., 2016b; Darker et al., 2016a; Darnell et al., 2016; Dunn, 2003; Garner et al., 2020; Garner, Gotham, et al., 2017; Garner, Zehner, et al., 2017; Greenfield et al., 2010; Hedegaard et al., 2016; Hedegaard et al., 2015; Heinrich et al., 2010; Ismail et al., 2018; Lakerveld et al., 2012; Lakerveld et al., 2013; Lakerveld et al., 2008; Magill et al., 2018b; Mertens et al., 2014; Nanchahal et al., 2012; Noble et al., 2018; O'Halloran et al., 2016; Pérula et al., 2011; Pérula et al., 2012; Shin et al., 2013; Shin et al., 2012; van Wier et al., 2013; Verweij et al., 2011; Verweij et al., 2009; Verweij et al., 2012; Ward et al., 2015; Zatzick et al., 2014; Zatzick et al., 2013), most used more than one rater [12/16, 75%;(Bóveda-Fontán et al., 2015; Cook et al., 2017; D'Onofrio et al., 2008; D'Onofrio et al., 2005; Darnell et al., 2016; Dunn, 2003; Garner et al., 2020; Garner, Gotham, et al., 2017; Garner, Zehner, et al., 2017; Greenfield et al., 2010; Hedegaard et al., 2016; Hedegaard et al., 2015; Heinrich et al., 2010; Ismail et al., 2018; Lakerveld et al., 2012; Lakerveld et al., 2013; Lakerveld et al., 2008; Magill et al., 2018b; Nanchahal et al., 2012; Noble et al., 2018; Pérula et al., 2011; Pérula et al., 2012; Shin et al., 2013; Shin et al., 2012; van Wier et al., 2013; Zatzick et al., 2014; Zatzick et al., 2013)]. However the assessment and reporting of inter-rater reliability was poor. Nine studies reported the total number of consultations rated(Beck et al., 2015; Beck et al., 2020; Beck et al., 2017; Britton et al., 2019; Britton et al., 2015; D'Onofrio et al., 2008; D'Onofrio et al., 2005; Darnell et al., 2016; Dermen et al., 2014; E. Drevenhorn et al., 2007; Eva Drevenhorn et al., 2007; Drevenhorn et al., 2012; Drevenhorn et al., 2015; Dunn, 2003; Heinrich et al., 2010; Ismail et al., 2018; R. Jansink et al., 2013; Renate Jansink et al., 2013; Jansink et al., 2009; Magill et al., 2018b; Nanchahal et al., 2012; Noble et al., 2018; Zatzick et al., 2014; Zatzick et al., 2013), with studies reporting a range of between 22(Nanchahal et al., 2012; Noble et al., 2018) and 392(D'Onofrio et al., 2008; D'Onofrio et al., 2005) consultations. Nineteen studies (63.3%) reported using one or more validated instruments, including the MITI [n=8;(Darnell et al., 2016; Dennett et al., 2018; Dermen et al., 2014; Dunn, 2003; Ismail et al., 2018; R. Jansink et al., 2013; Renate Jansink et al., 2013; Jansink et al., 2009; Lakerveld et al., 2012; Lakerveld et al., 2013; Lakerveld et al., 2008; Magill et al., 2018b; Nanchahal et al., 2012; Noble et al., 2018; O'Halloran et al., 2016; van Wier et al., 2013; Zatzick et al., 2014; Zatzick et al., 2013)]; BECCI [n=6;(Beck et al., 2015; Beck et al., 2020; Beck et al., 2017; Britton et al., 2019; Britton et al., 2015; Butler et al., 2013; Ismail et al., 2018; R. Jansink et al., 2013; Renate Jansink et al., 2013; Jansink et al., 2009; Magill et al., 2018b; Mertens et al., 2014; Spanou et al., 2010; Verweij et al., 2011; Verweij et al., 2009; Verweij et al., 2012; Ward et al., 2015)]; BNI adherence and competence scale [n=2;(D'Onofrio et al., 2008; D'Onofrio et al., 2005; George et al., 2020; George et al., 2021; George et al., 2019)]; MISC [n=2;(Cook et al., 2017; Lakerveld et al., 2012; Lakerveld et al., 2013; Lakerveld et al., 2008; van Wier et al., 2013)]; Independent Tape Rater Scale [n=1;(Garner et al., 2020; Garner, Gotham, et al., 2017; Garner, Zehner, et al., 2017)] and EVEM [n=1;(Bóveda-Fontán et al., 2015; Pérula et al., 2011; Pérula et al., 2012)].

**Reporting**

Fidelity outcome data was subsequently reported by 28/46 (60%) studies: 2/4 (50%) substance use(Garner et al., 2020; Garner, Gotham, et al., 2017; Garner, Zehner, et al., 2017; Jaffray et al., 2014); 2/4 (50%) physical activity(Elley et al., 2003; van der Weegen et al., 2015; Renée Verwey et al., 2014; R. Verwey et al., 2014, 2016; Renée Verwey et al., 2016); 3/7 (42%) smoking(Butler et al., 1999; Cossette et al., 2012; Glasgow et al., 2000; Rollnick et al., 1997); 5/6 (83%) treatment adherence(Cook et al., 2017; E. Drevenhorn et al., 2007; Eva Drevenhorn et al., 2007; Drevenhorn et al., 2012; Drevenhorn et al., 2015; George et al., 2020; George et al., 2021; George et al., 2019; Hedegaard et al., 2016; Hedegaard et al., 2015; Leiva et al., 2014); 5/8 (62%) alcohol(Adams et al., 1998; D'Onofrio et al., 2008; D'Onofrio et al., 2005; Darnell et al., 2016; Dhital et al., 2013; Dhital et al., 2015; Dunn, 2003; Greenfield et al., 2010; Ockene et al., 1999; Ockene et al., 1997; Shin et al., 2013; Shin et al., 2012; Zatzick et al., 2014; Zatzick et al., 2013); 2/3 (66%) sub-optimal glycaemic control(Ismail et al., 2018; Juul et al., 2011; Juul et al., 2014; Magill et al., 2018b); 7/9 (77%) multiple health behaviors(Bóveda-Fontán et al., 2015; Butler et al., 2013; R. Jansink et al., 2013; Renate Jansink et al., 2013; Jansink et al., 2009; Lakerveld et al., 2012; Lakerveld et al., 2013; Lakerveld et al., 2008; Nanchahal et al., 2012; Noble et al., 2018; Pérula et al., 2011; Pérula et al., 2012; Spanou et al., 2010; van Wier et al., 2013; Verweij et al., 2011; Verweij et al., 2009; Verweij et al., 2012; Whittemore et al., 2009); and 2/5 (40%) other health behaviors(Beck et al., 2015; Beck et al., 2020; Beck et al., 2017; Britton et al., 2019; Britton et al., 2015; Cornman et al., 2008). Outcomes were often idiosyncratic (e.g. derived from a study checklist of intervention components). Less than half assessed [n=20;(Adams et al., 1998; Beck et al., 2015; Beck et al., 2020; Beck et al., 2017; Bóveda-Fontán et al., 2015; Britton et al., 2019; Britton et al., 2015; Butler et al., 2013; Cook et al., 2017; D'Onofrio et al., 2008; D'Onofrio et al., 2005; Darnell et al., 2016; Dermen et al., 2014; Dhital et al., 2013; Dhital et al., 2015; Dunn, 2003; Elley et al., 2003; George et al., 2020; George et al., 2021; George et al., 2019; Glasgow et al., 2000; Greenfield et al., 2010; Hegarty et al., 2013; Hegarty et al., 2010; Hegarty et al., 2008; Hollis et al., 2007; Ismail et al., 2018; Jaffray et al., 2014; R. Jansink et al., 2013; Renate Jansink et al., 2013; Jansink et al., 2009; Juul et al., 2011; Juul et al., 2014; Magill et al., 2018b; Ockene et al., 1999; Ockene et al., 1997; Pérula et al., 2011; Pérula et al., 2012; Shin et al., 2013; Shin et al., 2012; Spanou et al., 2010; Zatzick et al., 2014; Zatzick et al., 2013)], and even fewer made comparisons between [n=12;(Adams et al., 1998; Beck et al., 2015; Beck et al., 2020; Beck et al., 2017; Britton et al., 2019; Britton et al., 2015; Butler et al., 2013; D'Onofrio et al., 2008; D'Onofrio et al., 2005; Darnell et al., 2016; Dhital et al., 2013; Dhital et al., 2015; Dunn, 2003; Ismail et al., 2018; Jaffray et al., 2014; R. Jansink et al., 2013; Renate Jansink et al., 2013; Jansink et al., 2009; Juul et al., 2011; Juul et al., 2014; Magill et al., 2018b; Ockene et al., 1999; Ockene et al., 1997; Spanou et al., 2010; Zatzick et al., 2014; Zatzick et al., 2013)] the delivery of intervention and comparison conditions (i.e. differentiation).

Provider fidelity to the specific principles or strategies of BCC (e.g. MI) using a validated instrument was reported by 11/28 (39.28%) studies: substance use: [n=1/2, 50%;(Garner et al., 2020; Garner, Gotham, et al., 2017; Garner, Zehner, et al., 2017)]; physical activity (0/3); smoking (0/5); treatment adherence: [n=1/5, 20%);(Cook et al., 2017)]; alcohol: [n=1/5; 20%);(Darnell et al., 2016; Dunn, 2003; Zatzick et al., 2014; Zatzick et al., 2013)]; suboptimal glycaemic control: [n=1/2, 50%;(Ismail et al., 2018; Magill et al., 2018b)]; multiple health behavior: [n=6/7, 85%;(Bóveda-Fontán et al., 2015; Butler et al., 2013; R. Jansink et al., 2013; Renate Jansink et al., 2013; Jansink et al., 2009; Lakerveld et al., 2012; Lakerveld et al., 2013; Lakerveld et al., 2008; Nanchahal et al., 2012; Noble et al., 2018; Pérula et al., 2011; Pérula et al., 2012; Spanou et al., 2010; van Wier et al., 2013; Verweij et al., 2011; Verweij et al., 2009; Verweij et al., 2012)]; and other: [n=1/2, 50%;(Beck et al., 2015; Beck et al., 2020; Beck et al., 2017; Britton et al., 2019; Britton et al., 2015)] health behavior focused interventions. Findings were derived from one or more of the following instruments: MITI [(n=4;(Darnell et al., 2016; Dunn, 2003; Ismail et al., 2018; Lakerveld et al., 2012; Lakerveld et al., 2013; Lakerveld et al., 2008; Magill et al., 2018b; Nanchahal et al., 2012; Noble et al., 2018; van Wier et al., 2013; Zatzick et al., 2014; Zatzick et al., 2013)]; BECCI [n=5;(Beck et al., 2015; Beck et al., 2020; Beck et al., 2017; Britton et al., 2019; Britton et al., 2015; Butler et al., 2013; Ismail et al., 2018; R. Jansink et al., 2013; Renate Jansink et al., 2013; Jansink et al., 2009; Magill et al., 2018b; Spanou et al., 2010; Verweij et al., 2011; Verweij et al., 2009; Verweij et al., 2012)]; MISC [n=2;(Cook et al., 2017; Lakerveld et al., 2012; Lakerveld et al., 2013; Lakerveld et al., 2008; van Wier et al., 2013)]; EVEM [n=1;(Bóveda-Fontán et al., 2015; Pérula et al., 2011; Pérula et al., 2012)] and Independent Tape Rater Scale [(n=1;(Garner et al., 2020; Garner, Gotham, et al., 2017; Garner, Zehner, et al., 2017)]. From the findings presented, average BECCI scores for intervention providers ranged from *M*=1.196 [(*SD*=not reported;(Butler et al., 2013; Spanou et al., 2010)] to *M*=2.14 [*SD*=0.42;(Beck et al., 2015; Beck et al., 2020; Beck et al., 2017; Britton et al., 2019; Britton et al., 2015)] whereby 1= ‘minimally’ and 2= ‘to some extent’. On the MITI, % MI adherence scores reported for intervention providers ranged from *M*=53.1 [*SD*=17.2;(Nanchahal et al., 2012; Noble et al., 2018)] to *M*=94.5 [*Range*=87-100;(Lakerveld et al., 2012; Lakerveld et al., 2013; Lakerveld et al., 2008; van Wier et al., 2013)] whereby ‘beginning proficiency’ = 90.
